# Asymmetric Synthesis of Saturated and Unsaturated Hydroxy Fatty Acids (HFAs) and Study of Their Antiproliferative Activity

**DOI:** 10.3390/biom14010110

**Published:** 2024-01-15

**Authors:** Olga G. Mountanea, Christiana Mantzourani, Dimitrios Gkikas, Panagiotis K. Politis, George Kokotos

**Affiliations:** 1 Department of Chemistry, National and Kapodistrian University of Athens, 15771 Athens, Greece; olgamount@chem.uoa.gr (O.G.M.); chrmantz@chem.uoa.gr (C.M.); 2 Center of Excellence for Drug Design and Discovery, National and Kapodistrian University of Athens, 15771 Athens, Greece; 3 Center for Basic Research, Biomedical Research Foundation of the Academy of Athens, 4 Soranou Efesiou Str., 11527 Athens, Greece; dgkikas@bioacademy.gr (D.G.); ppolitis@bioacademy.gr (P.K.P.); 4 School of Medicine, European University Cyprus, Nicosia 2404, Cyprus

**Keywords:** antiproliferative activity, asymmetric synthesis, hydroxy fatty acids, hydroxypalmitic acid, hydroxystearic acid

## Abstract

Hydroxy fatty acids (HFAs) constitute a class of lipids, distinguished by the presence of a hydroxyl on a long aliphatic chain. This study aims to expand our insights into HFA bioactivities, while also introducing new methods for asymmetrically synthesizing unsaturated and saturated HFAs. Simultaneously, a procedure previously established by us was adapted to generate new HFA regioisomers. An organocatalytic step was employed for the synthesis of chiral terminal epoxides, which either by alkynylation or by Grignard reagents resulted in unsaturated or saturated chiral secondary alcohols and, ultimately, HFAs. 7-(*S*)-Hydroxyoleic acid (7SHOA), 7-(*S*)-hydroxypalmitoleic acid (7SHPOA) and 7-(*R*)- and (*S*)-hydroxymargaric acids (7HMAs) were synthesized for the first time and, together with regioisomers of (*R*)- and (*S*)-hydroxypalmitic acids (HPAs) and hydroxystearic acids (HSAs), whose biological activity has not been tested so far, were studied for their antiproliferative activities. The unsaturation of the long chain, as well as an odd-numbered (C17) fatty acid chain, led to reduced activity, while the new 6-(*S*)-HPA regioisomer was identified as exhibiting potent antiproliferative activity in A549 cells. 6SHPA induced acetylation of histone 3 in A549 cells, without affecting acetylated α-tubulin levels, suggesting the selective inhibition of histone deacetylase (HDAC) class I enzymes, and was found to inhibit signal transducer and activator of transcription 3 (STAT3) expression.

## 1. Introduction

**Figure 1:**
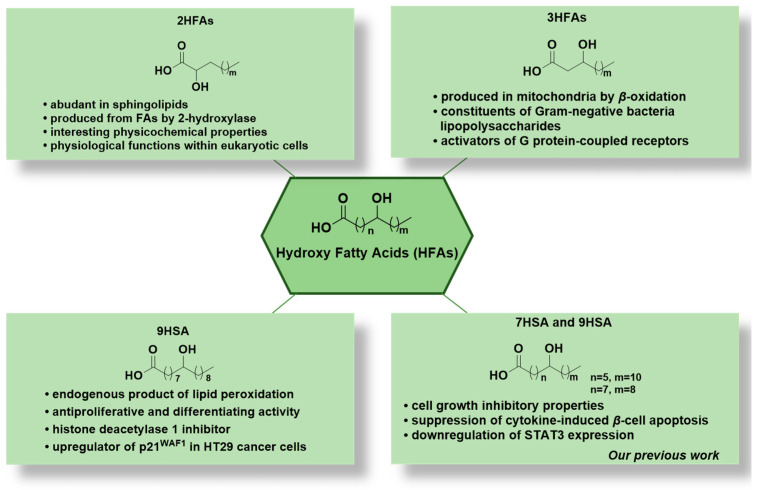
Saturated hydroxy fatty acids (SHFAs) and their roles.

Among the diverse fatty acids (FAs), hydroxy fatty acids (HFAs) constitute a unique class, which is characterized by the presence of a hydroxyl group attached to a long aliphatic chain. Although they comprise a relatively small class of lipids, HFAs have attracted attention, due to their importance as components of animal and plant tissues, engaging in diverse biological functions [1]. They are classified by the position of the hydroxyl functionality (defined by the number of the carbon atom, which carries the hydroxyl, or a Greek letter such as *α*, *β*, or *ω*, *ω*-1, etc.), the number of hydroxyl groups (mono, di- or poly) and the nature of the long chain. Special attention has been paid to 2-hydroxy fatty acids (2HFAs) and 3-hydroxy fatty acids (3HFAs) (Figure 1), which are widespread in nature, being involved in oxidative bio-transformations in animal and plant organisms. 2HFAs are found as components of sphingolipids, produced by the 2-hydroxylation of FAs [2], whereas 3HFAs are present in mitochondria, generated by fatty acid β-oxidation [3], and are components of inflammatory lipopolysaccharides [4].

Three years ago, adopting a “suspect” analysis approach, we identified the presence of families of hydroxylated palmitic and stearic acids (HPAs and HSAs, respectively) in cow milk, human plasma and yogurt [5,6,7] (Figure 1). HPAs and HSAs carrying a hydroxyl at the 7- or 9-positions (7HPA, 9HPA, 7HSA, 9HSA) were found to exhibit antiproliferative activities against human cancer cell lines (A549, Caco-2, and SF268 cells) [5]. Furthermore, 7HSA and 9HSA were demonstrated to suppress β-cell apoptosis induced by proinflammatory cytokines [5]. As a matter of fact, 9HSA was first identified in Lewis lung carcinoma cells, in 1991, as an oxidation product [8]. Later on, it was demonstrated that 9HSA was able to upregulate p21^WAF1^ in HT29 cancer cells [9], to inhibit the cell growth in human colon cancer through histone deacetylase 1 [10], and to interfere with EGF signaling in a human colon adenocarcinoma [11].

In our previous work, we synthesized 7-, 9-, 10-HSAs and 7-, 9-, 10-HPAs and studied their inhibitory effect on cancer cell growth [5]. At the same time, Calonghi et al. synthesized derivatives of 9HSA and studied their antiproliferative activity on HT29 cancer cells [12]. Most recently, Calonghi et al. studied the effect of regioisomerism on the antiproliferative activity of HSAs on human cancer cell lines and reported that 5HSA not only presented antiproliferative activity, but also induced changes in cell displacement, directionality and speed [13]. To extend our knowledge on HFA bioactivities and to complete structure–activity relationship studies, we decided to synthesize chiral HPAs and HSAs carrying a hydroxyl group at the 6-, 8- and 11-positions and (*R*)- and (*S*)-7-hydroxymargaric acids (7HMAs), as well as unsaturated 7-hydroxyoleic acid and 7-hydroxypalmitoleic acid, which have not previously been prepared and tested. In this work, we present the synthesis of various regioisomers of HPAs, HSAs, HMAs and unsaturated HFAs with 16C and 18C carbon chains, as well as the study of their antiproliferative activity on cancer cell lines.

## 2. Materials and Methods

### 2.1. General Remarks

All commercially available products and solvents were purchased from Fluorochem (Fluorochem Ltd., Hadfield, Glossop, UK) Sigma-Aldrich (Sigma-Aldrich, Saint Louis, MO, USA), Fluka (Fluka Chemicals Ltd., Gillingham, Dorset, UK), Merck (Merck, Darmstadt, Germany), and Alfa Aesar (Alfa Aesar, Ward Hill, MA, USA). Solvents were used as received or dried over molecular sieves (4 Å). All water- or air-sensitive reactions were performed under an argon atmosphere, employing dry solvents and anhydrous conditions. Chromatographic purification of products was accomplished using forced-flow chromatography on Merck^®^ (Merck, Darmstadt, Germany) Kieselgel 60 F254 230–400 mesh. Thin-layer chromatography (TLC) was performed on aluminum-backed silica plates (0.2 mm, 60 F254). Visualization of the developed chromatogram was performed by fluorescence quenching using phosphomolybdic acid. Melting points were measured on a Buchi 530 apparatus (Buchi, Flawil, Switzerland) and are uncorrected. ^1^H NMR and ^13^C NMR spectra were recorded on a Varian Mercury (Varian, Palo Alto, CA, USA) (200 and 50 MHz, respectively) or an Avance III HD Bruker 400 MHz (Bruker, Fällanden, Switzerland) (400 MHz and 100 MHz, respectively) and are internally referenced to residual solvent signals. Data for ^1^H NMR are reported as follows: chemical shift (*δ* ppm), integration, multiplicity (s = singlet, d = doublet, t = triplet, m = multiplet, br s = broad signal), coupling constant and assignment. Data for ^13^C NMR are reported in terms of chemical shift (*δ* ppm). Optical rotations were measured using a PerkinElmer 343 (PerkinElmer, Shelton, Connecticut, USA) or an AA-65 series polarimeter (Optical Activity Ltd., Bury, UK) in a 10 cm cell at room temperature. Mass spectra (ESI) were recorded on a Finningan^®^ Surveyor MSQ LC–MS spectrometer (Thermo Finnigan, Co. Ltd., San Jose, CA, USA). High-resolution mass spectra were obtained on a Bruker Maxis Impact QTOF spectrometer (Bruker Daltonics, Bremen, Germany) or an AB Sciex 4600 Triple TOF mass spectrometer (AB Sciex, Singapore). The enantiomeric excess (*ee*) of compounds (*S*)- and (*R*)-**11a**–**d** was determined by HPLC analysis performed on an Agilent 1100 Series (Agilent Co., Santa Clara, CA, USA) with a DAD UV detector, and the peak intensities were measured in the UV range between 206 and 280 nm. A Daicel Chiralpak OD-H chromatography column (250 × 4.6 mm ID) was used. HPLC-grade hexane and ^i^PrOH were used as solvents in a *n*-hexane:^i^PrOH 99:1 ratio and a flow rate of 1 mL·min^–1^.

### 2.2. Synthesis of 8-((Tert-butyldimethylsilyl)oxy)octanal (***2***) Using Pyridinium Chlorochromate (PCC)

Monoprotected diol **1** (1.00 mmol) was dissolved in dry CH_2_Cl_2_ (5 mL) and the solution was added to a stirred solution of PCC (431 mg, 2.00 mmol) in dry CH_2_Cl_2_ (10 mL) at 0 °C. The reaction mixture was left stirring at room temperature for 1 h. Then, the reaction mixture was filtered through a funnel packed with celite and silica and the residue was washed with CH_2_Cl_2_ (30 mL). The filtrate was concentrated and evaporated to give a crude mixture, which was purified by flash chromatography on silica gel, eluting with petroleum ether (bp 40−60 °C):ethyl acetate (90:10–80:20) to give the desired mono-protected aldehyde **2** [14]. Colorless oil; yield 85%; ^1^H NMR (200 MHz, CDCl_3_): *δ* 9.74 (1H, t, *J* = 1.9 Hz, CHO), 3.58 (2H, t, *J* = 6.4 Hz, CH_2_OTBDMS), 2.46–2.28 (2H, m, C*H*_2_CHO), 1.69–1.42 (4H, m, 2 × CH_2_), 1.38–1.22 (6H, m, 3 × CH_2_), 0.87 (9H, s, 3 × CH_3_), 0.03 (6H, s, 2 × CH_3_); ^13^C NMR (50 MHz, CDCl_3_): *δ* 202.8, 63.1, 43.8, 32.7, 29.1, 25.9, 25.6, 22.0, 18.3, −5.3; MS 281 [M + Na]^+^.

### 2.3. General Procedure for the Synthesis of Chiral Epoxides Using MacMillan’s Imidazolidinone

To a round bottom flask, (2*S*,5*R*)-2-(*tert*-butyl)-3,5-dimethylimidazolidin-4-one trifluoroacetate (for *S*-epoxide synthesis) or (2*R*,5*S*)-2-(*tert*-butyl)-3,5-dimethylimidazolidin-4-one trifluoroacetate (for *R*-epoxide synthesis) (57 mg, 0.20 mmol) was dissolved in THF (0.5 mL) and an addition of 2,3,4,5,6,6-hexachlorocyclohexa-2,4-dien-1-one (331 mg, 1.10 mmol) followed. After vigorous stirring for 5 min, the corresponding aldehyde (1.00 mmol) was added to the reaction mixture. Following an additional 20 min of stirring at room temperature, the resultant mixture was subsequently cooled to 0 °C, before a solution of NaBH_4_ (95 mg, 2.50 mmol) in EtOH (1 mL) was added. After a duration of 10 min, the reaction mixture was allowed to attain room temperature over a 5 min period. Eventually, a freshly prepared solution containing aqueous KOH (1.70 g KOH diluted in 2.7 mL of water) and EtOH (1.3 mL) was introduced. The resultant reaction mixture underwent vigorous stirring for a duration of 30 min, and, then, H_2_O (20 mL) was added. The reaction mixture was extracted with Et_2_O (3 × 20 mL), washed with brine (1 × 20 mL), dried over Na_2_SO_4_, filtered, and concentrated in vacuo. The desired epoxide was isolated through purification using silica gel flash chromatography, eluting with petroleum ether (bp 40−60 °C):ethyl acetate (80:20–70:30).

(*S*)-*tert*-Butyldimethyl((6-(oxiran-2-yl)hexyl)oxy)silane (**3**). Colorless oil; yield 74%; [α]_D_^20^ = −2.5 (c 2.0, CH_2_Cl_2_); ^1^H NMR (200 MHz, CDCl_3_): *δ* 3.55 (2H, t, *J* = 6.2 Hz, CH_2_OTBDMS), 2.90–2.79 (1H, m, OCH), 2.68 (1H, t, *J* = 4.3 Hz, OC*H*H), 2.40 (1H, dd, *J* = 4.3 and 2.5 Hz, OC*H*H), 1.52–1.26 (10H, m, 5 × CH_2_), 0.84 (9H, s, 3 × CH_3_), −0.01 (6H, s, 2 × CH_3_); ^13^C NMR (50 MHz, CDCl_3_): *δ* 63.0, 52.2, 46.9, 32.6, 32.3, 29.1, 25.9, 25.6, 18.2, −5.4; HRMS (ESI^+^): *m*/*z* calculated for C_14_H_31_O_2_Si^+^: 259.2088; [M + H]^+^ found: 259.2086.

(*S*)-2-(5-(Benzyloxy)pentyl)oxirane (*S*-**11a**). Colorless oil; yield 80%; [α]_D_^20^ = −3.5 (c 1.0, CH_2_Cl_2_); ^1^H NMR (200 MHz, CDCl_3_): *δ* 7.46–7.13 (5H, m, ArH), 4.50 (2H, s, PhCH_2_O), 3.48 (2H, t, *J* = 6.4 Hz, OCH_2_), 2.97–2.86 (1H, m, OCH), 2.76 (1H, t, *J* = 4.4 Hz, OC*H*H), 2.48 (1H, dd, *J* = 4.4 and 2.7 Hz, OC*H*H), 1.72–1.36 (8H, m, 4 × CH_2_); ^13^C NMR (50 MHz, CDCl_3_): *δ* 138.3, 128.2, 127.5, 127.4, 72.8, 70.1, 52.4, 47.1, 32.3, 29.5, 25.9, 25.7; HRMS (ESI^+^): *m*/*z* calculated for C_14_H_20_NaO_2_^+^: 243.1356; [M + Na]^+^ found: 243.1357; HPLC analysis: 95% *ee*.

(*R*)-2-(5-(Benzyloxy)pentyl)oxirane (*R*-**11a**). Colorless oil; yield 78%; [α]_D_^20^ = +4.5 (c 2.0, CH_2_Cl_2_); HPLC analysis: 90% *ee*.

(*S*)-2-(6-(Benzyloxy)hexyl)oxirane (*S*-**11b**) [5]. Colorless oil; yield 75%; [α]_D_^20^ = −5.0 (c 1.0, CH_2_Cl_2_), [α]_D_^20^ lit. = −5.0 (c 1.0, CH_2_Cl_2_); ^1^H NMR (200 MHz, CDCl_3_): *δ* 7.45–7.14 (5H, m, ArH), 4.50 (2H, s, PhCH_2_O), 3.46 (2H, t, *J* = 6.5 Hz, OCH_2_), 2.94–2.80 (1H, m, OCH), 2.73 (1H, t, *J* = 4.5 Hz, OC*H*H), 2.45 (1H, dd, *J* = 4.5 and 2.7 Hz, OC*H*H), 1.65–1.22 (10H, m, 5 × CH_2_); ^13^C NMR (50 MHz, CDCl_3_): *δ* 138.5, 128.3, 127.5, 127.4, 72.8, 70.3, 52.3, 47.0, 32.3, 29.6, 29.2, 26.0, 25.9; HRMS (ESI^+^): *m*/*z* calculated for C_15_H_22_NaO_2_^+^: 257.1512; [M + Na]^+^ found: 257.1512; HPLC analysis: 93% *ee*.

(*R*)-2-(6-(Benzyloxy)hexyl)oxirane (*R*-**11b**) [15]. Colorless oil; yield 80%; [α]_D_^20^ = +5.4 (c 1.0, CH_2_Cl_2_), [α]_D_^25^ lit. = +5.6 (c 2.0, CHCl_3_); HPLC analysis: 90% *ee*.

(*S*)-2-(7-(Benzyloxy)heptyl)oxirane (*S*-**11c**) [16]. Colorless oil; yield 80%; [α]_D_^20^ = −6.5 (c 1.0, CH_2_Cl_2_), [α]_D_^20^ lit. = −6.7 (c 1.0, CHCl_3_); ^1^H NMR (200 MHz, CDCl_3_): *δ* 7.44–7.19 (5H, m, ArH), 4.51 (2H, s, PhCH_2_O), 3.48 (2H, t, *J* = 6.5 Hz, OCH_2_), 2.97–2.87 (1H, m, OCH), 2.76 (1H, t, *J* = 4.5 Hz, OC*H*H), 2.48 (1H, dd, *J* = 4.5 and 2.9 Hz, OC*H*H), 1.70–1.26 (12H, m, 6 × CH_2_); ^13^C NMR (50 MHz, CDCl_3_): *δ* 138.4, 128.2, 127.5, 127.4, 72.8, 70.3, 52.4, 47.1, 32.3, 29.6, 29.3, 26.0, 25.8; HRMS (ESI^+^): *m*/*z* calculated for C_16_H_24_NaO_2_^+^: 271.1669; [M + Na]^+^ found: 271.1679; HPLC analysis: 92% *ee*.

(*R*)-2-(7-(Benzyloxy)heptyl)oxirane (*R*-**11c**) [17]. Colorless oil; yield 78%; [α]_D_^20^ = +5.0 (c 1.0, CH_2_Cl_2_), [α]_D_^22^ lit. = +4.5 (c 0.92, CHCl_3_); HPLC analysis: 93% *ee*.

(*S*)-2-(10-(Benzyloxy)decyl)oxirane (*S*-**11d**). Colorless oil; yield 75%; [α]_D_^20^ = −3.0 (c 1.0, CHCl_3_); ^1^H NMR (400 MHz, CDCl_3_): *δ* 7.47–7.19 (5H, m, ArH), 4.50 (2H, s, PhCH_2_O), 3.46 (2H, t, *J* = 6.6 Hz, OCH_2_), 3.00–2.83 (1H, m, OCH), 2.74 (1H, t, *J* = 4.5 Hz, OC*H*H), 2.46 (1H, dd, *J* = 4.5 and 2.7 Hz, OC*H*H), 1.65–1.27 (18H, m, 9 × CH_2_); ^13^C NMR (100 MHz, CDCl_3_): *δ* 138.7, 128.3, 127.6, 127.4, 72.8, 70.5, 52.4, 47.1, 32.5, 29.8, 29.5, 29.5, 29.4, 29.4, 26.2, 25.9; HRMS (ESI^+^): *m*/*z* calculated for C_19_H_30_NaO_2_^+^: 313.2138; [M + Na]^+^ found: 313.2139; HPLC analysis: 90% *ee*.

(*R*)-2-(10-(Benzyloxy)decyl)oxirane (*R*-**11d**) [18]. Colorless oil; yield 78%; [α]_D_^20^ = +2.5 (c 1.0, CHCl_3_), [α]_D_^25^ lit. = +2.3 (c 0.5, CHCl_3_); HPLC analysis: 91% *ee*.

(*S*)-*tert*-Butyldimethyl((5-(oxiran-2-yl)pentyl)oxy)silane (**25**) [19]. Colorless oil; yield 76%; [α]_D_^20^ = −3.0 (c 1.0, CHCl_3_), [α]_D_^25^ lit. = −2.36 (c 0.95, CHCl_3_); ^1^H NMR (400 MHz, CDCl_3_): *δ* 3.61 (2H, t, *J* = 6.4 Hz, CH_2_OTBDMS), 2.96–2.86 (1H, m, OCH), 2.74 (1H, t, *J* = 4.3 Hz, OC*H*H), 2.50–2.42 (1H, m, OC*H*H), 1.58–1.38 (8H, m, 4 × CH_2_), 0.89 (9H, s, 3 × CH_3_), 0.04 (6H, s, 2 × CH_3_); ^13^C NMR (100 MHz, CDCl_3_): *δ* 63.1, 52.3, 47.1, 32.7, 32.5, 26.0, 25.8, 25.7, 18.4, −5.3; HRMS (ESI^+^): *m*/*z* calculated for C_13_H_28_NaO_2_Si^+^: 267.1751; [M + Na]^+^ found: 267.1754.

(*R*)-*tert*-Butyldimethyl((5-(oxiran-2-yl)pentyl)oxy)silane (**18**) [20]. Colorless oil; yield 74%; [α]_D_^20^ = +4.0 (c 1.0, CHCl_3_), [α]_D_^21^.^3^ lit. = +4.2 (c 0.9, CHCl_3_).

### 2.4. General Procedure for the Synthesis of Secondary Alcohols from Terminal Epoxides Using Alkynes

To a flame-dried flask under an argon atmosphere, a solution of 1-decyne or 1-octyne or 1-undecyne (0.72 mL or 0.59 mL or 0.79 mL, 4.00 mmol) in anhydrous THF (1.1 mL) was added and the mixture was cooled at −78 °C. Then, the slow addition of *n*-BuLi (1.6 M solution in hexanes, 2.2 mL, 3.50 mmol) over 10 min followed and the reaction mixture was left stirring at −78 °C for 25 min, before BF_3_·OEt_2_ (0.38 mL, 3.00 mmol) and epoxide **3**, **18** or **25** (1.00 mmol) in anhydrous THF (3 mL) were added dropwise. The reaction mixture was left stirring for another 1 h at −78 °C, and then warmed to room temperature. Subsequently, a saturated aqueous solution of NaHCO_3_ (10 mL) was added, and the aqueous layer was extracted with Et_2_O (3 × 20 mL). The combined organic layers were washed with brine (1 × 50 mL), dried over Na_2_SO_4_, filtered, and concentrated under reduced pressure. The crude residue was purified by flash column chromatography eluting with petroleum ether (bp 40−60 °C):diethyl ether (95:5–90:10) to give the expected propargylic alcohol.

(*S*)-1-((*tert*-Butyldimethylsilyl)oxy)octadec-9-yn-7-ol (**4a**). Colorless oil; yield 82%; [α]_D_^20^ = +1.5 (c 2.5, CH_2_Cl_2_); ^1^H NMR (200 MHz, CDCl_3_): *δ* 3.73–3.63 (1H, m, OCH), 3.58 (2H, t, *J* = 6.4 Hz, CH_2_OTBDMS), 2.45–2.26 (2H, m, CH_2_C≡), 2.19–2.11 (2H, m, 2 × C*H*HC≡), 1.97 (1H, d, *J* = 4.8 Hz, OH), 1.53–1.23 (22H, m, 11 × CH_2_), 0.91–0.82 (12H, m, 4 × CH_3_), 0.03 (6H, s, 2 × CH_3_); ^13^C NMR (50 MHz, CDCl_3_): *δ* 83.3, 76.0, 70.2, 63.2, 36.2, 32.8, 31.8, 29.4, 29.2, 29.1, 29.0, 28.9, 27.8, 26.0, 25.8, 25.7, 22.6, 18.7, 18.3, 14.1, −5.3; HRMS (ESI^+^): *m*/*z* calculated for C_24_H_48_NaO_2_Si^+^: 419.3316; [M + Na]^+^ found: 419.3315.

(*S*)-1-((*tert*-Butyldimethylsilyl)oxy)hexadec-9-yn-7-ol (**4b**). Colorless oil; yield 86%; [α]_D_^20^ = +1.0 (c 2.0, CH_2_Cl_2_); ^1^H NMR (200 MHz, CDCl_3_): *δ* 3.69–3.58 (1H, m, OCH), 3.54 (2H, t, *J* = 6.4 Hz, CH_2_OTBDMS), 2.31–2.06 (5H, m, 2 × CH_2_C≡ and OH), 1.52–1.16 (18H, m, 9 × CH_2_), 0.90–0.66 (12H, m, 4 × CH_3_), −0.02 (6H, s, 2 × CH_3_); ^13^C NMR (50 MHz, CDCl_3_): *δ* 82.9, 76.0, 70.0, 63.1, 36.0, 32.7, 31.2, 29.3, 28.9, 28.4, 27.6, 25.8, 25.7, 25.5, 22.4, 18.6, 18.2, 13.9, −5.4; HRMS (ESI^+^): *m*/*z* calculated for C_22_H_45_O_2_Si^+^: 369.3183; [M + H]^+^ found: 369.3184.

(*R*)-1-((*tert*-Butyldimethylsilyl)oxy)octadec-8-yn-6-ol (**19**). Colorless oil; yield 85%; [α]_D_^20^ = +1.8 (c 1.0, CH_2_Cl_2_); ^1^H NMR (400 MHz, CDCl_3_): *δ* 3.72–3.64 (1H, m, OCH), 3.60 (2H, t, *J* = 6.5 Hz, CH_2_OTBDMS), 2.44–2.23 (2H, m, CH_2_C≡), 2.19–2.14 (2H, m, 2 × C*H*HC≡), 1.98–1.91 (1H, m, OH), 1.53–1.48 (4H, m, 2 × CH_2_), 1.39–1.24 (18H, m, 9 × CH_2_), 0.93–0.85 (12H, m, 4 × CH_3_), 0.04 (6H, s, 2 × CH_3_); ^13^C NMR (100 MHz, CDCl_3_): *δ* 83.4, 76.0, 70.2, 63.2, 36.2, 32.8, 31.9, 29.5, 29.3, 29.1, 29.0, 28.9, 27.8, 26.0, 25.8, 25.5, 22.7, 18.7, 18.4, 14.1, −5.3; HRMS (ESI^+^): *m*/*z* calculated for C_24_H_48_NaO_2_Si^+^: 419.3316; [M + Na]^+^ found: 419.3316.

(*S*)-1-((*tert*-Butyldimethylsilyl)oxy)octadec-8-yn-6-ol (**26**). Colorless oil; yield 83%; [α]_D_^20^ = −2.0 (c 2.5, CH_2_Cl_2_).

### 2.5. General Procedure for the Synthesis of Alkenes from Alkynes Using Lindlar’s Catalyst

Lindlar’s catalyst (50 mg) was added to a round bottom flask containing alkyne **4a**,**b** (1.00 mmol) in MeOH (10 mL), followed by the addition of quinoline (6 μL, 0.05 mmol), and the reaction mixture was left stirring under a hydrogen atmosphere for 16 h. After filtration through a celite pad, the solvent was evaporated and the crude reaction mixture was purified by flash silica column chromatography eluting with petroleum ether (bp 40−60 °C):ethyl acetate (100:0–95:5) to give the desired hydroxy alkene.

(*S*,*Z*)-1-((*tert*-Butyldimethylsilyl)oxy)octadec-9-en-7-ol (**5a**). Colorless oil; yield 85%; [α]_D_^20^ = −2.0 (c 0.5, CH_2_Cl_2_); ^1^H NMR (200 MHz, CDCl_3_): *δ* 5.59–5.36 (2H, m, 2 × =CH), 3.64–3.51 (3H, m, CH_2_OTBDMS and OCH), 2.19 (2H, t, *J* = 6.6 Hz, CH_2_C=), 2.10–1.97 (2H, m, CH_2_C=), 1.64 (1H, s, OH), 1.54–1.21 (22H, m, 11 × CH_2_), 0.94–0.77 (12H, m, 4 × CH_3_), 0.03 (6H, s, 2 × CH_3_); ^13^C NMR (50 MHz, CDCl_3_): *δ* 133.5, 125.1, 71.4, 63.2, 36.8, 35.3, 32.8, 31.9, 29.7, 29.5, 29.3, 29.3, 27.4, 25.9, 25.8, 25.7, 22.6, 18.3, 14.1, −5.3; HRMS (ESI^+^): *m*/*z* calculated for C_24_H_50_NaO_2_Si^+^: 421.3472; [M + Na]^+^ found: 421.3473.

(*S*,*Z*)-1-((*tert*-Butyldimethylsilyl)oxy)hexadec-9-en-7-ol (**5b**). Colorless oil; yield 80%; [α]_D_^20^ = −1.0 (c 1.9, CH_2_Cl_2_); ^1^H NMR (200 MHz, CDCl_3_): *δ* 5.57–5.31 (2H, m, 2 × =CH), 3.65–3.46 (3H, m, CH_2_OTBDMS and OCH), 2.25–2.11 (2H, m, CH_2_C=), 2.11–1.96 (2H, m, CH_2_C=), 1.79 (1H, s, OH), 1.56–1.13 (18H, m, 9 × CH_2_), 0.99–0.64 (12H, m, 4 × CH_3_), 0.01 (6H, s, 2 × CH_3_); ^13^C NMR (50 MHz, CDCl_3_): *δ* 133.3, 125.1, 71.3, 63.2, 36.7, 35.3, 32.8, 31.7, 29.6, 29.4, 28.9, 27.4, 25.9, 25.7, 25.7, 22.6, 18.3, 14.0, −5.4; HRMS (ESI^+^): *m*/*z* calculated for C_22_H_46_NaO_2_Si^+^: 393.3159; [M + Na]^+^ found: 393.3152.

### 2.6. General Procedure for the Acetylation of Alcohols

Secondary alcohol **5a**,**b**, **12a**–**f**, **20** or **27** (1.00 mmol) in dry CH_2_Cl_2_ (10 mL) was added to a flame-dried flask under an argon atmosphere at 0 °C, followed by the addition of dry pyridine (0.12 mL, 1.50 mmol) and acetyl chloride (0.14 mL, 2.00 mmol). The reaction mixture was then left stirring for 16 h at room temperature. Then, a saturated aqueous solution of NH_4_Cl (10 mL) was added, and the aqueous layer was extracted with Et_2_O (3 × 20 mL), dried over Na_2_SO_4_, filtered, and concentrated in vacuo. The desired product was isolated by flash silica chromatography eluting with petroleum ether (bp 40−60 °C):ethyl acetate (90:10).

(*S*,*Z*)-1-((*tert*-Butyldimethylsilyl)oxy)octadec-9-en-7-yl acetate (**6a**). Colorless oil; yield 94%; [α]_D_^20^ = +2.0 (c 1.0, CH_2_Cl_2_); ^1^H NMR (200 MHz, CDCl_3_): *δ* 5.54–5.28 (2H, m, 2 × =CH), 4.94–4.80 (1H, m, OCH), 3.58 (2H, t, *J* = 6.3 Hz, CH_2_OTBDMS), 2.34–2.20 (2H, m, CH_2_C=), 2.09–1.92 (5H, m, CH_2_C= and COCH_3_), 1.58–1.11 (22H, m, 11 × CH_2_), 0.97–0.67 (12H, m, 4 × CH_3_), 0.03 (6H, s, 2 × CH_3_); ^13^C NMR (50 MHz, CDCl_3_): *δ* 170.8, 132.8, 124.1, 73.9, 63.2, 33.5, 32.7, 31.9, 29.6, 29.5, 29.3, 27.3, 26.0, 25.7, 25.4, 22.7, 21.2, 18.3, 14.1, −5.3; HRMS (ESI^+^): *m*/*z* calculated for C_26_H_52_NaO_3_Si^+^: 463.3578; [M + Na]^+^ found: 463.3578.

(*S*,*Z*)-1-((*tert*-Butyldimethylsilyl)oxy)hexadec-9-en-7-yl acetate (**6b**). Colorless oil; yield 96%; [α]_D_^20^ = +3.0 (c 2.0, CH_2_Cl_2_); ^1^H NMR (200 MHz, CDCl_3_): *δ* 5.55–5.26 (2H, m, 2 × =CH), 4.94–4.79 (1H, m, OCH), 3.58 (2H, t, *J* = 6.5 Hz, CH_2_OTBDMS), 2.37–2.19 (2H, m, CH_2_C=), 2.16–1.93 (5H, m, CH_2_C= and COCH_3_), 1.61–1.21 (18H, m, 9 × CH_2_), 1.03–0.82 (12H, m, 4 × CH_3_), 0.03 (6H, s, 2 × CH_3_); ^13^C NMR (50 MHz, CDCl_3_): *δ* 170.8, 132.7, 124.1, 73.9, 63.2, 33.5, 32.7, 31.9, 31.8, 29.5, 29.3, 29.0, 27.3, 25.9, 25.7, 25.4, 22.6, 21.2, 18.3, 14.1, −5.3; HRMS (ESI^+^): *m*/*z* calculated for C_24_H_48_NaO_3_Si^+^: 435.3265; [M + Na]^+^ found: 435.3261.

(*R*)-1-(Benzyloxy)hexadecan-6-yl acetate (*R*-**13a**). Colorless oil; yield 94%; [α]_D_^20^ = +2.5 (c 1.0, CHCl_3_); ^1^H NMR (400 MHz, CDCl_3_): *δ* 7.40–7.23 (5H, m, ArH), 4.93–4.79 (1H, m, OCH), 4.50 (2H, s, PhCH_2_O), 3.46 (2H, t, *J* = 6.5 Hz, OCH_2_), 2.03 (3H, s, COCH_3_), 1.65–1.57 (2H, m, CH_2_), 1.57–1.45 (4H, m, 2 × CH_2_), 1.45–1.17 (20H, m, 10 × CH_2_), 0.88 (3H, t, *J* = 6.8 Hz, CH_3_); ^13^C NMR (100 MHz, CDCl_3_): *δ* 170.9, 138.6, 128.3, 127.6, 127.5, 74.3, 72.9, 70.3, 34.1, 34.0, 31.9, 29.6, 29.6, 29.6, 29.5, 29.3, 26.1, 25.3, 25.2, 22.7, 21.3, 14.1; HRMS (ESI^+^): *m*/*z* calculated for C_25_H_42_NaO_3_^+^: 413.3026; [M + Na]^+^ found: 413.3043.

(*S*)-1-(Benzyloxy)hexadecan-6-yl acetate (*S*-**13a**). Colorless oil; yield 94%; [α]_D_^20^ = −2.0 (c 1.64, CHCl_3_).

(*R*)-1-(Benzyloxy)hexadecan-8-yl acetate (*R*-**13b**). Colorless oil; yield 89%; [α]_D_^20^ = +1.0 (c 1.0, CHCl_3_); ^1^H NMR (200 MHz, CDCl_3_): *δ* 7.44–7.19 (5H, m, ArH), 4.91–4.79 (1H, m, OCH), 4.50 (2H, s, PhCH_2_O), 3.46 (2H, t, *J* = 6.5 Hz, OCH_2_), 2.03 (3H, s, COCH_3_), 1.67–1.40 (6H, m, 3 × CH_2_), 1.40–1.02 (20H, m, 10 × CH_2_), 0.87 (3H, t, *J* = 6.2 Hz, CH_3_); ^13^C NMR (50 MHz, CDCl_3_): *δ* 171.0, 138.6, 128.3, 127.6, 127.4, 74.4, 72.8, 70.4, 34.1, 31.8, 29.7, 29.5, 29.5, 29.4, 29.2, 26.1, 25.3, 25.3, 22.6, 21.3, 14.1; HRMS (ESI^+^): *m*/*z* calculated for C_25_H_42_NaO_3_^+^: 413.3026; [M + Na]^+^ found: 413.3034.

(*S*)-1-(Benzyloxy)hexadecan-8-yl acetate (*S*-**13b**). Colorless oil; yield 95%; [α]_D_^20^ = −0.9 (c 1.54, CHCl_3_).

(*R*)-16-(Benzyloxy)hexadecan-6-yl acetate (*R*-**13c**). Colorless oil; yield 93%; [α]_D_^20^ = +1.3 (c 1.0, CH_2_Cl_2_); ^1^H NMR (400 MHz, CDCl_3_): *δ* 7.53–7.18 (5H, m, ArH), 4.92–4.80 (1H, m, OCH), 4.50 (2H, s, PhCH_2_O), 3.46 (2H, t, *J* = 6.7 Hz, OCH_2_), 2.04 (3H, s, COCH_3_), 1.65–1.57 (2H, m, CH_2_), 1.56–1.45 (4H, m, 2 × CH_2_), 1.45–1.11 (20H, m, 10 × CH_2_), 0.88 (3H, t, *J* = 6.8 Hz, CH_3_); ^13^C NMR (100 MHz, CDCl_3_): *δ* 171.0, 138.7, 128.3, 127.6, 127.4, 74.4, 72.8, 70.5, 34.1, 34.1, 31.7, 29.8, 29.5, 29.5, 29.5, 29.5, 26.2, 25.3, 25.0, 22.5, 21.3, 14.0; HRMS (ESI^+^): *m*/*z* calculated for C_25_H_42_NaO_3_^+^: 413.3026; [M + Na]^+^ found: 413.3034.

(*S*)-16-(Benzyloxy)hexadecan-6-yl acetate (*S*-**13c**). Colorless oil; yield 92%; [α]_D_^20^ = −0.9 (c 1.0, CH_2_Cl_2_).

(*R*)-1-(Benzyloxy)heptadecan-7-yl acetate (*R*-**13d**). Colorless oil; Yield 94%; [α]_D_^20^ = +1.7 (c 0.83, CH_2_Cl_2_); ^1^H NMR (400 MHz, CDCl_3_): *δ* 7.47–7.18 (5H, m, ArH), 4.93–4.79 (1H, m, OCH), 4.50 (2H, s, PhCH_2_O), 3.46 (2H, t, *J* = 6.5 Hz, OCH_2_), 2.03 (3H, s, COCH_3_), 1.65–1.48 (6H, m, 3 × CH_2_), 1.40–1.21 (22H, m, 11 × CH_2_), 0.88 (3H, t, *J* = 6.2 Hz, CH_3_); ^13^C NMR (100 MHz, CDCl_3_): *δ* 170.9, 138.7, 128.3, 127.6, 127.4, 74.4, 72.9, 70.4, 34.1, 34.0, 31.9, 29.7, 29.6, 29.6, 29.5, 29.3, 29.3, 26.1, 25.3, 25.2, 22.7, 21.2, 14.1; HRMS (ESI^+^): *m*/*z* calculated for C_26_H_44_NaO_3_^+^: 427.3183; [M + Na]^+^ found: 427.3192.

(*S*)-1-(Benzyloxy)heptadecan-7-yl acetate (*S*-**13d**). Colorless oil; yield 92%; [α]_D_^20^ = −1.3 (c 1.0, CH_2_Cl_2_).

(*R*)-1-(Benzyloxy)octadecan-8-yl acetate (*R*-**13e**). Colorless oil; yield 97%; [α]_D_^20^ = +1.2 (c 0.8, CH_2_Cl_2_); ^1^H NMR (400 MHz, CDCl_3_): *δ* 7.58–7.13 (5H, m, ArH), 4.96–4.76 (1H, m, OCH), 4.50 (2H, s, PhCH_2_O), 3.46 (2H, t, *J* = 6.6 Hz, OCH_2_), 2.03 (3H, s, COCH_3_), 1.74–1.40 (8H, m, 4 × CH_2_), 1.38–1.23 (22H, m, 11 × CH_2_), 0.88 (3H, t, *J* = 6.5 Hz, CH_3_); ^13^C NMR (100 MHz, CDCl_3_): *δ* 170.9, 138.7, 128.3, 127.6, 127.4, 74.4, 72.8, 70.5, 34.1, 34.1, 31.9, 29.7, 29.6, 29.6, 29.5, 29.5, 29.3, 29.3, 26.1, 25.3, 25.2, 22.7, 21.3, 14.1; HRMS (ESI^+^): *m*/*z* calculated for C_27_H_46_NaO_3_^+^: 441.3339; [M + Na]^+^ found: 441.3347.

(*S*)-1-(Benzyloxy)octadecan-8-yl acetate (*S*-**13e**). Colorless oil; yield 99%; [α]_D_^20^ = −1.9 (c 1.6, CH_2_Cl_2_).

(*R*)-18-(Benzyloxy)octadecan-8-yl acetate (*R*-**13f**). Colorless oil; yield 94%; [α]_D_^20^ = +1.0 (c 0.5, CH_2_Cl_2_); ^1^H NMR (400 MHz, CDCl_3_): *δ* 7.44–7.18 (5H, m, ArH), 4.94–4.78 (1H, m, OCH), 4.50 (2H, s, PhCH_2_O), 3.46 (2H, t, *J* = 6.6 Hz, OCH_2_), 2.04 (3H, s, COCH_3_), 1.65–1.57 (2H, m, CH_2_), 1.56–1.44 (4H, m, 2 × CH_2_), 1.43–1.16 (24H, m, 12 × CH_2_), 0.88 (3H, t, *J* = 6.8 Hz, CH_3_); ^13^C NMR (100 MHz, CDCl_3_): *δ* 170.9, 138.7, 128.3, 127.6, 127.4, 74.5, 72.8, 70.5, 34.1, 31.8, 29.8, 29.6, 29.5, 29.5, 29.5, 29.2, 26.2, 25.3, 22.6, 21.3, 14.1; HRMS (ESI^+^): *m*/*z* calculated for C_27_H_46_NaO_3_^+^: 441.3339; [M + Na]^+^ found: 441.3339.

(*S*)-18-(Benzyloxy)octadecan-8-yl acetate (*S*-**13f**). Colorless oil; yield 95%; [α]_D_^20^ = −2.0 (c 1.0, CH_2_Cl_2_).

(*S*)-1-((*tert*-Butyldimethylsilyl)oxy)octadecan-6-yl acetate (**21**). Colorless oil; yield 92%; [α]_D_^20^ = +0.8 (c 0.5, CH_2_Cl_2_); ^1^H NMR (400 MHz, CDCl_3_): *δ* 4.91–4.79 (1H, m, OCH), 3.59 (2H, t, *J* = 6.5 Hz, CH_2_OTBDMS), 2.03 (3H, s, COCH_3_), 1.55–1.46 (6H, m, 3 × CH_2_), 1.36–1.22 (24H, m, 12 × CH_2_), 0.93–0.84 (12H, m, 4 × CH_3_), 0.04 (6H, s, 2 × CH_3_); ^13^C NMR (100 MHz, CDCl_3_): *δ* 170.9, 74.4, 63.1, 34.1, 32.7, 31.9, 29.7, 29.6, 29.6, 29.5, 29.3, 26.0, 25.8, 25.3, 25.1, 22.7, 21.3, 18.4, 14.1, −5.3; HRMS (ESI^+^): *m*/*z* calculated for C_26_H_54_NaO_3_Si^+^: 465.3734; [M + Na]^+^ found: 465.3735.

(*R*)-1-((*tert*-Butyldimethylsilyl)oxy)octadecan-6-yl acetate (**28**). Colorless oil; yield 95%; [α]_D_^20^ = −2.0 (c 1.4, CH_2_Cl_2_).

### 2.7. General Procedure for the Deprotection of the Tert-Butyldimethylsilyl (TBDMS) Group

To a flame-dried flask under an argon atmosphere, the appropriate TBDMS-protected alcohol (1.00 mmol) in dry THF (5 mL) was added and, then, a solution of tetra-*N*-butylammonium fluoride (1M in THF, 1 mL, 1.00 mmol) was added dropwise at 0 °C. The reaction mixture was then left to reach room temperature, and stirring was continued for 1 h. The solvent was removed under reduced pressure and the crude reaction mixture was purified by flash chromatography on silica gel eluting with petroleum ether (bp 40−60 °C):ethyl acetate (80:20–70:30) to give the desired product.

(*S*,*Z*)-1-Hydroxyoctadec-9-en-7-yl acetate (**7a**). Colorless oil; yield 82%; [α]_D_^20^ = −8.0 (c 2.0, CH_2_Cl_2_); ^1^H NMR (200 MHz, CDCl_3_): *δ* 5.52–5.25 (2H, m, 2 × =CH), 4.94–4.78 (1H, m, OCH), 3.68–3.58 (3H, m, C*H*_2_OH and OH), 2.38–2.24 (2H, m, CH_2_C=), 2.08–1.92 (5H, m, CH_2_C= and COCH_3_), 1.71–1.41 (6H, m, 3 × CH_2_), 1.40–1.17 (16H, m, 8 × CH_2_), 0.86 (3H, t, *J* = 6.3 Hz, CH_3_); ^13^C NMR (50 MHz, CDCl_3_): *δ* 170.8, 132.8, 124.0, 73.9, 62.8, 33.5, 33.0, 32.6, 31.9, 31.8, 29.5, 29.5, 29.3, 29.2, 27.3, 25.6, 25.3, 22.6, 21.2, 14.1; HRMS (ESI^+^): *m*/*z* calculated for C_20_H_38_NaO_3_^+^: 349.2713; [M + Na]^+^ found: 349.2713.

(*S*,*Z*)-1-Hydroxyhexadec-9-en-7-yl acetate (**7b**). Colorless oil; yield 76%; [α]_D_^20^ = −9.6 (c 2.5, CH_2_Cl_2_); ^1^H NMR (400 MHz, CDCl_3_): *δ* 5.55–5.39 (1H, m, =CH), 5.38–5.23 (1H, m, =CH), 4.98–4.71 (1H, m, OCH), 3.62 (2H, t, *J* = 6.1 Hz, C*H*_2_OH), 2.36–2.16 (2H, m, CH_2_C=), 2.12–1.91 (5H, m, CH_2_C= and COCH_3_), 1.61–1.49 (5H, m, 2 × CH_2_ and OH), 1.40–1.20 (14H, m, 7 × CH_2_), 0.87 (3H, t, *J* = 6.0 Hz, CH_3_); ^13^C NMR (100 MHz, CDCl_3_): *δ* 170.9, 132.8, 124.0, 73.9, 62.9, 33.5, 32.6, 31.9, 31.7, 29.5, 29.2, 29.0, 27.3, 25.6, 25.3, 22.6, 21.2, 14.1; HRMS (ESI^+^): *m*/*z* calculated for C_18_H_34_NaO_3_^+^: 321.2400; [M + Na]^+^ found: 321.2404.

(*S*)-1-Hydroxyoctadecan-6-yl acetate (**22**). Colorless oil; yield 89%; [α]_D_^20^ = +1.3 (c 0.5, CH_2_Cl_2_); ^1^H NMR (400 MHz, CDCl_3_): *δ* 4.91–4.82 (1H, m, OCH), 3.63 (2H, t, *J* = 6.5 Hz, C*H*_2_OH), 2.04 (3H, s, COCH_3_), 1.60–1.48 (6H, m, 3 × CH_2_), 1.43–1.18 (25H, m, 12 × CH_2_ and OH), 0.88 (3H, t, *J* = 6.8 Hz, CH_3_); ^13^C NMR (100 MHz, CDCl_3_): *δ* 171.0, 74.3, 62.8, 34.2, 34.1, 32.6, 31.9, 29.7, 29.6, 29.6, 29.5, 29.3, 25.6, 25.3, 25.0, 22.7, 21.3, 14.1; HRMS (ESI^+^): *m*/*z* calculated for C_20_H_40_NaO_3_^+^: 351.2870; [M + Na]^+^ found: 351.2869.

(*R*)-Hydroxyoctadecan-6-yl acetate (**29**). Colorless oil; yield 92%; [α]_D_^20^ = −1.8 (c 0.5, CH_2_Cl_2_).

### 2.8. General Procedure for the Oxidation of Alcohols to Acids Using Jones Reagent

To a round-bottomed flask containing an alcohol (1.00 mmol) in acetone (10 mL), the Jones reagent (2 M, 1.5 mL, 3.00 mmol) was added dropwise at 0 °C and the reaction mixture was left under stirring at this temperature for 1 h. Then, the reaction mixture was quenched with a saturated solution of NaHSO_3_ (10 mL), which was added at room temperature. The aqueous layer was extracted with Et_2_O (3 × 20 mL), washed with brine (1 × 50 mL), dried over Na_2_SO_4_, filtered, and concentrated under reduced pressure to give a crude mixture, which was then purified by flash chromatography eluting with petroleum ether (bp 40−60 °C):ethyl acetate (60:40) to afford the desired acid.

(*S*,*Z*)-7-Acetoxyoctadec-9-enoic acid (**8a**). Colorless oil; yield 80%; [α]_D_^20^ = −1.5 (c 1.0, CH_2_Cl_2_); ^1^H NMR (200 MHz, CDCl_3_): *δ* 10.14 (1H, br s, COOH), 5.52–5.21 (2H, m, 2 × =CH), 4.91–4.77 (1H, m, OCH), 2.41–2.19 (4H, m, CH_2_C= and C*H*_2_COOH), 2.09–1.84 (5H, m, CH_2_C= and COCH_3_), 1.73–1.05 (20H, m, 10 × CH_2_), 0.85 (3H, t, *J* = 6.1 Hz, CH_3_); ^13^C NMR (50 MHz, CDCl_3_): *δ* 179.8, 170.9, 132.8, 123.9, 73.8, 33.9, 33.3, 31.9, 31.8, 29.5, 29.5, 29.3, 28.8, 27.3, 25.0, 24.5, 22.6, 21.2, 14.1; HRMS (ESI^−^): *m*/*z* calculated for C_20_H_35_O_4_^−^: 339.2541; [M − H]^−^ found: 339.2540.

(*S*,*Z*)-7-Acetoxyhexadec-9-enoic acid (**8b**). Colorless oil; yield 85%; [α]_D_^20^ = −3.0 (c 1.43, CH_2_Cl_2_); ^1^H NMR (200 MHz, CDCl_3_): *δ* 9.69 (1H, br s, COOH), 5.53–5.22 (2H, m, 2 × =CH), 4.93–4.79 (1H, m, OCH), 2.40–2.15 (4H, m, CH_2_C= and C*H*_2_COOH), 2.13–1.84 (5H, m, CH_2_C= and COCH_3_), 1.74–1.10 (16H, m, 8 × CH_2_), 0.86 (3H, t, *J* = 6.3 Hz, CH_3_); ^13^C NMR (50 MHz, CDCl_3_): *δ* 179.9, 170.9, 132.8, 123.9, 73.8, 33.9, 33.3, 31.9, 31.7, 29.5, 28.9, 28.8, 27.3, 25.0, 24.5, 22.6, 21.2, 14.1; HRMS (ESI^−^): *m*/*z* calculated for C_18_H_31_O_4_^−^: 311.2228; [M − H]^−^ found: 311.2226.

(*R*)-6-Acetoxyhexadecanoic acid (*R*-**15a**). White solid; yield 84%; m.p.: 39–41 °C; [α]_D_^20^ = +2.8 (c 1.43, CH_2_Cl_2_); ^1^H NMR (400 MHz, CDCl_3_): *δ* 4.94–4.80 (1H, m, OCH), 2.34 (2H, t, *J* = 7.4 Hz, C*H*_2_COOH), 2.03 (3H, s, COCH_3_), 1.71–1.47 (6H, m, 3 × CH_2_), 1.41–1.21 (18H, m, 9 × CH_2_), 0.87 (3H, t, *J* = 6.8 Hz, CH_3_); ^13^C NMR (100 MHz, CDCl_3_): *δ* 179.5, 171.0, 74.1, 34.1, 33.8, 33.7, 31.9, 29.6, 29.5, 29.5, 29.3, 25.3, 24.8, 24.5, 22.7, 21.2, 14.1; HRMS (ESI^−^): *m*/*z* calculated for C_18_H_33_O_4_^−^: 313.2384; [M − H]^−^ found: 313.2378.

(*S*)-6-Acetoxyhexadecanoic acid (*S*-**15a**). White solid; yield 85%; m.p.: 39–41 °C; [α]_D_^20^ = −2.0 (c 1.0, CH_2_Cl_2_).

(*R*)-8-Acetoxyhexadecanoic acid (*R*-**15b**). Colorless oil; yield 87%; [α]_D_^20^ = −0.8 (c 0.5, CH_3_OH); ^1^H NMR (200 MHz, CDCl_3_): *δ* 4.90–4.78 (1H, m, OCH), 2.34 (2H, t, *J* = 7.4 Hz, C*H*_2_COOH), 2.03 (3H, s, COCH_3_), 1.74–1.44 (6H, m, 3 × CH_2_), 1.44–1.09 (18H, m, 9 × CH_2_), 0.87 (3H, t, *J* = 6.4 Hz, CH_3_); ^13^C NMR (50 MHz, CDCl_3_): *δ* 179.8, 171.0, 74.4, 34.1, 34.0, 33.9, 31.8, 29.5, 29.5, 29.2, 29.1, 28.9, 25.3, 25.1, 24.5, 22.6, 21.3, 14.1; HRMS (ESI^−^): *m*/*z* calculated for C_18_H_33_O_4_^−^: 313.2384; [M − H]^−^ found: 313.2376.

(*S*)-8-Acetoxyhexadecanoic acid (*S*-**15b**) [21]. Colorless oil; yield 88%; [α]_D_^20^ = +0.6 (c 0.5, CH_3_OH), [α]_D_^22^ lit. = +0.48 (c 2.8, CH_3_OH).

(*R*)-11-Acetoxyhexadecanoic acid (*R*-**15c**). Colorless oil; yield 87%; [α]_D_^20^ = +0.8 (c 1.0, CHCl_3_); ^1^H NMR (400 MHz, CDCl_3_): *δ* 4.92–4.79 (1H, m, OCH), 2.34 (2H, t, *J* = 7.5 Hz, C*H*_2_COOH), 2.04 (3H, s, COCH_3_), 1.67–1.59 (2H, m, CH_2_), 1.55–1.46 (4H, m, 2 × CH_2_), 1.36–1.22 (18 H, m, 9 × CH_2_), 0.88 (3H, t, *J* = 6.8 Hz, CH_3_); ^13^C NMR (100 MHz, CDCl_3_): *δ* 179.1, 171.0, 74.5, 34.1, 34.1, 33.9, 31.7, 29.5, 29.4, 29.3, 29.2, 29.0, 25.3, 25.0, 24.7, 22.5, 21.3, 14.0; HRMS (ESI^−^): *m*/*z* calculated for C_18_H_33_O_4_^−^: 313.2384; [M − H]^−^ found: 313.2381.

(*S*)-11-Acetoxyhexadecanoic acid (*S*-**15c**) [22]. Colorless oil; yield 84%; [α]_D_^20^ = −1.0 (c 1.0, CHCl_3_), [α]_D_^23^ lit. = −0.79 (c 14.85, CHCl_3_).

(*R*)-7-Acetoxyheptadecanoic acid (*R*-**15d**). Colorless oil; yield 88%; [α]_D_^20^ = +0.8 (c 0.7, CH_2_Cl_2_); ^1^H NMR (400 MHz, CDCl_3_): *δ* 4.93–4.79 (1H, m, OCH), 2.35 (2H, t, *J* = 7.4 Hz, C*H*_2_COOH), 2.04 (3H, s, COCH_3_), 1.68–1.60 (2H, m, CH_2_), 1.58–1.45 (4H, m, 2 × CH_2_), 1.41–1.18 (20H, m, 10 × CH_2_), 0.88 (3H, t, *J* = 6.6 Hz, CH_3_); ^13^C NMR (100 MHz, CDCl_3_): *δ* 179.1, 171.0, 74.3, 34.1, 33.9, 33.8, 31.9, 29.6, 29.6, 29.5, 29.3, 28.9, 25.3, 24.9, 24.5, 22.7, 21.3, 14.1; HRMS (ESI^−^): *m*/*z* calculated for C_19_H_35_O_4_^−^: 327.2541; [M − H]^−^ found: 327.2533.

(*S*)-7-Acetoxyheptadecanoic acid (*S*-**15d**). Colorless oil; yield 92%; [α]_D_^20^ = −1.4 (c 1.4, CH_2_Cl_2_).

(*R*)-8-Acetoxyoctadecanoic acid (*R*-**15e**) [23]. White solid; yield 80%; m.p.: 42–43 °C; [α]_D_^20^ = +1.8 (c 1.3, CH_2_Cl_2_); ^1^H NMR (400 MHz, CDCl_3_): *δ* 4.93–4.78 (1H, m, OCH), 2.34 (2H, t, *J* = 7.5 Hz, C*H*_2_COOH), 2.03 (3H, s, COCH_3_), 1.67–1.47 (6H, m, 3 × CH_2_), 1.40–1.19 (22H, m, 11 × CH_2_), 0.88 (3H, t, *J* = 6.6 Hz, CH_3_); ^13^C NMR (100 MHz, CDCl_3_): *δ* 179.4, 171.0, 74.4, 34.1, 34.0, 33.9, 31.9, 29.6, 29.6, 29.5, 29.3, 29.1, 28.9, 25.3, 25.1, 24.6, 22.7, 21.3, 14.1; HRMS (ESI^−^): *m*/*z* calculated for C_20_H_37_O_4_^−^: 341.2697; [M − H]^−^ found: 341.2697.

(*S*)-8-Acetoxyoctadecanoic acid (*S*-**15e**). White solid; yield 82%; m.p.: 42–43 °C; [α]_D_^20^ = −2.0 (c 1.0, CH_2_Cl_2_).

(*R*)-11-Acetoxyoctadecanoic acid (*R*-**15f**). Colorless oil; yield 86%; [α]_D_^20^ = +0.8 (c 0.7, CH_2_Cl_2_); ^1^H NMR (400 MHz, CDCl_3_): *δ* 11.01 (1H, br s, COOH), 5.00–4.69 (1H, m, OCH), 2.34 (2H, t, *J* = 7.4 Hz, C*H*_2_COOH), 2.03 (3H, s, COCH_3_), 1.67–1.47 (6H, m, 3 × CH_2_), 1.41–1.20 (22H, m, 11 × CH_2_), 0.87 (3H, t, *J* = 6.0 Hz, CH_3_); ^13^C NMR (100 MHz, CDCl_3_): *δ* 179.5, 171.0, 74.5, 34.1, 33.9, 31.8, 29.5, 29.5, 29.4, 29.3, 29.2, 29.2, 29.0, 25.3, 24.6, 22.6, 21.3, 14.1; HRMS (ESI^−^): *m*/*z* calculated for C_20_H_37_O_4_^−^: 341.2697; [M − H]^−^ found: 341.2697.

(*S*)-11-Acetoxyoctadecanoic acid (*S*-**15f**). Colorless oil; yield 84%; [α]_D_^20^ = −1.0 (c 0.5, CH_2_Cl_2_).

(*S*)-6-Acetoxyoctadecanoic acid (**23**). White solid; yield 80%; m.p.: 47–49 °C; [α]_D_^20^ = −1.0 (c 0.5, CH_2_Cl_2_); ^1^H NMR (400 MHz, CDCl_3_): *δ* 4.92–4.78 (1H, m, OCH), 2.35 (2H, t, *J* = 7.4 Hz, C*H*_2_COOH), 2.03 (3H, s, COCH_3_), 1.68–1.49 (6H, m, 3 × CH_2_), 1.40–1.22 (22H, m, 11 × CH_2_), 0.88 (3H, t, *J* = 6.8 Hz, CH_3_); ^13^C NMR (100 MHz, CDCl_3_): *δ* 179.1, 171.0, 74.1, 34.1, 33.8, 33.7, 31.9, 29.7, 29.6, 29.6, 29.5, 29.5, 29.3, 25.3, 24.8, 24.5, 22.7, 21.2, 14.1; HRMS (ESI^−^): *m*/*z* calculated for C_20_H_37_O_4_^−^: 341.2697; [M − H]^−^ found: 341.2692.

(*R*)-6-Acetoxyoctadecanoic acid (**30**). White solid; yield 81%; m.p.: 47–49 °C; [α]_D_^20^ = +1.2 (c 0.8, CH_2_Cl_2_).

### 2.9. General Procedure for the Removal of the Acetyl Group

LiOH·H_2_O (168 mg, 4.00 mmol) was added to a solution containing acid **8a**,**b**, **15a**–**f**, **23**, or **30** (1.00 mmol) in THF:H_2_O (1:1, 5 mL). The resulting reaction mixture was stirred at room temperature for 16 h. Subsequently, the pH of the reaction mixture was adjusted to one by addition of an aqueous solution of HCl 1 N (10 mL). The aqueous layer was then subjected to extraction with EtOAc (3 × 10 mL), and the combined organic layers were washed with brine (1 × 30 mL), dried over Na_2_SO_4_, filtered, and concentrated under reduced pressure. Lastly, the isolation of the desired hydroxy fatty acids followed, using silica gel flash chromatography eluting with petroleum ether (bp 40−60 °C):ethyl acetate (20:80).

(*S*,*Z*)-7-Hydroxyoctadec-9-enoic acid (**9a**). Colorless oil; yield 77%; [α]_D_^20^ = −2.0 (c 1.0, CH_2_Cl_2_); ^1^H NMR (400 MHz, CDCl_3_): *δ* 5.62–5.52 (1H, m, =CH), 5.44–5.35 (1H, m, =CH), 3.65–3.57 (1H, m, OCH), 2.36 (2H, t, *J* = 7.5 Hz, C*H*_2_COOH), 2.21 (2H, t, *J* = 6.3 Hz, CH_2_C=), 2.09–2.01 (2H, m, CH_2_C=), 1.70–1.61 (2H, m, CH_2_), 1.56–1.19 (19H, m, 9 × CH_2_ and OH), 0.88 (3H, t, *J* = 6.9 Hz, CH_3_); ^13^C NMR (100 MHz, CDCl_3_): *δ* 179.0, 133.7, 124.9, 71.4, 36.5, 35.4, 33.8, 31.9, 29.7, 29.5, 29.3, 29.3, 29.1, 27.4, 25.4, 24.6, 22.7, 14.1; HRMS (ESI^−^): *m*/*z* calculated for C_18_H_33_O_3_^−^: 297.2435; [M − H]^−^ found: 297.2432.

(*S*,*Z*)-7-Hydroxyhexadec-9-enoic acid (**9b**). Colorless oil; yield 74%; [α]_D_^20^ = −1.5 (c 2.0, CH_2_Cl_2_); ^1^H NMR (400 MHz, CDCl_3_): *δ* 5.63–5.52 (1H, m, =CH), 5.46–5.34 (1H, m, =CH), 3.72–3.48 (1H, m, OCH), 2.35 (2H, t, *J* = 7.5 Hz, C*H*_2_COOH), 2.21 (2H, t, *J* = 6.8 Hz, CH_2_C=), 2.10–1.96 (2H, m, CH_2_C=), 1.70–1.59 (2H, m, CH_2_), 1.53–1.20 (15H, m, 7 × CH_2_ and OH), 0.88 (3H, t, *J* = 6.4 Hz, CH_3_); ^13^C NMR (100 MHz, CDCl_3_): *δ* 179.0, 133.7, 124.9, 71.4, 36.5, 35.4, 33.8, 31.7, 29.7, 29.6, 29.0, 29.0, 27.4, 25.4, 24.6, 22.6, 14.1; HRMS (ESI^−^): *m*/*z* calculated for C_16_H_29_O_3_^−^: 269.2122; [M − H]^−^ found: 269.2116.

(*R*)-6-Hydroxyhexadecanoic acid (*R*-**16a**). White solid; yield 75%; m.p.: 77–80 °C, [α]_D_^20^ = −1.4 (c 1.0, CH_2_Cl_2_); ^1^H NMR (400 MHz, CDCl_3_): *δ* 5.76 (1H, br s, COOH), 3.66–3.54 (1H, m, OCH), 2.35 (2H, t, *J* = 7.4 Hz, C*H*_2_COOH), 1.73–1.55 (2H, m, CH_2_), 1.54–1.08 (23H, m, 11 × CH_2_ and OH), 0.87 (3H, t, *J* = 6.8 Hz, CH_3_); ^13^C NMR (100 MHz, CDCl_3_): *δ* 179.1, 71.8, 37.4, 36.8, 33.9, 31.9, 29.7, 29.6, 29.3, 25.6, 25.1, 24.6, 22.7, 14.1; HRMS (ESI^−^): *m*/*z* calculated for C_16_H_31_O_3_^−^: 271.2279; [M − H]^−^ found: 271.2278.

(*S*)-6-Hydroxyhexadecanoic acid (*S*-**16a**). White solid; yield 74%, m.p.: 77–80 °C; [α]_D_^20^ = +2.0 (c 1.0, CH_2_Cl_2_).

(*R*)-8-Hydroxyhexadecanoic acid (*R*-**16b**) [24]. White solid; yield 71%; m.p.: 75–78 °C (lit. m.p.: 78.5–79 °C); [α]_D_^20^ = −0.8 (c 0.8, CHCl_3_), [α]_D_^20^.^5^ lit. = −0.51 (c 2.73, CHCl_3_); ^1^H NMR (400 MHz, CDCl_3_): *δ* 6.20 (1H, br s, COOH), 3.65–3.53 (1H, m, OCH), 2.32 (2H, t, *J* = 7.4 Hz, C*H*_2_COOH), 1.67–1.57 (2H, m, CH_2_), 1.54–1.11 (23H, m, 11 × CH_2_ and OH), 0.87 (3H, t, *J* = 6.4 Hz, CH_3_); ^13^C NMR (100 MHz, CDCl_3_): *δ* 179.2, 72.1, 37.3, 37.2, 34.0, 31.8, 29.7, 29.5, 29.2, 29.0, 25.6, 25.3, 24.6, 22.6, 14.0; HRMS (ESI^−^): *m*/*z* calculated for C_16_H_31_O_3_^−^: 271.2279; [M − H]^−^ found: 271.2275.

(*S*)-8-Hydroxyhexadecanoic acid (*S*-**16b**) [21]. White solid; yield 69%; m.p.: 75–78 °C (lit. m.p.: 77–79.5 °C); [α]_D_^20^ = +0.9 (c 0.5, CHCl_3_), [α]_D_^22^ lit. = +1.06 (c 2.19, CHCl_3_).

(*R*)-11-Hydroxyhexadecanoic acid (*R*-**16c**) [25]. White solid; yield 74%; m.p.: 63–66 °C (lit. m.p.: 65–67 °C); [α]_D_^20^ = −1.0 (c 1.0, CHCl_3_), [α]_D_^24^ lit. = −0.8 (c 0.8, CHCl_3_); ^1^H NMR (400 MHz, CDCl_3_): *δ* 3.64–3.55 (1H, m, OCH), 2.34 (2H, t, *J* = 7.4 Hz, C*H*_2_COOH), 1.72–1.52 (2H, m, CH_2_), 1.50–1.19 (23H, m, 11 × CH_2_ and OH), 0.89 (3H, t, *J* = 6.1 Hz, CH_3_); ^13^C NMR (100 MHz, CDCl_3_): *δ* 179.0, 72.1, 37.4, 33.9, 31.9, 29.6, 29.5, 29.3, 29.1, 29.0, 25.6, 25.3, 24.6, 22.6, 14.0; HRMS (ESI^−^): *m*/*z* calculated for C_16_H_31_O_3_^−^: 271.2279; [M − H]^−^ found: 271.2279.

(*S*)-11-Hydroxyhexadecanoic acid (*S*-**16c**) [25]. White solid; yield 73%; m.p.: 63–66 °C (lit. m.p.: 65–67 °C); [α]_D_^20^ = +1.0 (c 1.0, CHCl_3_), [α]_D_^24^ lit. = +0.9 (c 0.7, CHCl_3_).

(*R*)-7-Hydroxyheptadecanoic acid (*R*-**16d**). White solid; yield 77%; m.p.: 68–71 °C; [α]_D_^20^ = −1.0 (c 1.0, CH_2_Cl_2_); ^1^H NMR (400 MHz, CDCl_3_): *δ* 5.29 (1H, br s, COOH), 3.64–3.54 (1H, m, OCH), 2.34 (2H, t, *J* = 7.4 Hz, C*H*_2_COOH), 1.70–1.59 (2H, m, CH_2_), 1.56–1.08 (25H, m, 12 × CH_2_ and OH), 0.88 (3H, t, *J* = 6.4 Hz, CH_3_); ^13^C NMR (100 MHz, CDCl_3_): *δ* 179.1, 72.0, 37.5, 37.1, 33.9, 31.9, 29.7, 29.6, 29.3, 29.1, 25.6, 25.2, 24.6, 22.7, 14.1; HRMS (ESI^−^): *m*/*z* calculated for C_17_H_33_O_3_^−^: 285.2435; [M − H]^−^ found: 285.2430.

(*S*)-7-Hydroxyheptadecanoic acid (*S*-**16d**). White solid; yield 67%; m.p.: 68–71 °C; [α]_D_^20^ = +1.4 (c 1.0, CH_2_Cl_2_).

(*R*)-8-Hydroxyoctadecanoic acid (*R*-**16e**) [26]. White solid; yield 70%; m.p.: 77–79 °C (lit. m.p.: 77.5–78 °C); [α]_D_^20^ = −1.5 (c 0.76, CH_2_Cl_2_), [α]_D_^20^ lit. = −1.6 (c 6.0, CHCl_3_); ^1^H NMR (400 MHz, CDCl_3_): *δ* 5.76 (1H, br s, COOH), 3.74–3.46 (1H, m, OCH), 2.33 (2H, t, *J* = 7.4 Hz, C*H*_2_COOH), 1.80–1.49 (3H, m, CH_2_ and OH), 1.49–1.19 (26H, m, 13 × CH_2_), 0.87 (3H, t, *J* = 6.5 Hz, CH_3_); ^13^C NMR (100 MHz, CDCl_3_): *δ* 179.3, 72.1, 37.4, 37.3, 34.0, 31.9, 29.7, 29.6, 29.3, 29.3, 29.0, 25.6, 25.4, 24.6, 22.7, 14.1; HRMS (ESI^−^): *m*/*z* calculated for C_18_H_35_O_3_^−^: 299.2592; [M − H]^−^ found: 299.2586.

(*S*)-8-Hydroxyoctadecanoic acid (*S*-**16e**). White solid; yield 68%; m.p.: 77–79 °C; [α]_D_^20^ = +2.5 (c 1.0, CH_2_Cl_2_).

(*R*)-11-Hydroxyoctadecanoic acid (*R*-**16f**). White solid; yield 77%; m.p.: 75–77 °C (lit. m.p._rac_. [27]: 77.2–77.5 °C); [α]_D_^20^ = −3.3 (c 0.75, CH_2_Cl_2_); ^1^H NMR (400 MHz, CDCl_3_): *δ* 3.63–3.54 (1H, m, OCH), 2.34 (2H, t, *J* = 7.3 Hz, C*H*_2_COOH), 1.69–1.59 (2H, m, CH_2_), 1.55–1.09 (27H, m, 13 × CH_2_ and OH), 0.88 (3H, t, *J* = 6.3 Hz, CH_3_); ^13^C NMR (100 MHz, CDCl_3_): *δ* 179.0, 72.1, 37.4, 37.4, 33.9, 31.8, 29.7, 29.6, 29.5, 29.3, 29.1, 29.0, 25.6, 25.6, 24.7, 22.6, 14.1; HRMS (ESI^−^): *m*/*z* calculated for C_18_H_35_O_3_^−^: 299.2592; [M − H]^−^ found: 299.2592.

(*S*)-11-Hydroxyoctadecanoic acid (*S*-**16f**). White solid; yield 76%; m.p.: 75–77 °C (lit. m.p._rac_. [27]: 77.2–77.5 °C); [α]_D_^20^ = +3.6 (c 0.5, CH_2_Cl_2_).

(*S*)-6-Hydroxyoctadecanoic acid (**24**). White solid; yield 67%; m.p.: 80–81 °C (lit. m.p._rac_. [27]: 82–82.4 °C); [α]_D_^20^ = +3.0 (c 0.77, CH_2_Cl_2_); ^1^H NMR (400 MHz, CDCl_3_): *δ* 3.64–3.56 (1H, m, OCH), 2.37 (2H, t, *J* = 7.4 Hz, C*H*_2_COOH), 1.73–1.60 (2H, m, CH_2_), 1.58–1.02 (27H, m, 13 × CH_2_ and OH), 0.88 (3H, t, *J* = 6.7 Hz, CH_3_); ^13^C NMR (100 MHz, CDCl_3_): *δ* 179.1, 71.8, 37.5, 36.9, 33.9, 31.9, 29.7, 29.7, 29.6, 29.6, 29.3, 25.6, 25.1, 24.6, 22.7, 14.1; HRMS (ESI^−^): *m*/*z* calculated for C_18_H_35_O_3_^−^: 299.2592; [M − H]^−^ found: 299.2592.

(*R*)-6-Hydroxyoctadecanoic acid (**31**). White solid; yield 69%; m.p.: 80–81 °C (lit. m.p._rac_. [27]: 82–82.4 °C); [α]_D_^20^ = −2.5 (c 1.0, CH_2_Cl_2_).

### 2.10. General Procedure for the Synthesis of Secondary Alcohols Using Grignard Reagents

Nonylmagnesium bromide (or heptylmagnesium bromide or butylmagnesium chloride or hexylmagnesium bromide) (2 M solution in diethyl ether, 1 mL, 2.00 mmol) was introduced to a flame-dried flask containing copper(I) iodide (38 mg, 0.20 mmol) under an argon atmosphere. After cooling the reaction mixture at −40 °C and stirring it for 10 min, the appropriate epoxide (1.00 mmol) in dry THF (10 mL) was added dropwise. Stirred at −40 °C for 1 h, the reaction mixture was then brought to room temperature. Subsequently, 10 mL of a saturated aqueous NH_4_Cl solution were added, and the resulting aqueous layer underwent extraction with Et_2_O (3 × 20 mL). The combined organic layers were washed with brine (1 × 50 mL), dried over Na_2_SO_4_, filtered, and then concentrated under reduced pressure. The desired alcohol was isolated through silica gel flash chromatography using petroleum ether (bp 40−60 °C):ethyl acetate (80:20–70:30) as the elution system.

(*R*)-1-(Benzyloxy)hexadecan-6-ol (*R*-**12a**). White solid; yield 73%; m.p.: 53–54 °C; [α]_D_^20^ = −1.8 (c 0.5, CH_2_Cl_2_); ^1^H NMR (400 MHz, CDCl_3_): *δ* 7.46–7.17 (5H, m, ArH), 4.50 (2H, s, PhCH_2_O), 3.63–3.53 (1H, m, OCH), 3.47 (2H, t, *J* = 6.6 Hz, OCH_2_), 1.69–1.58 (2H, m, CH_2_), 1.54–1.16 (25H, m, 12 × CH_2_ and OH), 0.89 (3H, t, *J* = 6.7 Hz, CH_3_); ^13^C NMR (100 MHz, CDCl_3_): *δ* 138.7, 128.3, 127.6, 127.5, 72.9, 71.9, 70.3, 37.5, 37.4, 31.9, 29.7, 29.6, 29.6, 29.3, 26.3, 25.6, 25.5, 22.7, 14.1; HRMS (ESI^+^): *m*/*z* calculated for C_23_H_40_NaO_2_^+^: 371.2921; [M + Na]^+^ found: 371.2921.

(*S*)-1-(Benzyloxy)hexadecan-6-ol (*S*-**12a**). White solid; yield 70%; m.p.: 53–54 °C; [α]_D_^20^ = +2.0 (c 1.0, CH_2_Cl_2_).

(*R*)-1-(Benzyloxy)hexadecan-8-ol (*R*-**12b**). White solid; yield 71%; m.p.: 52–54 °C; [α]_D_^20^ = +2.0 (c 1.0, CH_2_Cl_2_); ^1^H NMR (200 MHz, CDCl_3_): *δ* 7.43–7.21 (5H, m, ArH), 4.50 (2H, s, PhCH_2_O), 3.63–3.53 (1H, m, OCH), 3.46 (2H, t, *J* = 6.6 Hz, OCH_2_), 1.71–1.57 (3H, m, CH_2_ and OH), 1.50–1.20 (24H, m, 12 × CH_2_), 0.88 (3H, t, *J* = 6.0 Hz, CH_3_); ^13^C NMR (50 MHz, CDCl_3_): *δ* 138.6, 128.3, 127.6, 127.4, 72.8, 72.0, 70.4, 37.5, 37.4, 31.9, 29.7, 29.6, 29.6, 29.4, 29.3, 26.1, 25.6, 25.6, 22.6, 14.1; HRMS (ESI^+^): *m*/*z* calculated for C_23_H_40_NaO_2_^+^: 371.2921; [M + Na]^+^ found: 371.2921.

(*S*)-1-(Benzyloxy)hexadecan-8-ol (*S*-**12b**). White solid; yield 71%; m.p.: 52–54 °C; [α]_D_^20^ = −2.8 (c 1.0, CH_2_Cl_2_).

(*R*)-16-(Benzyloxy)hexadecan-6-ol (*R*-**12c**). White low melting point solid; yield 83%; [α]_D_^20^ = +2.0 (c 1.0, CH_2_Cl_2_); ^1^H NMR (400 MHz, CDCl_3_): *δ* 7.37–7.25 (5H, m, ArH), 4.50 (2H, s, PhCH_2_O), 3.67–3.52 (1H, m, OCH), 3.46 (2H, t, *J* = 6.6 Hz, OCH_2_), 1.65–1.57 (2H, m, CH_2_), 1.50–1.23 (25H, m, 12 × CH_2_ and OH), 0.89 (3H, t, *J* = 6.4 Hz, CH_3_); ^13^C NMR (100 MHz, CDCl_3_): *δ* 138.7, 128.3, 127.6, 127.4, 72.8, 72.0, 70.5, 37.5, 37.4, 31.9, 29.8, 29.7, 29.6, 29.6, 29.5, 29.5, 26.2, 25.6, 25.3, 22.6, 14.0; HRMS (ESI^+^): *m*/*z* calculated for C_23_H_40_NaO_2_^+^: 371.2921; [M + Na]^+^ found: 371.2920.

(*S*)-16-(Benzyloxy)hexadecan-6-ol (*S*-**12c**). White low melting point solid; yield 82%; [α]_D_^20^ = −2.4 (c 0.5, CH_2_Cl_2_).

(*R*)-1-(Benzyloxy)heptadecan-7-ol (*R*-**12d**). White solid; yield 72%; m.p.: 58–60 °C; [α]_D_^20^ = −2.0 (c 0.5, CH_2_Cl_2_); ^1^H NMR (400 MHz, CDCl_3_): *δ* 7.50–7.18 (5H, m, ArH), 4.50 (2H, s, PhCH_2_O), 3.62–3.53 (1H, m, OCH), 3.47 (2H, t, *J* = 6.6 Hz, OCH_2_), 1.66–1.58 (2H, m, CH_2_), 1.51–1.21 (27H, m, 13 × CH_2_ and OH), 0.88 (3H, t, *J* = 6.5 Hz, CH_3_); ^13^C NMR (100 MHz, CDCl_3_): *δ* 138.7, 128.3, 127.6, 127.5, 72.9, 72.0, 70.4, 37.5, 37.4, 31.9, 29.7, 29.6, 29.5, 29.3, 26.2, 25.6, 25.6, 22.7, 14.1; HRMS (ESI^+^): *m*/*z* calculated for C_24_H_42_NaO_2_^+^: 385.3077; [M + Na]^+^ found: 385.3079.

(*S*)-1-(Benzyloxy)heptadecan-7-ol (*S*-**12d**). White solid; yield 75%; m.p.: 58–60 °C; [α]_D_^20^ = +2.5 (c 0.5, CH_2_Cl_2_).

(*R*)-1-(Benzyloxy)octadecan-8-ol (*R*-**12e**). White solid; yield 79%; m.p.: 53–56 °C; [α]_D_^20^ = −1.8 (c 0.4, CH_2_Cl_2_); ^1^H NMR (400 MHz, CDCl_3_): *δ* 7.59–7.08 (5H, m, ArH), 4.51 (2H, s, PhCH_2_O), 3.69–3.52 (1H, m, OCH), 3.48 (2H, t, *J* = 7.0 Hz, OCH_2_), 1.73–1.55 (3H, m, CH_2_ and OH), 1.55–1.12 (28H, m, 14 × CH_2_), 0.89 (3H, t, *J* = 6.2 Hz, CH_3_); ^13^C NMR (100 MHz, CDCl_3_): *δ* 138.7, 128.3, 127.6, 127.4, 72.8, 72.0, 70.5, 37.5, 37.5, 31.9, 29.7, 29.6, 29.4, 29.3, 26.1, 25.6, 25.6, 22.7, 14.1; HRMS (ESI^+^): *m*/*z* calculated for C_25_H_44_NaO_2_^+^: 399.3234; [M + Na]^+^ found: 399.3240.

(*S*)-1-(Benzyloxy)octadecan-8-ol (*S*-**12e**). White solid; yield 76%; m.p.: 53–56 °C; [α]_D_^20^ = +2.0 (c 1.0, CH_2_Cl_2_).

(*R*)-18-(Benzyloxy)octadecan-8-ol (*R*-**12f**). White solid; yield 84%; m.p.: 47–49 °C; [α]_D_^20^ = +1.4 (c 1.2, CH_2_Cl_2_); ^1^H NMR (400 MHz, CDCl_3_): *δ* 7.40–7.22 (5H, m, ArH), 4.50 (2H, s, PhCH_2_O), 3.63–3.54 (1H, m, OCH), 3.46 (2H, t, *J* = 6.7 Hz, OCH_2_), 1.65–1.58 (2H, m, CH_2_), 1.48–1.24 (29H, m, 14 × CH_2_ and OH), 0.88 (3H, t, *J* = 6.8 Hz, CH_3_); ^13^C NMR (100 MHz, CDCl_3_): *δ* 138.7, 128.3, 127.6, 127.4, 72.8, 72.0, 70.5, 37.5, 31.8, 29.8, 29.7, 29.7, 29.6, 29.6, 29.5, 29.5, 29.3, 26.2, 25.6, 22.6, 14.1; HRMS (ESI^+^): *m*/*z* calculated for C_25_H_44_NaO_2_^+^: 399.3234; [M + Na]^+^ found: 399.3234.

(*S*)-18-(Benzyloxy)octadecan-8-ol (*S*-**12f**). White solid; yield 82%; m.p.: 47–49 °C; [α]_D_^20^ = −1.8 (c 0.8, CH_2_Cl_2_).

### 2.11. General Procedure for the Removal of Benzyl Group

To a round bottom flask containing the benzyl-protected alcohol (1.00 mmol) in MeOH (10 mL), 10% palladium on activated charcoal was added and the reaction mixture was left stirring under a hydrogen atmosphere for 16 h. After filtration through a celite pad, the solvent was removed in vacuo, leading to the isolation of the desired alcohol without further purification.

(*R*)-1-Hydroxyhexadecan-6-yl acetate (*R*-**14a**). Colorless oil; yield 77%; [α]_D_^20^ = +1.2 (c 2.0, CH_2_Cl_2_); ^1^H NMR (400 MHz, CDCl_3_): *δ* 4.94–4.76 (1H, m, OCH), 3.61 (2H, t, *J* = 6.5 Hz, C*H*_2_OH), 2.02 (3H, s, COCH_3_), 1.67 (1H, s, OH), 1.59–1.46 (6H, m, 3 × CH_2_), 1.40–1.19 (20H, m, 10 × CH_2_), 0.86 (3H, t, *J* = 6.7 Hz, CH_3_); ^13^C NMR (100 MHz, CDCl_3_): *δ* 171.0, 74.2, 62.7, 34.1, 34.1, 32.6, 31.9, 29.6, 29.5, 29.5, 29.3, 25.5, 25.3, 25.0, 22.6, 21.2, 14.1; HRMS (ESI^+^): *m*/*z* calculated for C_18_H_36_NaO_3_^+^: 323.2557; [M + Na]^+^ found: 323.2557.

(*S*)-1-Hydroxyhexadecan-6-yl acetate (*S*-**14a**). Colorless oil; yield 84%; [α]_D_^20^ = −1.0, (c 1.0, CH_2_Cl_2_).

(*R*)-1-Hydroxyhexadecan-8-yl acetate (*R*-**14b**). Colorless oil; yield 74%; [α]_D_^20^ = +1.0 (c 0.5, CHCl_3_), [α]_D_^20^ lit. [24] = +0.03 (c 6.311, CH_3_OH); ^1^H NMR (200 MHz, CDCl_3_): *δ* 4.91–4.78 (1H, m, OCH), 3.63 (2H, t, *J* = 6.4 Hz, C*H*_2_OH), 2.03 (3H, s, COCH_3_), 1.61–1.43 (7H, m, 3 × CH_2_ and OH), 1.43–1.10 (20H, m, 10 × CH_2_), 0.87 (3H, t, *J* = 6.0 Hz, CH_3_); ^13^C NMR (50 MHz, CDCl_3_): *δ* 171.0, 74.4, 62.9, 34.1, 32.7, 31.8, 29.5, 29.5, 29.4, 29.3, 29.2, 25.6, 25.3, 25.2, 22.6, 21.3, 14.1; HRMS (ESI^+^): *m*/*z* calculated for C_18_H_36_NaO_3_^+^: 323.2557; [M + Na]^+^ found: 323.2557.

(*S*)-1-Hydroxyhexadecan-8-yl acetate (*S*-**14b**) [17]. Colorless oil; yield 83%; [α]_D_^20^ = −1.5 (c 0.5, CHCl_3_), [α]_D_^22^ lit. = −1.83 (c 2.07, CHCl_3_).

(*R*)-16-Hydroxyhexadecan-6-yl acetate (*R*-**14c**). Colorless oil; yield 89%; [α]_D_^20^ = +1.7 (c 0.6, CH_2_Cl_2_); ^1^H NMR (400 MHz, CDCl_3_): *δ* 5.04–4.72 (1H, m, OCH), 3.63 (2H, t, *J* = 6.6 Hz, C*H*_2_OH), 2.03 (3H, s, COCH_3_), 1.64–1.47 (7H, m, 3 × CH_2_ and OH), 1.37–1.20 (20H, m, 10 × CH_2_), 0.87 (3H, t, *J* = 6.7 Hz, CH_3_); ^13^C NMR (100 MHz, CDCl_3_): *δ* 171.0, 74.4, 63.1, 34.1, 34.1, 32.8, 31.7, 29.5, 29.5, 29.5, 29.4, 29.4, 25.7, 25.3, 25.0, 22.5, 21.3, 14.0; HRMS (ESI^+^): *m*/*z* calculated for C_18_H_36_NaO_3_^+^: 323.2557; [M + Na]^+^ found: 323.2557.

(*S*)-16-Hydroxyhexadecan-6-yl acetate (*S*-**14c**). Colorless oil; yield 92%; [α]_D_^20^ = −2.0 (c 0.5, CH_2_Cl_2_).

(*R*)-1-Hydroxyheptadecan-7-yl acetate (*R*-**14d**). Colorless oil; yield 91%; [α]_D_^20^ = +1.8 (c 1.6, CH_2_Cl_2_); ^1^H NMR (400 MHz, CDCl_3_): *δ* 4.92–4.78 (1H, m, OCH), 3.62 (2H, t, *J* = 6.6 Hz, C*H*_2_OH), 2.02 (3H, s, COCH_3_), 1.59–1.46 (7H, m, 3 × CH_2_ and OH), 1.37–1.21 (22H, m, 11 × CH_2_), 0.87 (3H, t, *J* = 6.6 Hz, CH_3_); ^13^C NMR (100 MHz, CDCl_3_): *δ* 171.0, 74.4, 62.9, 34.1, 34.0, 32.6, 31.9, 29.6, 29.5, 29.5, 29.3, 29.2, 25.6, 25.3, 25.2, 22.6, 21.2, 14.1; HRMS (ESI^+^): *m*/*z* calculated for C_19_H_38_NaO_3_^+^: 337.2713; [M + Na]^+^ found: 337.2712.

(*S*)-1-Hydroxyheptadecan-7-yl acetate (*S*-**14d**). Colorless oil; yield 96%; [α]_D_^20^ = −2.4 (c 2.0, CH_2_Cl_2_).

(*R*)-1-Hydroxyoctadecan-8-yl acetate (*R*-**14e**). Colorless oil; yield 90%; [α]_D_^20^ = +1.5 (c 1.0, CH_2_Cl_2_); ^1^H NMR (400 MHz, CDCl_3_): *δ* 4.93–4.74 (1H, m, OCH), 3.62 (2H, t, *J* = 6.6 Hz, C*H*_2_OH), 2.02 (3H, s, COCH_3_), 1.59–1.46 (7H, m, 3 × CH_2_ and OH), 1.42–1.13 (24H, m, 12 × CH_2_), 0.87 (3H, t, *J* = 6.5 Hz, CH_3_); ^13^C NMR (100 MHz, CDCl_3_): *δ* 171.0, 74.4, 62.9, 34.1, 34.1, 32.7, 31.9, 29.6, 29.5, 29.5, 29.4, 29.3, 29.3, 25.6, 25.3, 25.2, 22.6, 21.2, 14.1; HRMS (ESI^+^): *m*/*z* calculated for C_20_H_40_NaO_3_^+^: 351.2870; [M + Na]^+^ found: 351.2875.

(*S*)-1-Hydroxyoctadecan-8-yl acetate (*S*-**14e**). Colorless oil; yield 85%; [α]_D_^20^ = −2.0 (c 1.4, CH_2_Cl_2_).

(*R*)-18-Hydroxyoctadecan-8-yl acetate (*R*-**14f**). Colorless oil; yield 90%; [α]_D_^20^ = +1.9 (c 1.0, CH_2_Cl_2_); ^1^H NMR (400 MHz, CDCl_3_): *δ* 4.93–4.78 (1H, m, OCH), 3.63 (2H, t, *J* = 6.6 Hz, C*H*_2_OH), 2.03 (3H, s, COCH_3_), 1.60–1.47 (6H, m, 3 × CH_2_), 1.40–1.19 (25H, m, 12 × CH_2_ and OH), 0.87 (3H, t, *J* = 6.9 Hz, CH_3_); ^13^C NMR (100 MHz, CDCl_3_): *δ* 171.0, 74.5, 63.1, 34.1, 32.8, 31.8, 29.5, 29.5, 29.5, 29.4, 29.4, 29.2, 25.7, 25.3, 25.3, 22.6, 21.3, 14.1; HRMS (ESI^+^): *m*/*z* calculated for C_20_H_40_NaO_3_^+^: 351.2870; [M + Na]^+^ found: 351.2870.

(*S*)-18-Hydroxyoctadecan-8-yl acetate (*S*-**14f**). Colorless oil; yield 89%; [α]_D_^20^ = −2.2 (c 0.7, CH_2_Cl_2_).

### 2.12. General Method for the Synthesis of Alkanes from Alkynes Using Rosenmund Catalyst

To a round-bottom flask containing the alkyne (1.00 mmol) in EtOAc (10 mL), Rosenmund catalyst (Pd/BaSO_4_) (10% *w*/*w*) was added and the reaction mixture was left stirring under a hydrogen atmosphere for 3 h. Then, the reaction mixture was filtered through a celite pad and the solvent was evaporated in vacuo. Flash silica column chromatography eluting with petroleum ether (bp 40−60 °C):ethyl acetate (100:0–95:5–90:10) followed, leading to the isolation of compounds **20** and **27**.

(*S*)-1-((*tert*-Butyldimethylsilyl)oxy)octadecan-6-ol (**20**). Colorless oil; yield 92%; [α]_D_^20^ = +1.0 (c 1.0, CH_2_Cl_2_); ^1^H NMR (200 MHz, CDCl_3_): *δ* 3.64–3.49 (3H, m, CH_2_OTBDMS and OCH), 1.63–1.17 (31H, m, 15 × CH_2_ and OH), 0.96–0.77 (12H, m, 4 × CH_3_), 0.03 (6H, s, 2 × CH_3_); ^13^C NMR (50 MHz, CDCl_3_): *δ* 71.8, 63.2, 37.4, 32.8, 31.9, 29.7, 29.6, 29.3, 25.9, 25.9, 25.6, 25.4, 22.7, 18.3, 14.1, −5.3; HRMS (ESI^+^): *m*/*z* calculated for C_24_H_52_NaO_2_Si^+^: 423.3629; [M + Na]^+^ found: 423.3629.

(*R*)-1-((*tert*-Butyldimethylsilyl)oxy)octadecan-6-ol (**27**). Colorless oil; yield 91%; [α]_D_^20^ = −1.8 (c 2.5, CH_2_Cl_2_).

### 2.13. Biological Assays

#### 2.13.1. Cell Culture and Reagents

A549 and SF268 cell lines were maintained in Dulbecco’s Modified Eagle Medium (DMEM) (4.5 g/L glucose, Biosera, Nuaille, France), which was supplemented with 10% heat-inactivated fetal bovine serum (FBS) (Biosera, Nuaille, France) and penicillin/streptomycin (100 mg/mL; Invitrogen, Carlsbad, CA, USA). The cells were incubated in a humidified incubator at 37 °C in 5% CO_2_.

#### 2.13.2. Cell Viability Assays

MTT [3-(4,5-dimethylthiazol-2-yl)-2,5-diphenyl-2H-tetrazolium bromide] assay: MTT experiments were performed in triplicate and repeated at least three times. First, cells (3 × 10^5^) were seeded in a 96-well culture plate, incubated for 24 h and then treated with 10 µM, 25 µM, 35 µM, 50 µM, 75 µM and 100 µM of HFAs and incubated for 72 h (in all concentrations, dimethyl sulfoxide (DMSO) was 0.5%). Control cells were treated with 0.5% DMSO in culture medium. After treatment, the medium was removed and the cells were incubated with MTT reagent (Sigma) (0.25 mg/mL) at 37 °C for 3 h. The resulting formazan crystals were solubilized by removal of the MTT and addition of 100 μL DMSO per well. The optical density at 570 nm was measured with an Enzyme-linked Immunosorbent Assay (ELISA) reader: IRMECO ELx800 by BioTek (BioTek Instruments, Winooski, VT, USA). Cell viability was calculated by the formula: cell viability (%) = (absorbance of the treated wells)/(absorbance of the DMSO control wells) × 100%.

Cell growth inhibition analysis: Microsoft Excel was used for data analysis (Office Professional Plus 2016). The background absorbance at 690 nm was subtracted from the corresponding values at 570 nm and the average of three repeats for each condition was determined. The resulting dataset was normalized to DMSO control cells as the 100% survival value. Inhibition curves were generated with the use of GraphPad Prism version 6.01 and the corresponding IC_50_ values were calculated from the resultant plot. Two-way ANOVA statistical analyses with multiple comparisons were performed on the dataset, in order to compare the different concentrations of the compounds investigated to the DMSO control.

#### 2.13.3. Immunoblotting

Using RIPA’s (Radioimmunoprecipitation) lysis buffer, total protein was extracted from the treated cells. The homogenates were centrifuged for 10 min at 13,000 rpm in ice (4 °C). A Bradford protein assay was used to determine the protein concentration in the collected supernatants (Bio-Rad protein assay). In each case, 35 μg of protein samples were loaded onto SDS-PAGE gels (sodium dodecyl-sulfate polyacrylamide gel electrophoresis) and transferred, using a semi-dry transfer technique, to nitrocellulose membranes from Amersham (Bio-Rad, Hercules, CA, USA). Five percent bovine serum albumin (BSA) (Applichem, Darmstadt, Germany, A1391), diluted in Tris-buffered saline (1×) containing 0.1% Tween-20, was used to block the membranes for an hour at room temperature. Subsequently, the membranes were incubated with the primary antibody solutions at 4 °C overnight, and then secondary antibodies were added and membranes were incubated at room temperature for 1.5 h. The primary antibodies for the Western blots were anti-STAT3 (Santa Cruz Biotech. Inc., Santa Cruz, CA, USA, sc-482) (1:1000 dilution), anti-acetylated Histone 3 [ac Lys14, ac Lys9] (Novus Bio., Centennial, CO, USA, NBP2-59181) (1:1000 dilution), anti-acetylated α-tubulin (OriGene Technologies, Inc., Rockville, MD, USA, TA385485) (1:1000 dilution) and mouse anti-beta actin (Sigma, A5441) (1:20.000 dilution). The secondary antibodies were rabbit anti-mouse IgG (Sigma, A9044) (1:20.000 dilution), and goat anti-rabbit IgG (Sigma, A6154) (1:10.000 dilution).

## 3. Results and Discussion

### 3.1. Synthesis of HFAs

We have previously presented the asymmetric synthesis of fatty acid esters of hydroxy fatty acids (FAHFAs) and their precursors, saturated HFAs [5,28], as well as the asymmetric synthesis of 3HFAs [29]. In this work, we present a synthetic methodology leading to two unsaturated HFAs, namely (*S*,*Z*)-7-hydroxyoctadec-9-enoic acid and (*S*,*Z*)-7-hydroxyhexadec-9-enoic acid. The asymmetric synthesis involves an organocatalytic protocol as the key step, using MacMillan’s third-generation imidazolidinone ((2*S*,5*R*)-2-(*tert*-butyl)-3,5-dimethylimidazolidin-4-one trifluoroacetate) as the catalyst for the formation of chiral terminal (*S*)-epoxides, enabling the introduction of the chiral center of HFAs backbone [5,28] and leading to products in high enantiomeric excesses (*ee*’s 90–95%).

**Scheme 1:**
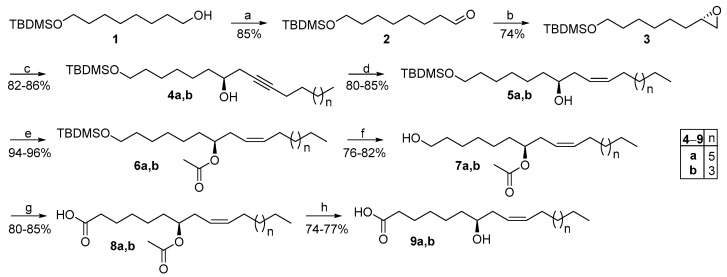
Asymmetric synthesis of unsaturated HFAs. (a) PCC, dry CH_2_Cl_2_; (b) i. (2*S*,5*R*)-2-(*tert*-butyl)-3,5-dimethylimidazolidin-4-one trifluoroacetate (20%), 2,3,4,5,6,6-hexachlorocyclohexa-2,4-dien-1-one, THF; ii. NaBH_4_, EtOH, iii. KOH/EtOH/H_2_O; (c) 1-decyne or 1-octyne, n-BuLi, BF_3_·OEt_2_, dry THF; (d) Lindlar’s cat. (5%), H_2_, quinoline, CH_3_OH; (e) AcCl, dry pyridine, dry CH_2_Cl_2_; (f) TBAF, dry THF; (g) Jones reagent, acetone; (h) LiOH·H_2_O, THF:H_2_O.

As depicted in Scheme 1, mono-*tert*-butyldimethylsilyl (TBDMS)-protected α,ω-diol **1** underwent oxidation using pyridinium chlorochromate (PCC), for the formation of aldehyde **2**, which was converted to a chiral terminal epoxide following a simple, one-pot, three-step organocatalytic protocol. The aldehyde was converted to an α-chloro aldehyde by utilization of (2*S*,5*R*)-2-(*tert*-butyl)-3,5-dimethylimidazolidin-4-one trifluoroacetate as the catalyst and 2,3,4,5,6,6-hexachlorocyclohexa-2,4-dien-1-one as the chlorinating agent. Then, reduction by NaBH_4_ was carried out and the subsequent epoxide-ring formation occurred through treatment with an aqueous KOH solution in ethanol, following a S_N_2 mechanism. The succeeding step, consisting of the alkynylation of epoxide **3** using commercially available alkynes, 1-decyne or 1-octyne, in the presence of n-BuLi and Lewis acid, boron trifluoride diethyl etherate [30], was inspired by Durand and co-workers’ work [31], enabling the synthesis of hydroxy alkynes **4a**,**b**. Then, hydrogenation was carried out using Lindlar’s catalyst and quinoline under an H_2_ atmosphere, for the conversion of hydroxy alkynes **4a**,**b** to *cis*-hydroxy alkenes **5a**,**b**. The acetylation of compounds **5a**,**b** for the protection of the hydroxyl group and consequent deprotection of the TBDMS-group with tetra-n-butylammonium fluoride (TBAF) led to the formation of intermediates **7a**,**b**. The oxidation of alcohols **7a**,**b** with Jones reagent, followed by the saponification of esters **8a**,**b** afforded the unsaturated HFAs **9a**,**b** high yields.

**Scheme 2:**
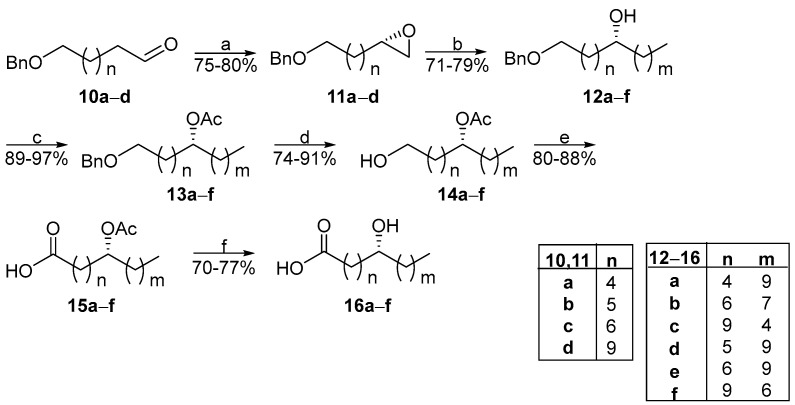
Asymmetric synthesis of (*R*)-6HPA, (*R*)-8HPA, (*R*)-11HPA, (*R*)-7HMA, (*R*)-8HSA and (*R*)-11HSA. (a) i. (2*S*,5*R*)-2-(*tert*-butyl)-3,5-dimethylimidazolidin-4-one trifluoroacetate (20%), 2,3,4,5,6,6-hexachlorocyclohexa-2,4-dien-1-one, THF; ii. NaBH_4_, EtOH; iii. KOH/EtOH/H_2_O; (b) RMgX, CuI, dry THF; (c) AcCl, dry pyridine, dry CH_2_Cl_2_; (d) H_2_, 10% Pd/C, EtOH; (e) Jones reagent, acetone; (f) LiOH·H_2_O, THF:H_2_O.

The asymmetric synthesis of saturated (*R*)-HFAs is presented in Scheme 2. The asymmetric epoxidation of mono-benzyl-protected aldehydes **10a**–**d** led to the formation of (*S*)-epoxides **11a**–**d** in *ee’s* varying from 90% to 95%, which were then treated with the appropriate Grignard reagent in the presence of CuI for the formation of 16-, 17- or 18-carbon atom aliphatic chain secondary alcohols **12a**–**f**, with the hydroxyl group at positions 6-, 7-, 8- and 11-. The acetylation of compounds **12a**–**f**, the hydrogenation of **13a**–**f** for the deprotection of the benzyl group and the subsequent Jones oxidation enabled the isolation of acetoxy-palmitic, margaric or oleic acids **15a**–**f**, the hydrolysis of which with lithium hydroxide afforded the desired HFAs **16a**–**f**. The same procedure was followed for the synthesis of (*S*)-HFAs, using (2*R*,5*S*)-2-(*tert*-butyl)-3,5-dimethylimidazolidin-4-one trifluoroacetate as the chlorination catalyst, permitting the synthesis of (*R*)-epoxides in *ee*’s varying from 90% to 93%.

**Scheme 3:**
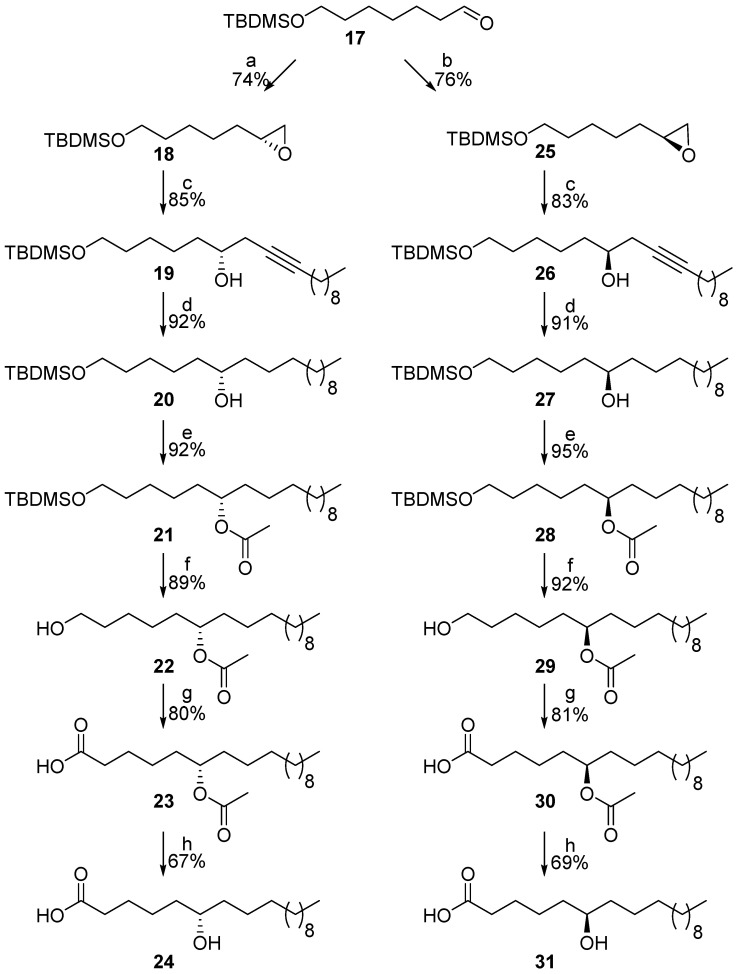
Asymmetric synthesis of (*S*)- and (*R*)-6HSAs. (a) i. (2*R*,5*S*)-2-(*tert*-butyl)-3,5-dimethylimidazolidin-4-one trifluoroacetate (20%), 2,3,4,5,6,6-hexachlorocyclohexa-2,4-dien-1-one, THF; ii. NaBH_4_, EtOH; iii. KOH/EtOH/H_2_O; (b) i. (2*S*,5*R*)-2-(*tert*-butyl)-3,5-dimethylimidazolidin-4-one trifluoroacetate (20%), 2,3,4,5,6,6-hexachlorocyclohexa-2,4-dien-1-one, THF; ii. NaBH_4_, EtOH; iii. KOH/EtOH/H_2_O; (c) 1-undecyne, n-BuLi, BF_3_·OEt_2_, dry THF; (d) 10% Pd/BaSO_4_, H_2_, EtOAc; (e) AcCl, dry pyridine, dry CH_2_Cl_2_; (f) TBAF, dry THF; (g) Jones reagent, acetone; (h) LiOH·H_2_O, THF:H_2_O.

In case the above-mentioned synthetic methodology of saturated HFAs was not feasible, due to the poor availability of the starting materials, another synthetic route to obtain HFAs was developed, which constituted a combination of the previous two. This route was used to synthesize (*S*)- and (*R*)-6HSAs (Scheme 3). Chiral epoxides **18** and **25**, derived from the previously described organocatalytic protocol, were submitted to alkynylation after the addition of commercially available 1-undecyne, alongside with n-BuLi and BF_3_·OEt_2_. Hydroxy alkynes **19** and **26** were then hydrogenated over Rosenmund catalyst, without the addition of any amine, as described by Balas et al. [31], to form the fully saturated compounds **20** and **27**. Then, a similar synthetic pathway was followed (including the acetylation of the secondary hydroxyl group, TBDMS-deprotection with TBAF reagent, Jones oxidation and acetyl deprotection using LiOH), allowing the preparation of HFAs **24** and **31**.

### 3.2. Antiproliferative Activity

**Figure 2:**
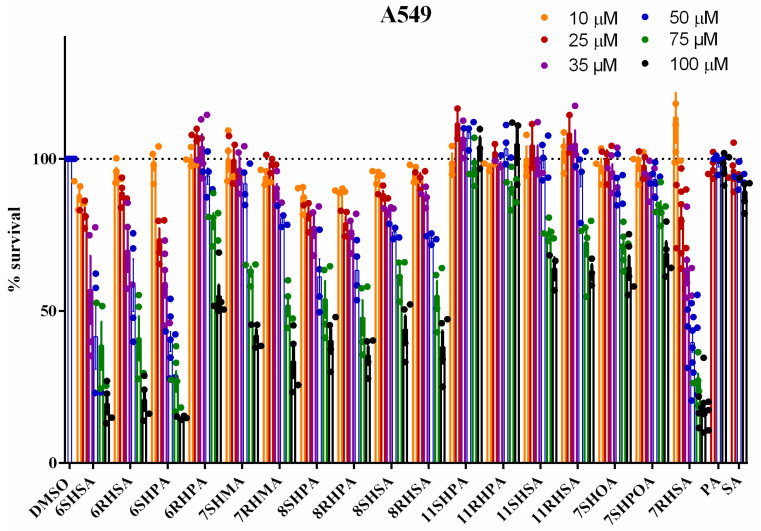
Antiproliferative activity of various HFAs (human lung cancer cell line A549). Cells were treated with increasing concentrations (10 µM, 25 µM, 35 µM, 50 µM, 75 µM, 100 µM) of the test compounds for 72 h and cell viability was determined by the MTT assay for a minimum of three experiments (AVG ± SEM).

The antiproliferative activity of six enantiomers of HSAs (6-, 8-, 11-HSAs), six enantiomers of HPAs (6-, 8-, 11-HPAs), two enantiomers of hydroxymargaric acid (7HMA), 7-(*S*)-hydroxyoleic acid (7SHOA) and 7-(*S*)-hydroxypalmitoleic acid (7SHPOA) was studied on A549 cells and the results are summarized in Figure 2. Data on 7RHSA, palmitic acid (PA) and stearic acid (SA) were included for comparison purposes. The A549 cell line was selected because it had been used in our previous work [5], thus permitting direct comparison of the effect of the newly synthesized analogs with the previous results. In addition, the antitumor drug 2-hydroxyoleic acid (2HOA, trade name Minerval), possessing a similar structure, has been shown to present selectivity over various cancer cell lines, exhibiting a more potent effect on A549 (IC_50_ = 90 μΜ) than, for example, U87-MG (IC_50_ = 400 μM) [32].

The position of the hydroxyl group in the fatty acid chain drastically influences the *in vitro* potency. Interestingly, 6HFAs exhibited the most potent antiproliferative activity at concentrations over 35 μM, while 7HMAs, 8HPAs and 8HSAs exhibited moderate antiproliferative activity at concentrations over 50 μM. When the hydroxyl group was at position 11- of the aliphatic chain, no activity was observed for the corresponding HPAs, while the presence of a double bond in the aliphatic chain resulted in very weak activity. All statistical comparisons for the antiproliferative activity of the investigated HFAs are included in Table S1 (Supplementary Materials).

**Figure 3:**
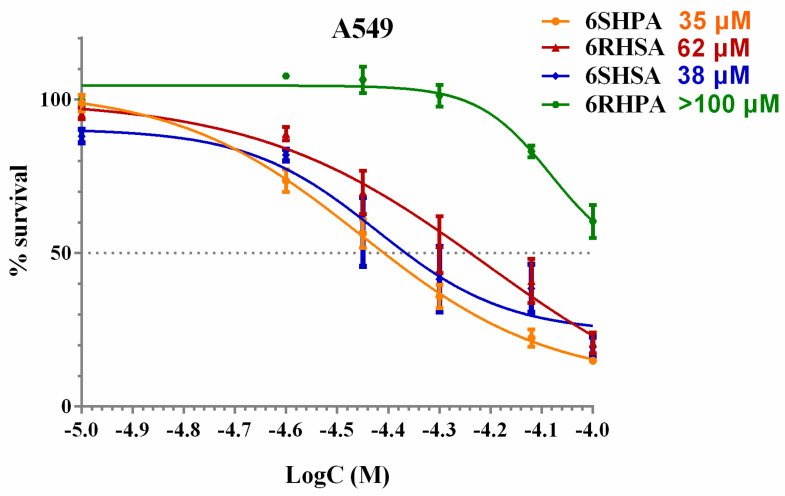
Inhibition curves of enantiomers 6SHPA, 6RHSA and 6SHSA in A549 cells. IC_50_ values were derived from the dose–response relationship for a minimum of six experiments (95% CI log IC_50_ −4.53 to −4.38, −4.71 to −3.71 and −4.57 to −4.28, respectively).

For A549 cancer cells, the IC_50_ values for the enantiomers 6SHPA, 6RHSA and 6SHSA were determined from the curves depicted in Figure 3 and vary from 35 to 62 μM.

**Figure 4:**
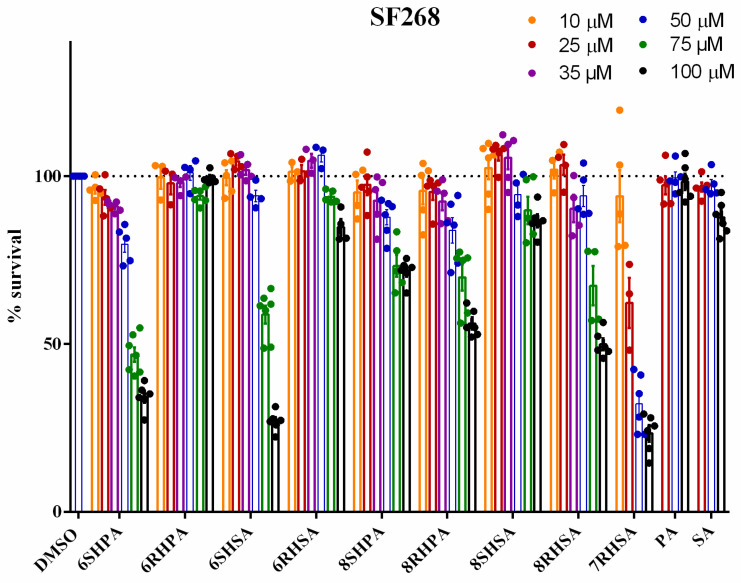
Antiproliferative activity of HFAs (human astrocytoma cell line SF268). Cells were treated with increasing concentrations (10 µM, 25 µM, 35 µM, 50 µM, 75 µM, 100 µM) of the test compounds for 72 h and cell viability was determined by the MTT assay for a minimum of three experiments (AVG ± SEM).

**Figure 5:**
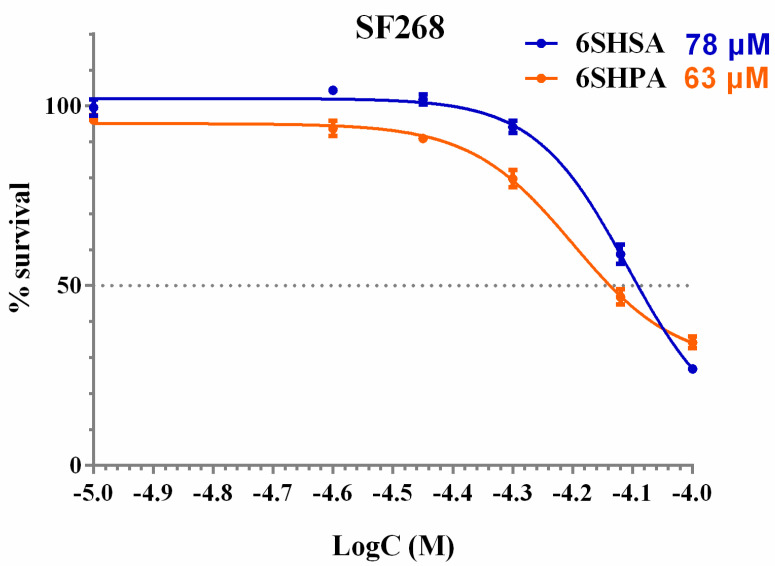
Inhibition curves of enantiomers 6SHSA and 6SHPA in SF268 cells. IC_50_ values were derived from the dose–response relationship for a minimum of six experiments (95% CI log IC_50_ −4.15 to −4.06 and −4.23 to −4.17, respectively).

Based on these results, the enantiomers of 6HPA, 6HSA, 8HPA and 8HSA were selected for evaluation on an SF268 human astrocytoma cell line. The results are presented in Figure 4, and 7RHSA, PA and SA were included for comparison purposes. The IC_50_ values for the enantiomers 6SHPA and 6SHSA were determined from the curves depicted in Figure 5.

For both cell lines, (*S*)-6-hydroxypalmitic acid (6SHPA) was found to exhibit the most potent antiproliferative activity, with IC_50_ values of 35 and 63 μΜ for A549 and SF268 cells, respectively. The (*R*)-enantiomers of 6HPA and 6HSA were found to be considerably less potent than the corresponding (*S*)-enantiomers and, in the case of 6RHPA, very weak potency was observed in A549 cells and no potency in SF268 cells. PA and SA were also studied for their potential antiproliferative activity against A549 and SF268 cells for comparison purposes. As shown in Figure 2, both compounds did not inhibit the proliferation of A549 cells. In addition, PA did not exhibit any activity against SF268 cells (Figure 4), while SA exhibited a very weak effect at the highest concentration (100 μM). These findings indicate that the presence of the hydroxyl group is crucial for the antiproliferative effect of HFAs.

### 3.3. Western Blot Analysis

**Figure 6:**
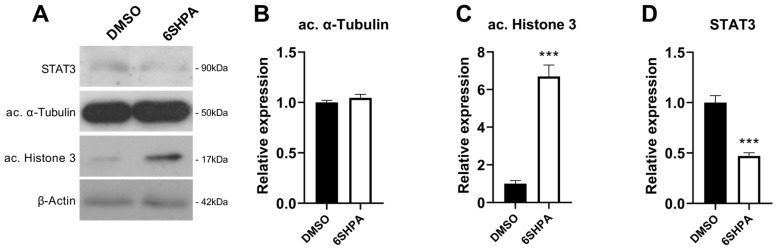
6SHPA induces the acetylation of histone 3 and inhibits STAT3 expression in A549 cells. Cells treated with 6SHPA (50 µM) versus DMSO and assessed after 48 h. (**A**,**B**) Western blot analysis for acetylated α-tubulin upon 6SHPA treatment. (**A**,**C**) Western blot analysis for acetylated histone 3 upon 6SHPA treatment. (**A**,**D**) Western blot analysis for STAT3 expression upon 6SHPA treatment. Protein expression levels quantitated by using the ImageJ software. All experiments were repeated three times (*n* = 3). For all cases, *** *p* < 0.001. Original images can be found in Supplementary File S1.

As we have demonstrated in our previous work, saturated HFAs can lead to cell cycle arrest and do not promote apoptosis, as reflected by the levels of phosphorylated H3 and activated caspase 3 [5]. Additionally, through real-time reverse transcription-quantitative polymerase chain reaction (RT-qPCR) and Western Blot analysis, 7RHSA was previously found to considerably reduce the levels of transcription regulator signal transducer and activator of transcription 3 (STAT3), a highly important regulator of tumor development and its resistance to treatment [33]. To corroborate these findings, the most potent compound (6SHPA) in the current study was tested for its ability to inhibit STAT3 expression, using Western Blot analysis (Figure 6). Importantly, 6SHPA led to a significant reduction in STAT3 expression, as depicted in Figure 6A,D. Subsequently, 6SHPA was tested for its ability to induce the acetylation of histone H3 and α-tubulin, using Western Blot analysis (Figure 6). Evidently, 6SHPA increased the levels of acetylated histone H3 (Figure 6A,C), with no significant changes found in acetylated α-tubulin levels (Figure 6A,B). These results indicate that 6SHPA may act as a class I Histone Deacetylase (HDAC) inhibitor and are in accordance with a previous study reporting that another HFA (9RHSA) was found to be an inhibitor of HDAC1, 2 and 3 of class I HDACs [33]. Moreover, its potential action as a class I HDAC inhibitor may also explain the antiproliferative action attributed to this compound.

## 4. Discussion

In our previous work [5], we synthesized and tested a series of HPA and HSA regio-isomers with the hydroxyl functionality at positions 7-, 9- and 10-, as well as 2RHSA and 12RHSA. We demonstrated that the (*R*)-enantiomer of 7HSA exhibited the highest potency (IC_50_ values of 38 and 27 μM against human cancer cells A549 and SF268, respectively), followed by 7RHPA (IC_50_ values of 42 and 49 μM against human cancer cells A549 and SF268, respectively). The corresponding (*S*)-enantiomers were slightly less potent, followed by the 9HSA and HPA isomers. 10HPA and HSA isomers exhibited very weak potency, while 2RHSA and 12RHSA exhibited no potency against these cell lines [5]. Calonghi et al. previously identified 9HSA as an antiproliferative agent against HT29 adenocarcinoma cells and demonstrated that the antiproliferative effect brought about by the (*R*)-enantiomer is more pronounced than the (*S*)-enantiomer [34], in accordance with our findings for 7- and 9-HSAs and HPAs [5]. In a recent study, Calonghi et al. evaluated the growth inhibitory effects of a series of HSAs on a panel of cancer cell lines. They found that 5HSA, 7HSA and 9HSA exhibited the highest inhibitory potency (7HSA was the most potent), while 10HSA and 11HSA exhibited a very weak effect and 8HSA showed no inhibitory activity in all cell lines [13]. Our current findings are in agreement with these results, regarding the weak effect of 11HSA enantiomers, while we found that 8HSAs exhibited moderate antiproliferative activity against A549 and SF268 cell lines, which were not utilized by Calogni et al. In addition, the 7HMA enantiomers, which were synthesized and tested for the first time, also exhibited moderate antiproliferative activity against A549 cells, indicating that an odd-numbered long chain is not favored. It seems that shortening the aliphatic chain by just one carbon atom significantly reduces the antiproliferative activity of 7HMA in comparison to 7HSA.

Interestingly, in this work, we found that 6- HPAs and HSAs, which have not been studied so far for their antiproliferative activity, can potently inhibit the proliferation of A549 and SF268 cells, with the (*S*)-enantiomers being the most potent, in particular 6SHPA. These findings are not in accordance with the trend previously observed for 7- and 9-HFAs, where the (*R*)-enantiomers were found to be more potent than the (*S*), though 6SHPA was found to inhibit the expression of the STAT3 protein in the same manner as 7RHSA [5] and to induce the acetylation of histone H3, indicating class I HDAC inhibition.

Regarding unsaturated HFAs, limited derivatives have been studied for their antiproliferative activity so far. Interestingly, 2-hydroxyoleic acid (2HOA) has been investigated as an anticancer drug. It has been reported that it induces glioma cell differentiation and autophagy [35] and that it stimulates signaling and retrograde transport [36]. It seems that 2HOA exerts its antitumor action through membrane fatty acid remodeling [37]. In an effort to extend the knowledge on the bioactivities of the corresponding unsaturated HFAs, we synthesized, for the first time, 7HOA and 7HPOA and tested them against A549 cells. Both compounds showed weak inhibitory activity at concentrations up to 100 μΜ. Consequently, if we compare the activity of 7HOA and 7HPOA with that of 7HSA, we may conclude that the unsaturation of the long chain reduces the antiproliferative activity. In comparison, 2HOA was previously reported to exhibit weak inhibitory activity against A549 cells with an IC_50_ of 90 μΜ [32].

## 5. Conclusions

The interesting antiproliferative properties of saturated HFAs, reported so far, have prompted us to explore various synthetic methods leading to chiral unsaturated and saturated HFAs, in an effort to extend and complete the structure–activity relationship studies. Herein, we present diverse synthetic routes for producing HFAs, using an organocatalytic process for crafting asymmetric terminal epoxides and employing MacMillan’s third-generation imidazolidinone catalyst. By these methods, 16 HFAs, namely 7-(*S*)-hydroxyoleic acid (7SHOA), 7-(*S*)-hydroxypalmitoleic acid (7SHPOA), two enantiomers of hydroxymargaric acid (7HMA), six enantiomers of HSAs (6-, 8-, 11-HSAs) and six enantiomers of HPAs (6-, 8-, 11-HPAs), were synthesized. The antiproliferative activities of the compounds synthesized were evaluated on A549 and SF268 cancer cell lines, showing that 6SHPA presented the most promising results, with IC_50_ values of 35 and 63 μΜ, respectively. Western blot analysis in A549 cells revealed that 6SHPA exhibited selective inhibition of HDAC class I enzymes, since it induced the acetylation of histone 3 in A549 cells, without affecting the levels of acetylated α-tubulin. Furthermore, 6SHPA was found to inhibit STAT3 expression in A549 cells. Overall, the antiproliferative activity of HFAs depends not only on the position of the hydroxyl functionality, but also on the configuration of the asymmetric center and the saturation or unsaturation of the long chain. Further studies are needed to better understand the biological roles of saturated and unsaturated HFAs.

## Data Availability

All data supporting this study are included in the article and Supplementary Materials.

## Supplementary Materials

The following supporting information can be downloaded at: https://www.mdpi.com/article/10.3390/biom14010110/s1, Table S1: two-way ANOVA statistical analysis with multiple comparisons to DMSO for all MTT tests of hydroxy fatty acids in A549 and SF268 human cancer cell lines. ^1^H NMR and ^13^C NMR spectra of the compounds synthesized. (PDF); Supplementary File S1: Western Blot analysis original images.

## References

[1] Zhang W., Hu W., Zhu Q., Niu M., An N., Feng Y., Kawamura K., Fu P. (2024). Hydroxy fatty acids in the surface earth system. Sci. Total Environ..

[2] Hama H. (2010). Fatty acid 2-hydroxylation in mammalian sphingolipid biology. Biochim. Biophys. Acta.

[3] Jin S.-J., Hoppel C.L., Tserng K.-Y. (1992). Incomplete fatty acid oxidation. The production and epimerization of 3-hydroxy fatty acids. J. Biol. Chem..

[4] Raetz C.R.H., Whitfield C. (2002). Lipopolysaccharide endotoxins. Annu. Rev. Biochem..

[5] Kokotou M.G., Kokotos A.C., Gkikas D., Mountanea O.G., Mantzourani C., Almutairi A., Lei X., Ramanadham S., Politis P.K., Kokotos G. (2020). Saturated hydroxy fatty acids exhibit a cell growth inhibitory activity and suppress the cytokine-induced β-cell apoptosis. J. Med. Chem..

[6] Kokotou M.G., Mantzourani C., Bourboula A., Mountanea O.G., Kokotos G. (2020). A liquid chromatography-high resolution mass spectrometry (LC-HRMS) method for the determination of free hydroxy fatty acids in cow and goat milk. Molecules.

[7] Kokotou M.G., Mantzourani C., Batsika C.S., Mountanea O.G., Eleftheriadou I., Kosta O., Tentolouris N., Kokotos G. (2022). Lipidomics analysis of free fatty acids in human plasma of healthy and diabetic subjects by liquid chromatography high resolution mass spectrometry (LC-HRMS). Biomedicines.

[8] Cavalli G., Casali E., Spisni A., Masotti L. (1991). Identification of the peroxidation product hydroxystearic acid in Lewis lung carcinoma cells. Biochem. Biophys. Res. Commun..

[9] Calonghi N., Cappadone C., Pagnotta E., Farruggia G., Buontempo F., Boga C., Brusa G.L., Santucci M.A., Masotti L. (2004). 9-Hydroxystearic acid upregulates p21^WAF1^ in HT29 cancer cells. Biochem. Biophys. Res. Commun..

[10] Calonghi N., Cappadone C., Pagnotta E., Boga C., Bertucci C., Fiori J., Tasco G., Casadio R., Masotti L. (2005). Histone deacetylase 1: A target of 9-hydroxystearic acid in the inhibition of cell growth in human colon cancer. J. Lipid Res..

[11] Calonghi N., Pagnotta E., Parolin C., Tognoli C., Boga C., Masotti L. (2006). 9-Hydroxystearic acid interferes with EGF signaling in a human colon adenocarcinoma. Biochem. Biophys. Res. Commun..

[12] Calonghi N., Boga C., Telese D., Bordoni S., Sartor G., Torsello C., Micheletti G. (2019). Synthesis of 9-hydroxystearic acid derivatives and their antiproliferative activity on HT 29 cancer cells. Molecules.

[13] Calonghi N., Boga C., Nitti P., Telese D., Bordoni S., Farruggia G., Asaro F., Grandi M., Zalambani C., Micheletti G. (2022). Effects of regioisomerism on the antiproliferative activity of hydroxystearic acids on human cancer cell lines. Molecules.

[14] Yadav J.S., Ganganna B., Dutta P., Singarapu K.K. (2014). Synthesis and determination of absolute configuration of α-pyrones isolated from *Penicillium corylophilum*. J. Org. Chem..

[15] Mohapatra D.K., Bhattasali D., Gurjar M.K., Khan M.I., Shashidhara K.S. (2008). First asymmetric total synthesis of penarolide sulfate A1. Eur. J. Org. Chem..

[16] Surup F., Kuhnert E., Böhm A., Pendzialek T., Solga D., Wiebach V., Engler H., Berkessel A., Stadler M., Kalesse M. (2018). The rickiols: 20-, 22-, and 24-membered macrolides from the ascomycete *Hypoxylon rickii*. Chem. Eur. J..

[17] Shimojo M., Matsumoto K., Hatanaka M. (2000). Enzyme-mediated preparation of optically active 1,2-diols bearing a long chain: Enantioselective hydrolysis of cyclic carbonates. Tetrahedron.

[18] Yadav J.S., Srinivasa Rao T., Ravindar K., Subba Reddy B.V. (2009). Total synthesis of (+)-aspicilin from D-mannitol. Synlett.

[19] Saikia B., Devi T.J., Barua N.C. (2013). Stereoselective total synthesis of both (6*R*,9*R*,10*S*,7*E*)- and (6*S*,9*R*,10*S*,7*E*)-epimers of oxylipin (9R,10S,7E)-6,9,10-trihydroxyoctadec-7-enoic acid. Tetrahedron.

[20] Lu B.L., Williams G.M., Verdon D.J., Dunbar P.R., Brimble M.A. (2020). Synthesis and evaluation of novel TLR2 agonists as potential adjuvants for cancer vaccines. J. Med. Chem..

[21] Masaoka Y., Sakakibara M., Mori K. (1982). Synthesis and absolute configuration of (+)-8-hydroxyhexadecanoic acid, an endogenous inhibitor for spore germination in *Lygodium japonicum*. Agric. Biol. Chem..

[22] Lu S.-F., O’yang Q., Guo Z.-W., Yu B., Hui Y.-Z. (1997). Total synthesis of tricolorin A. J. Org. Chem..

[23] Ebert C., Felluga F., Forzato C., Foscato M., Gardossi L., Nitti P., Pitacco G., Boga C., Caruana P., Micheletti G. (2012). Enzymatic kinetic resolution of hydroxystearic acids: A combined experimental and molecular modelling investigation. J. Mol. Catal. B Enzym..

[24] Sugai T., Mori K. (1984). Both enantiomers of 8-hydroxyhexadecanoic acid inhibit the spore germination of *Lygodium japonicum*. Agric. Biol. Chem..

[25] Kitagawa I., Baek N.-I., Kawashima K., Yokokawa Y., Yoshikawa M., Ohashi K., Shibuya H. (1996). Indonesian medicinal plants. XV. Chemical structures of five new resin-glycosides, merremosides a, b, c, d, and e, from the tuber of *Merremia mammosa* (convolvulaceae). Chem. Pharm. Bull..

[26] Ligthelm S.P., Horn D.H.S., Schwartz H.M., Von Holdt M.M. (1954). A chemical study of the fruits of three south african *Ximenia* species, with special reference to the kernel oils. J. Sci. Food Agric..

[27] Bergström S., Erdtman G.A., Rolander B., Stenhagen E., Östling S. (1952). The monoketo-and monohydroxyoctadecanoic acids. Acta Chem. Scand..

[28] Mountanea O.G., Limnios D., Kokotou M.G., Bourboula A., Kokotos G. (2019). Asymmetric synthesis of saturated hydroxy fatty acids and fatty acid esters of hydroxy fatty acids. Eur. J. Org. Chem..

[29] Bourboula A., Limnios D., Kokotou M.G., Mountanea O.G., Kokotos G. (2019). Enantioselective organocatalysis-based synthesis of 3-hydroxy fatty acids and fatty γ-lactones. Molecules.

[30] Yamaguchi M., Hirao I. (1983). An efficient method for the alkynylation of oxiranes using alkynyl boranes. Tetrahedron Lett..

[31] Balas L., Bertrand-Michel J., Viars F., Faugere J., Lefort C., Caspar-Bauguil S., Langin D., Durand T. (2016). Regiocontrolled syntheses of FAHFAs and LC-MS/MS differentiation of regioisomers. Org. Biomol. Chem..

[32] Massalha W., Markovits M., Pichinuk E., Feinstein-Rotkopf Y., Tarshish M., Mishra K., Llado V., Weil M., Escriba P.V., Kakhlon O. (2019). Minerval (2-hydroxyoleic acid) causes cancer cell selective toxicity by uncoupling oxidative phosphorylation and compromising bioenergetic compensation capacity. Biosci. Rep..

[33] Furtek S.L., Backos D.S., Matheson C.J., Reigan P. (2016). Strategies and approaches of targeting STAT3 for cancer treatment. ACS Chem. Biol..

[34] Parolin C., Calonghi N., Presta E., Boga C., Caruana P., Naldi M., Andrisano V., Masotti L., Sartor G. (2012). Mechanism and stereoselectivity of HDAC I inhibition by (*R*)-9-hydroxystearic acid in colon cancer. Biochim. Biophys. Acta.

[35] Terés S., Lladó V., Higuera M., Barceló-Coblijn G., Martin M.L., Noguera-Salvà M.A., Marcilla-Etxenike A., García-Verdugo J.M., Soriano-Navarro M., Saus C. (2012). 2-Hydroxyoleate, a nontoxic membrane binding anticancer drug, induces glioma cell differentiation and autophagy. Proc. Natl. Acad. Sci. USA.

[36] Torgersen M.L., Klokk T.I., Kavaliauskiene S., Klose C., Simons K., Skotland T., Sandvig K. (2016). The anti-tumor drug 2-hydroxyoleic acid (Minerval) stimulates signaling and retrograde transport. Oncotarget.

[37] Martin M.L., Barceló-Coblijn G., de Almeida R.F.M., Noguera-Salvà M.A., Terés S., Higuera M., Liebisch G., Schmitz G., Busquets X., Escribá P.V. (2013). The role of membrane fatty acid remodeling in the antitumor mechanism of action of 2-hydroxyoleic acid. Biochim. Biophys. Acta.

