# Supplementary material for: Asymmetric Synthesis of Saturated and Unsaturated Hydroxy Fatty Acids (HFAs) and Study of Their Antiproliferative Activity
Source: Biomolecules. 2024 Jan 15;14(1):110. doi: 10.3390/biom14010110 (PMC10813182; doi:10.3390/biom14010110)
Supplement: Supplementary file 1 [file biomolecules-14-00110-s001.zip › biomolecules-2752058-supplementary/biomolecules-2752058-supplementary revision and WB/biomolecules-2752058-supplementary revision.pdf]

Asymmetric Synthesis of Saturated and Unsaturated Hydroxy Fatty Acids (HFAs) and Study of Their Antiproliferative Activity

Olga G. Mountanea [1,2], Christiana Mantzourani [1,2], Dimitrios Gkikas [3], Panagiotis K. Politis [3,4], George Kokotos [1,2]*

[1] Department of Chemistry, National and Kapodistrian University of Athens, 15771 Athens, Greece

[2] Center of Excellence for Drug Design and Discovery, National and Kapodistrian University of Athens, 15771 Athens, Greece

[3] Center for Basic Research, Biomedical Research Foundation of the Academy of Athens, 4 Soranou Efesiou Str., 115 27 Athens, Greece

[4] School of Medicine, European University Cyprus, Nicosia 2404, Cyprus

*To whom correspondence should be addressed. For G.K.: phone: +30 210 7274462; fax: +30 210 7274761; E-mail: gkokotos@chem.uoa.gr.

SUPPLEMENTARY MATERIALS

	**Page**
**Table S1.** 2-way ANOVA statistical analysis	**S3**
**NMR Spectra**	**S4**

Table S1. 2-way ANOVA statistical analysis with multiple comparisons to DMSO, for all MTT tests of HFAs in A549 and SF268 human cancer cell lines ($p<0.05$ *, $p<0.01$ **, $p<0.001$ ***, $p<0.0001$ ****).

**A549**	10 μM	25 μM	35 μM	50 μM	75 μM	100 μM
6SHSA	ns	**	****	****	****	****
6RHSA	ns	ns	****	****	****	****
6SHPA	ns	****	****	****	****	****
6RHPA	ns	ns	ns	ns	****	****
7SHMA	ns	ns	ns	ns	****	****
7RHMA	ns	ns	ns	***	****	****
8SHPA	ns	**	****	****	****	****
8RHPA	ns	***	****	****	****	****
8SHSA	ns	ns	**	****	****	****
8RHSA	ns	ns	ns	****	****	****
11SHPA	ns	ns	ns	ns	ns	ns
11RHPA	ns	ns	ns	ns	ns	ns
11SHSA	ns	ns	ns	ns	****	****
11RHSA	ns	ns	ns	ns	****	****
7SHOA	ns	ns	ns	ns	****	****
7SHPOA	ns	ns	ns	ns	**	****
7RHSA	*	**	****	****	****	****
PA	-	ns	-	ns	-	ns
SA	-	ns	-	ns	-	ns

**SF268**	10 μM	25 μM	35 μM	50 μM	75 μM	100 μM
6SHPA	ns	ns	*	****	****	****
6RHPA	ns	ns	ns	ns	ns	ns
6SHSA	ns	ns	ns	ns	****	****
6RHSA	ns	ns	ns	ns	ns	***
8SHPA	ns	ns	ns	***	****	****
8RHPA	ns	ns	ns	****	****	****
8SHSA	ns	ns	ns	ns	*	***
8RHSA	ns	ns	*	ns	****	****
7RHSA	ns	****	-	****	-	****
PA	-	ns	-	ns	-	ns
SA	-	ns	-	ns	-	*

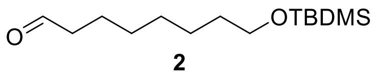

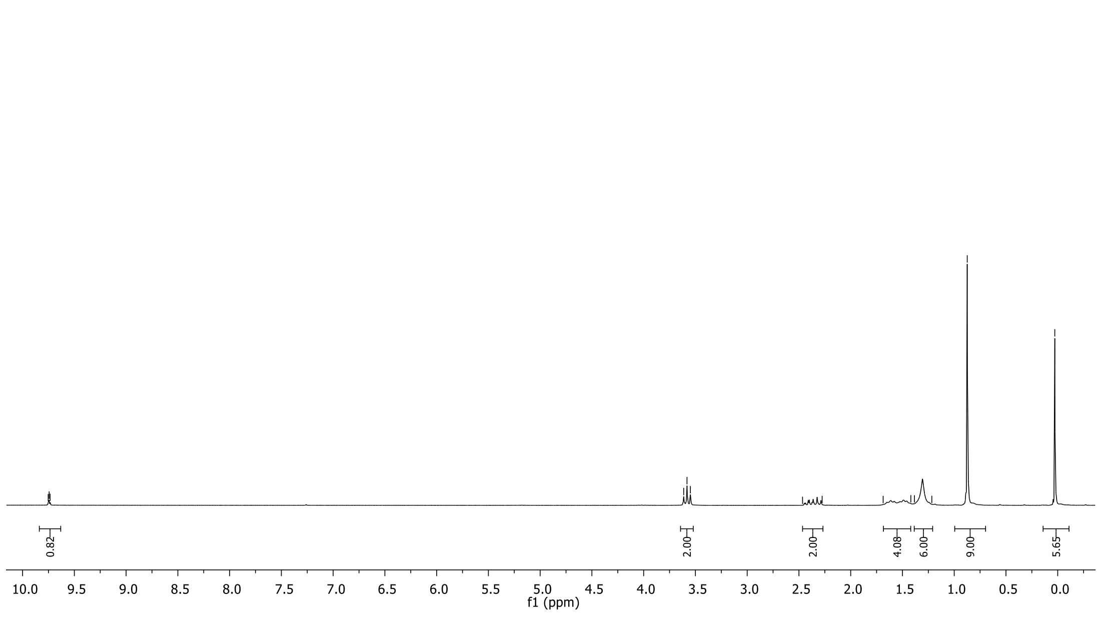
9.75
9.74
9.73
3.61
3.58
3.55
2.46
2.28
1.69
1.42
1.38
1.22
0.87
0.03
0.82
2.00
2.00
4.08
6.00
9.00
5.65
10.0 9.5 9.0 8.5 8.0 7.5 7.0 6.5 6.0 5.5 5.0 4.5 4.0 3.5 3.0 2.5 2.0 1.5 1.0 0.5 0.0
f1 (ppm)

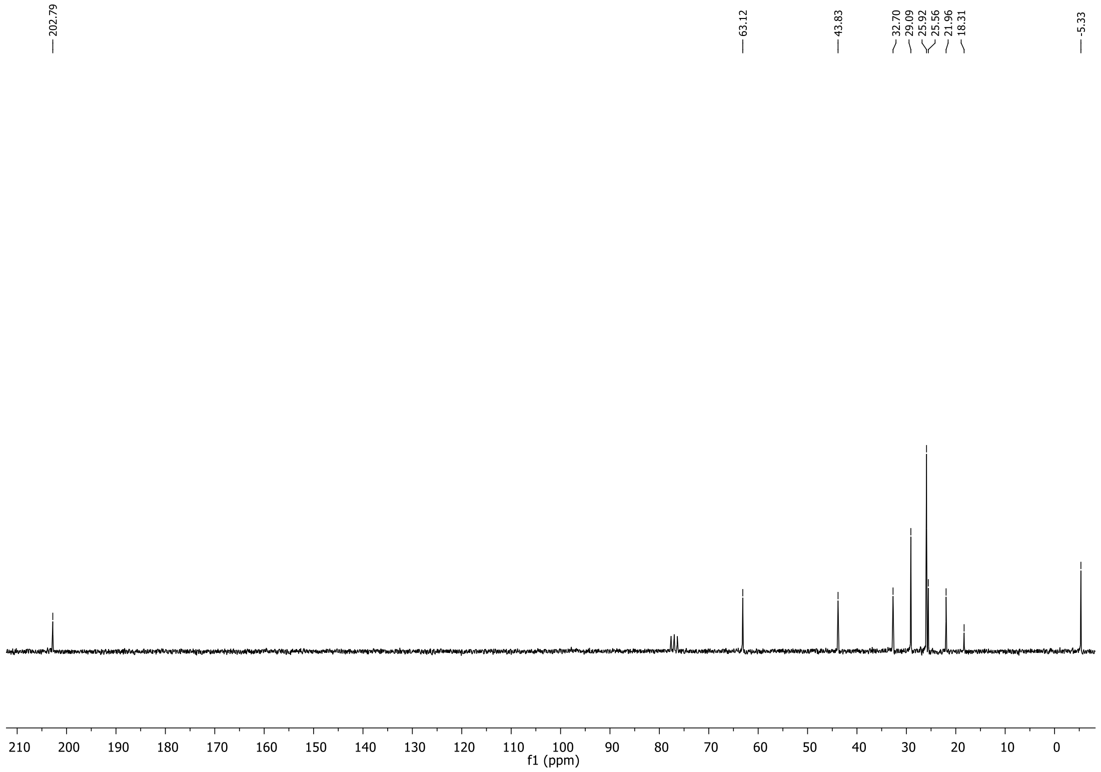
202.79
63.12
43.83
32.70
29.09
25.92
25.56
21.96
18.31
-5.33
210 200 190 180 170 160 150 140 130 120 110 100 90 80 70 60 50 40 30 20 10 0
f1 (ppm)

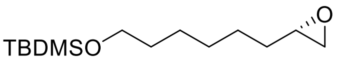

3

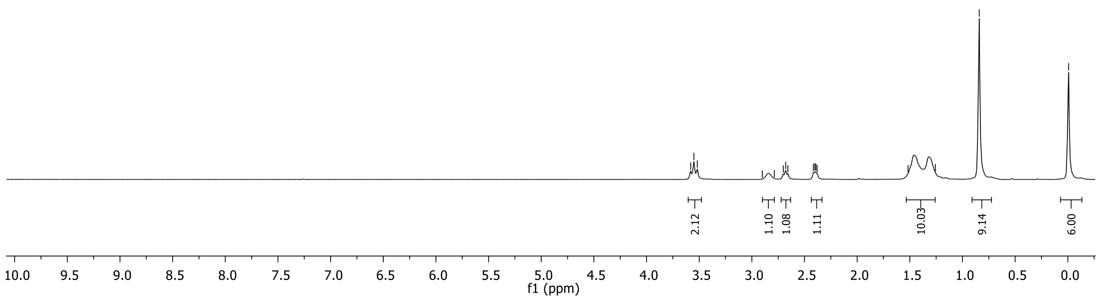

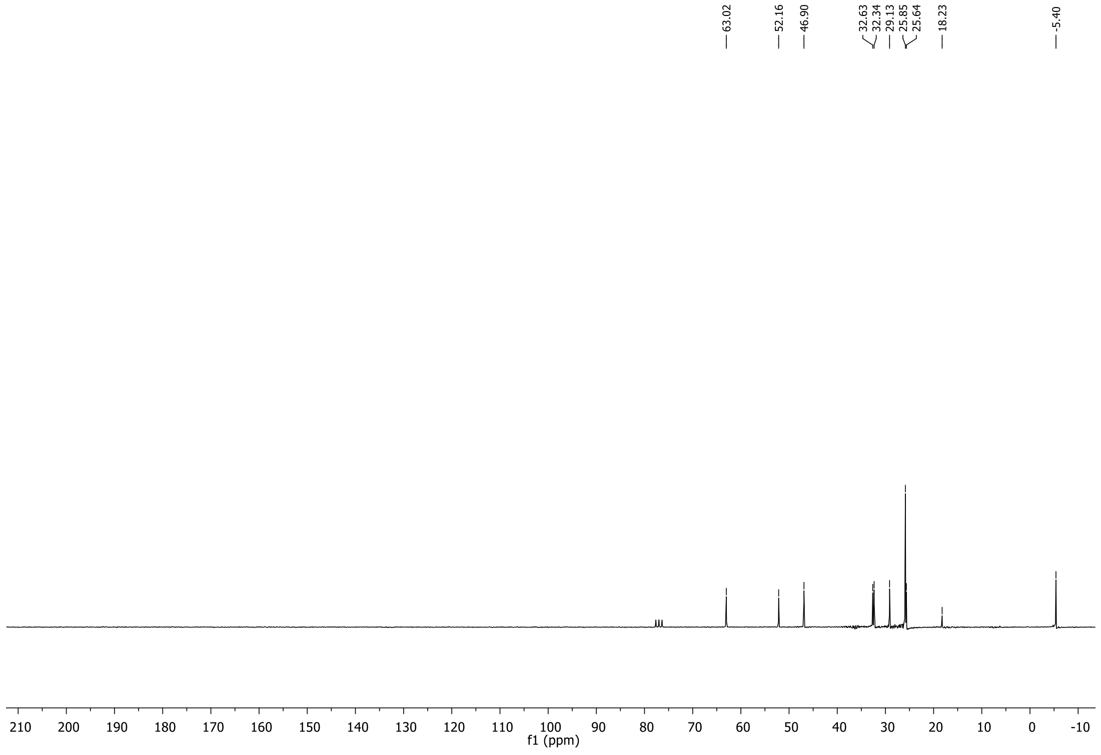

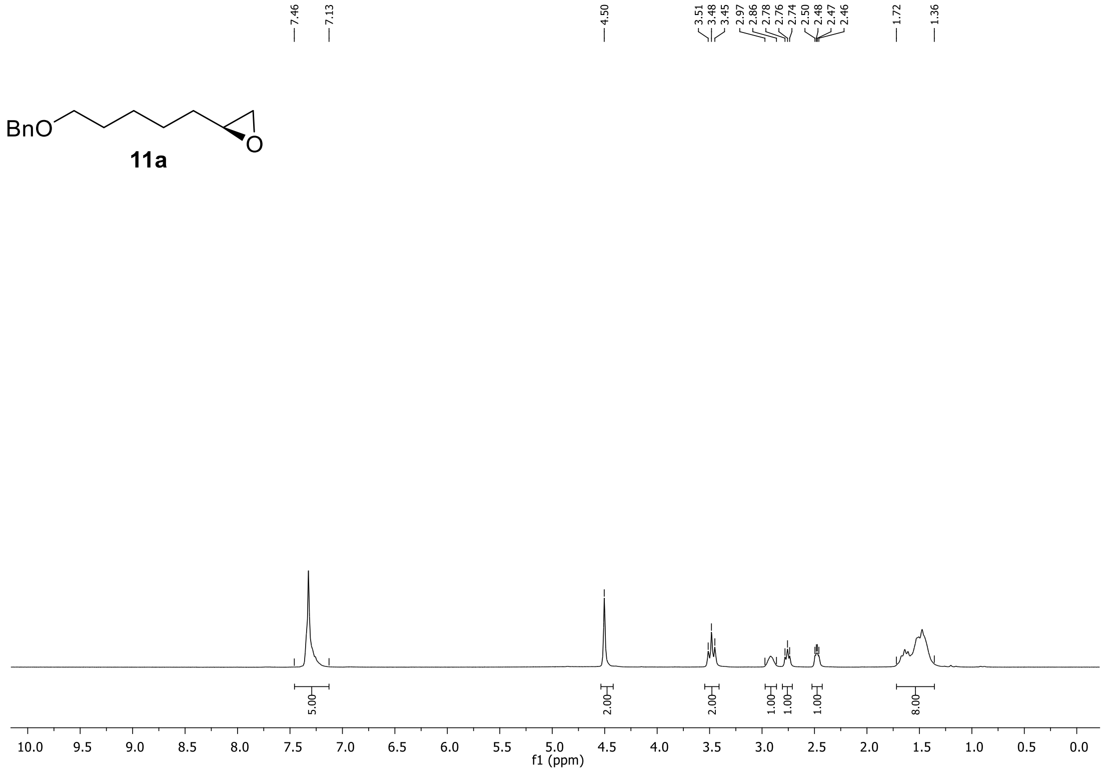
BnO
O
7.46
7.13
4.50
3.51
3.48
3.45
2.97
2.86
2.78
2.76
2.74
2.50
2.48
2.47
2.46
1.72
1.36
5.00
2.00
2.00
1.00
1.00
1.00
8.00
f1 (ppm)

11a

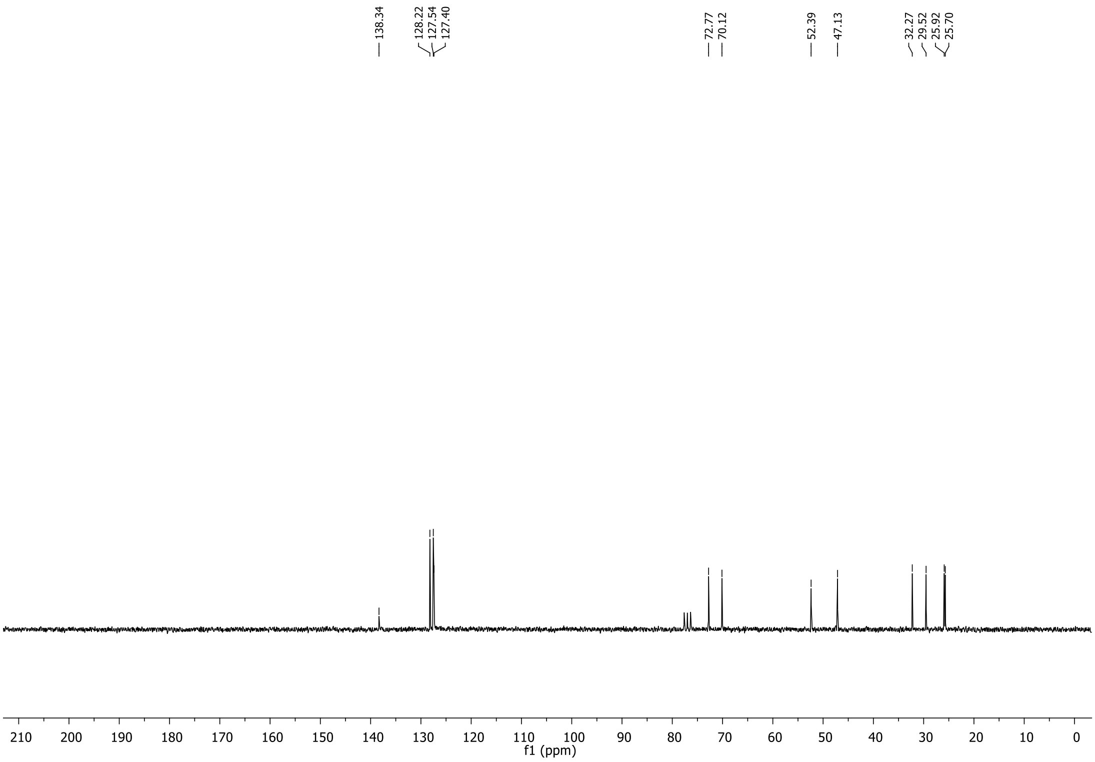
138.34
128.22
127.54
127.40
72.77
70.12
52.39
47.13
32.27
29.52
25.92
25.70
f1 (ppm)

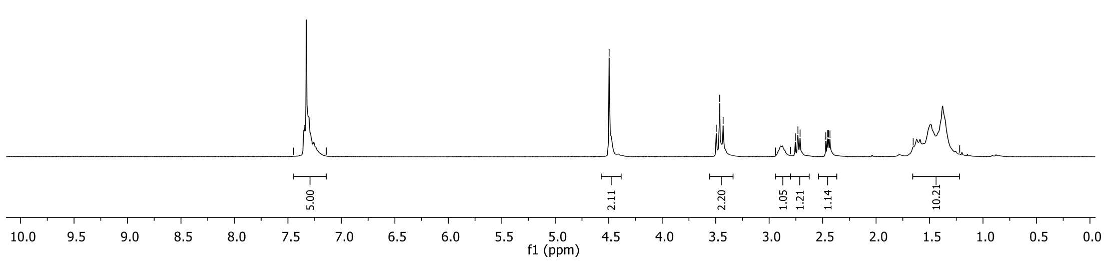
7.45
7.14
4.50
3.49
3.46
3.43
2.94
2.80
2.76
2.73
2.71
2.47
2.46
2.44
2.43
1.65
1.22
5.00
2.11
2.20
1.05
1.21
1.14
10.21
f1 (ppm)

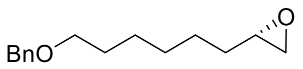
BnO
O

11b

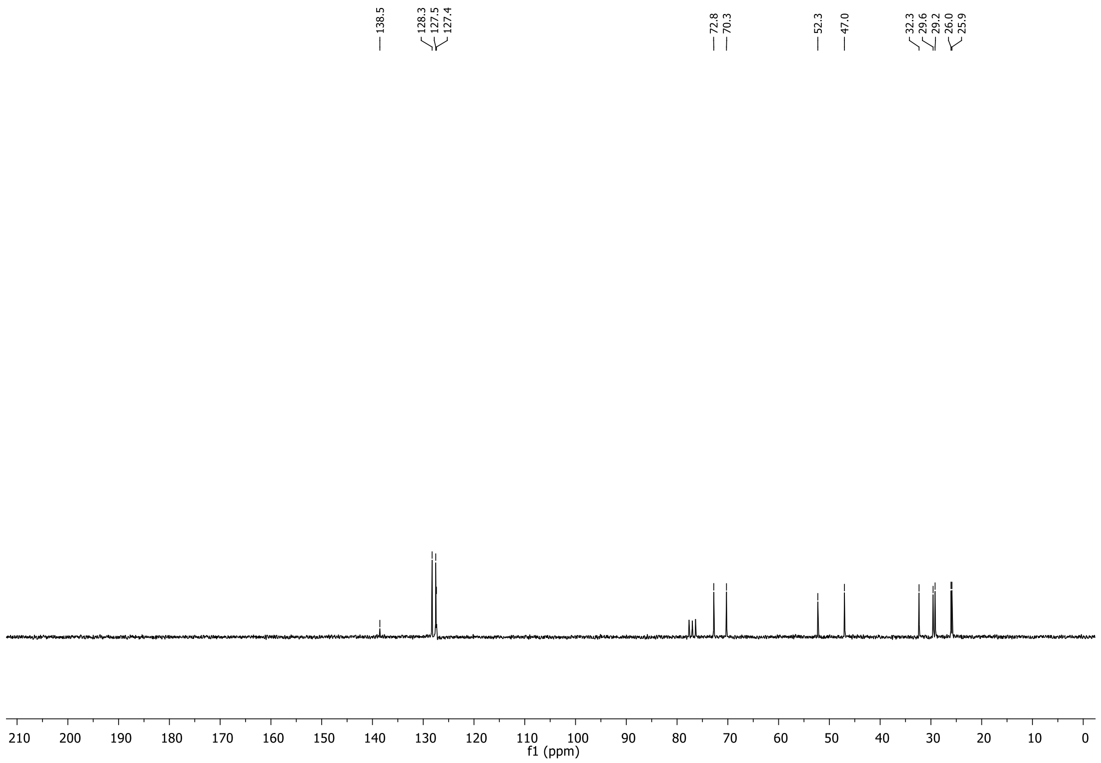
138.5
128.3
127.5
127.4
72.8
70.3
52.3
47.0
32.3
29.6
29.2
26.0
25.9
f1 (ppm)

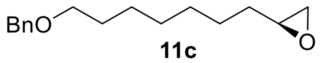
BnO
O

11c

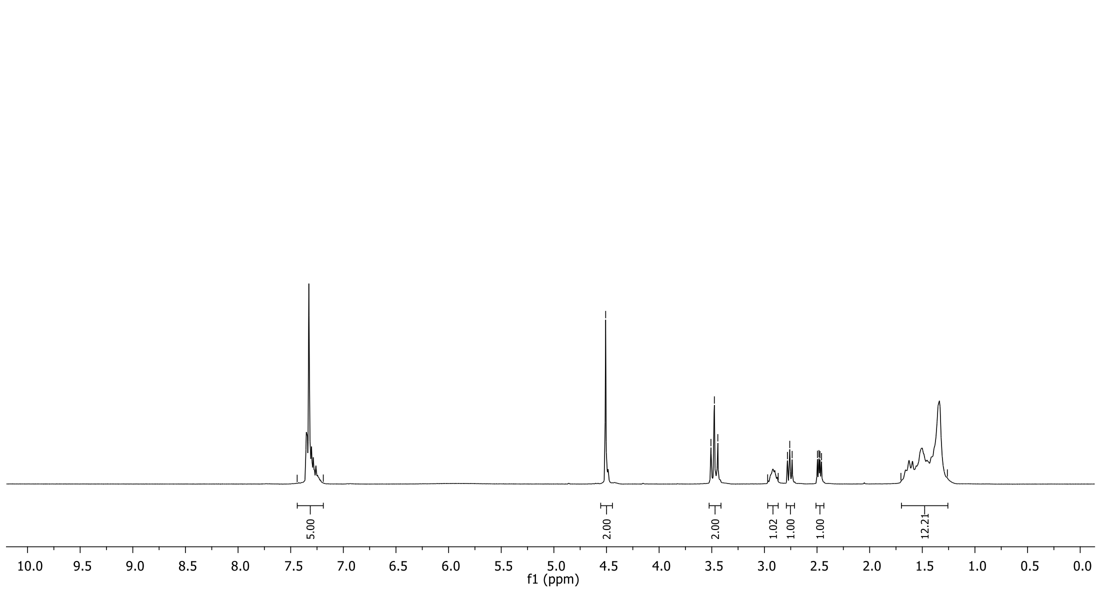
7.44
7.19
4.51
3.51
3.48
3.44
2.97
2.87
2.78
2.76
2.74
2.50
2.48
2.47
2.46
1.70
1.26
5.00
2.00
2.00
1.02
1.00
1.00
12.21
10.0 9.5 9.0 8.5 8.0 7.5 7.0 6.5 6.0 5.5 5.0 4.5 4.0 3.5 3.0 2.5 2.0 1.5 1.0 0.5 0.0
f1 (ppm)

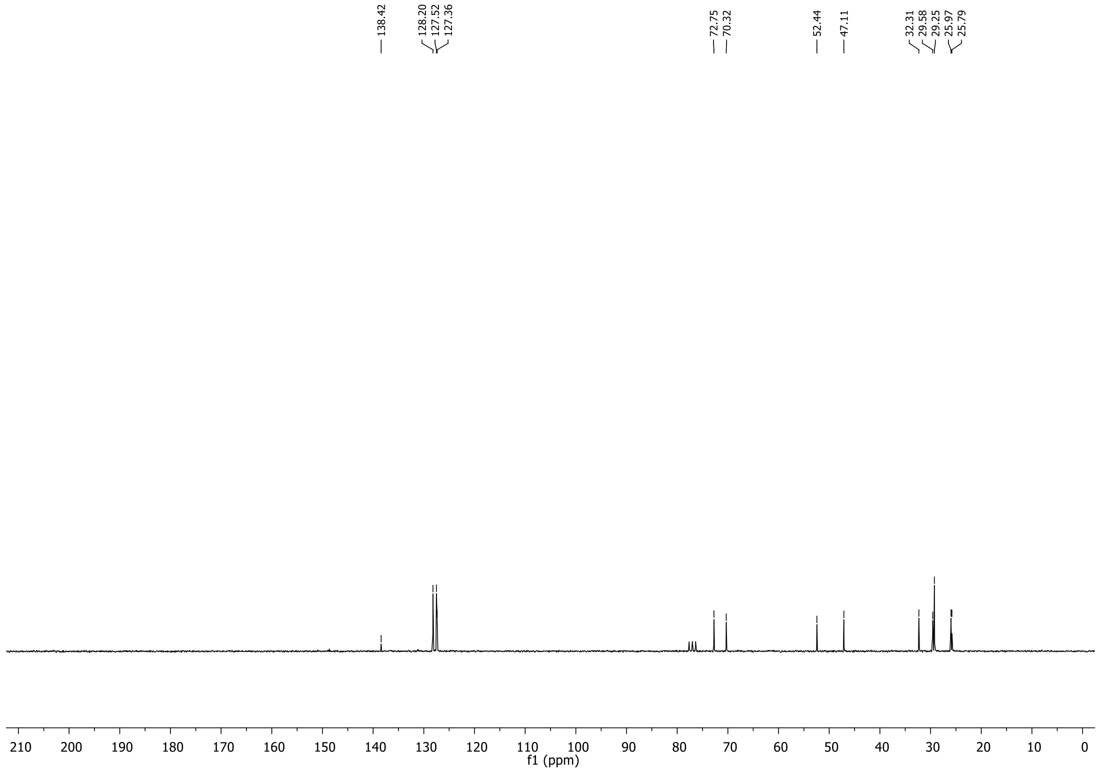
138.42
128.20
127.52
127.36
72.75
70.32
52.44
47.11
32.31
29.58
29.25
25.97
25.79
210 200 190 180 170 160 150 140 130 120 110 100 90 80 70 60 50 40 30 20 10 0
f1 (ppm)

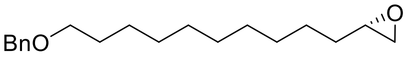
BnO
O

11d

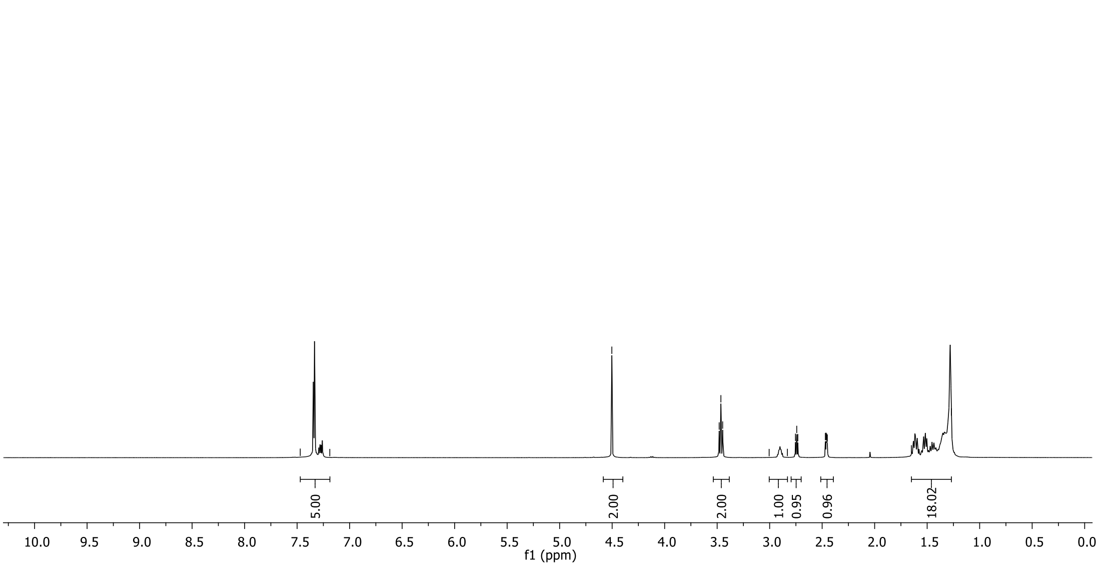
7.47
7.19
4.50
3.48
3.46
3.45
3.00
2.83
2.75
2.74
2.73
2.47
2.46
2.46
2.45
1.65
1.27
5.00
2.00
2.00
1.00
0.95
0.96
18.02
10.0 9.5 9.0 8.5 8.0 7.5 7.0 6.5 6.0 5.5 5.0 4.5 4.0 3.5 3.0 2.5 2.0 1.5 1.0 0.5 0.0
f1 (ppm)

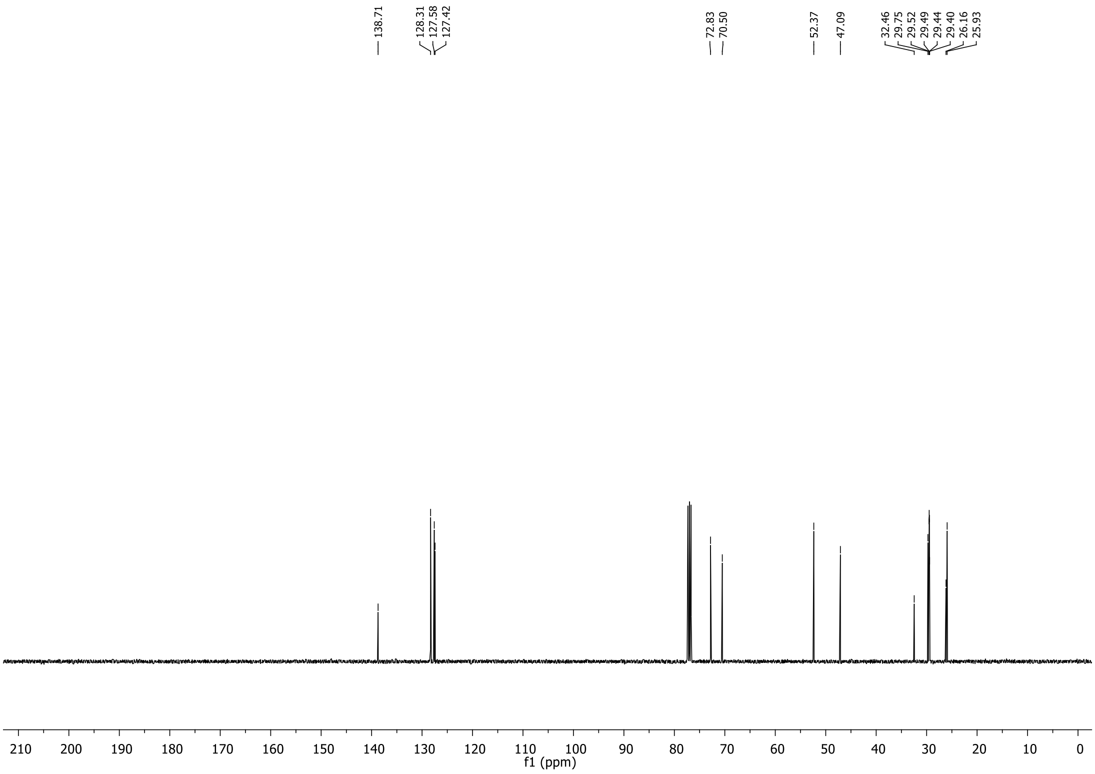
138.71
128.31
127.58
127.42
72.83
70.50
52.37
47.09
32.46
29.75
29.52
29.49
29.44
29.40
26.16
25.93
210 200 190 180 170 160 150 140 130 120 110 100 90 80 70 60 50 40 30 20 10 0
f1 (ppm)

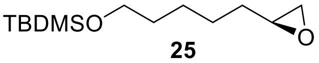
TBDMSO
O

25

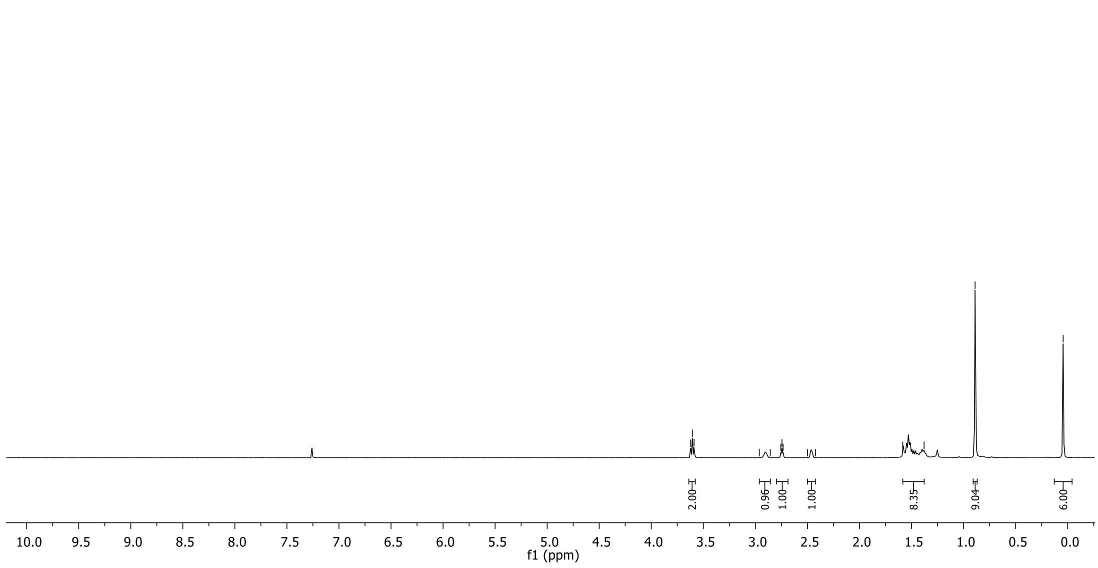
3.62
3.61
3.59
2.96
2.86
2.76
2.74
2.73
2.50
2.42
1.58
1.38
0.89
0.04
2.00
0.96
1.00
1.00
8.35
9.04
6.00
10.0 9.5 9.0 8.5 8.0 7.5 7.0 6.5 6.0 5.5 5.0 4.5 4.0 3.5 3.0 2.5 2.0 1.5 1.0 0.5 0.0
f1 (ppm)

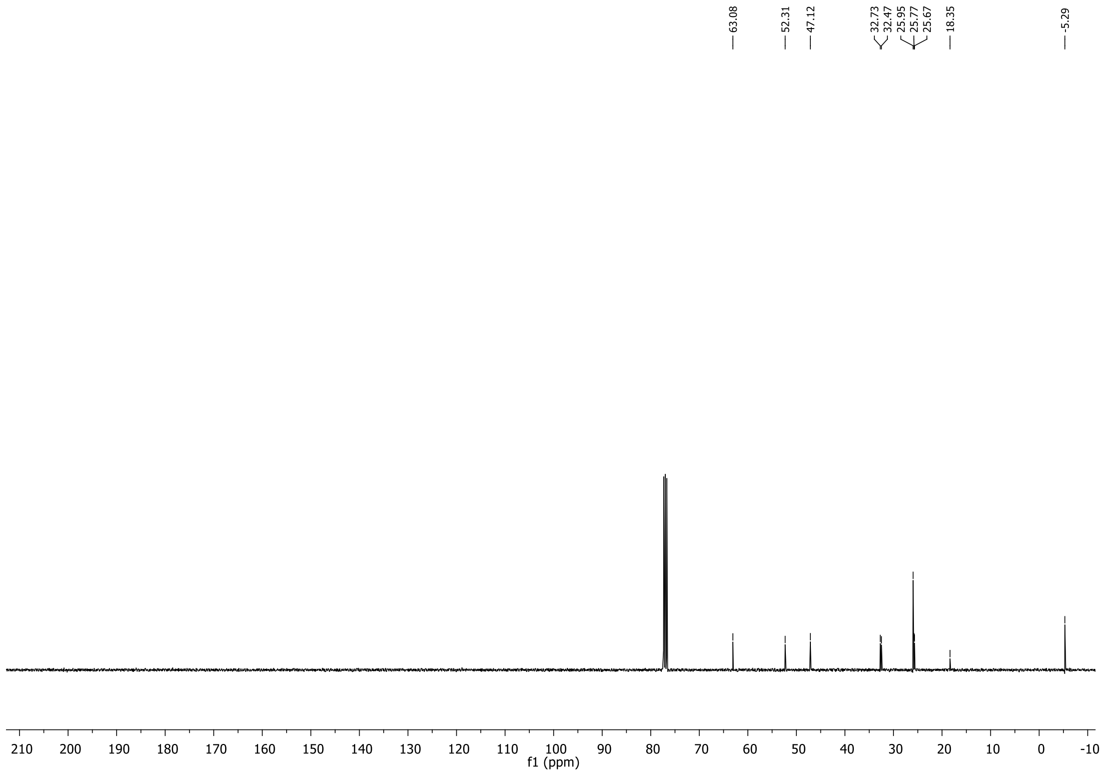
63.08
52.31
47.12
32.73
32.47
25.95
25.77
25.67
18.35
-5.29
210 200 190 180 170 160 150 140 130 120 110 100 90 80 70 60 50 40 30 20 10 0 -10
f1 (ppm)

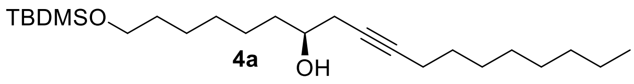
TBDMSO
OH

4a

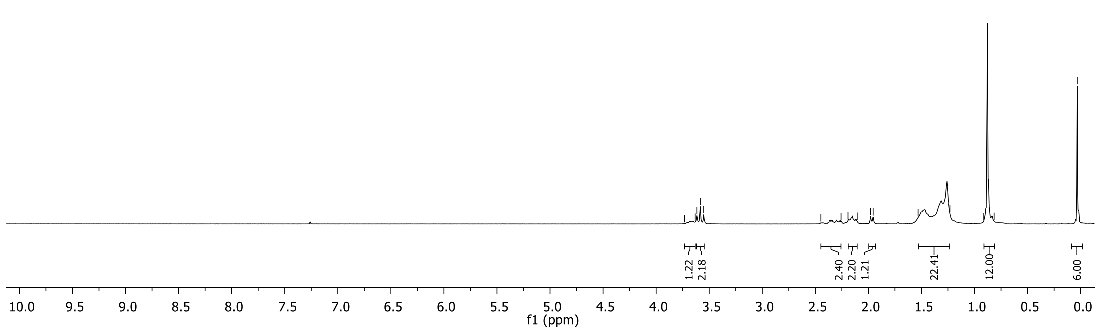
3.73
3.63
3.62
3.58
3.55
2.45
2.26
2.19
2.11
1.98
1.96
1.53
1.23
0.91
0.82
0.03
1.22
2.18
2.40
2.20
1.21
22.41
12.00
6.00
10.0 9.5 9.0 8.5 8.0 7.5 7.0 6.5 6.0 5.5 5.0 4.5 4.0 3.5 3.0 2.5 2.0 1.5 1.0 0.5 0.0
f1 (ppm)

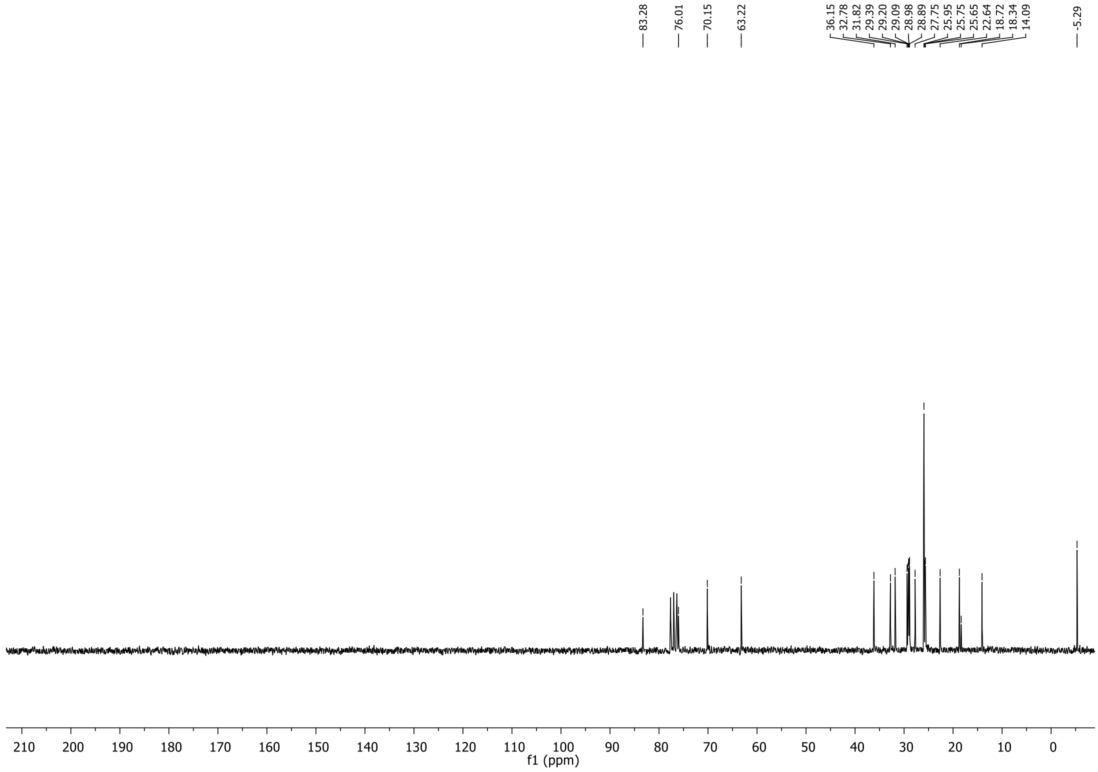
83.28
76.01
70.15
63.22
36.15
32.78
31.82
29.39
29.20
29.09
28.98
28.89
27.75
25.95
25.75
25.65
22.64
18.72
18.34
14.09
-5.29
210 200 190 180 170 160 150 140 130 120 110 100 90 80 70 60 50 40 30 20 10 0
f1 (ppm)

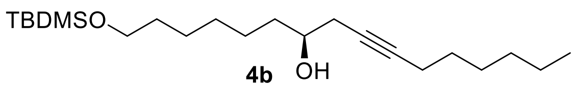
TBDMSO
OH
4b

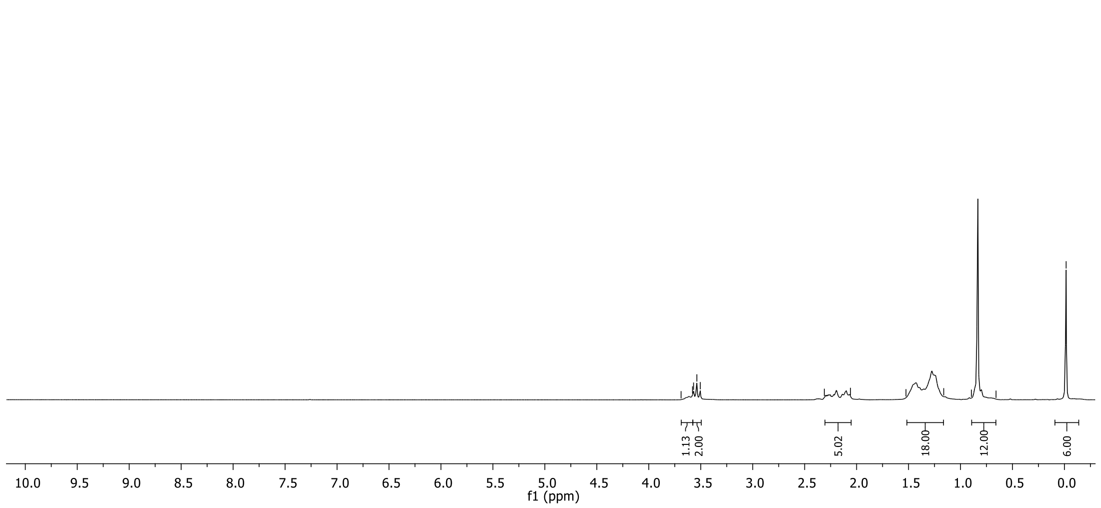
3.69
3.58
3.57
3.54
3.50
2.31
2.06
1.52
1.16
0.90
0.66
-0.02
1.13
2.00
5.02
18.00
12.00
6.00
10.0 9.5 9.0 8.5 8.0 7.5 7.0 6.5 6.0 5.5 5.0 4.5 4.0 3.5 3.0 2.5 2.0 1.5 1.0 0.5 0.0
f1 (ppm)

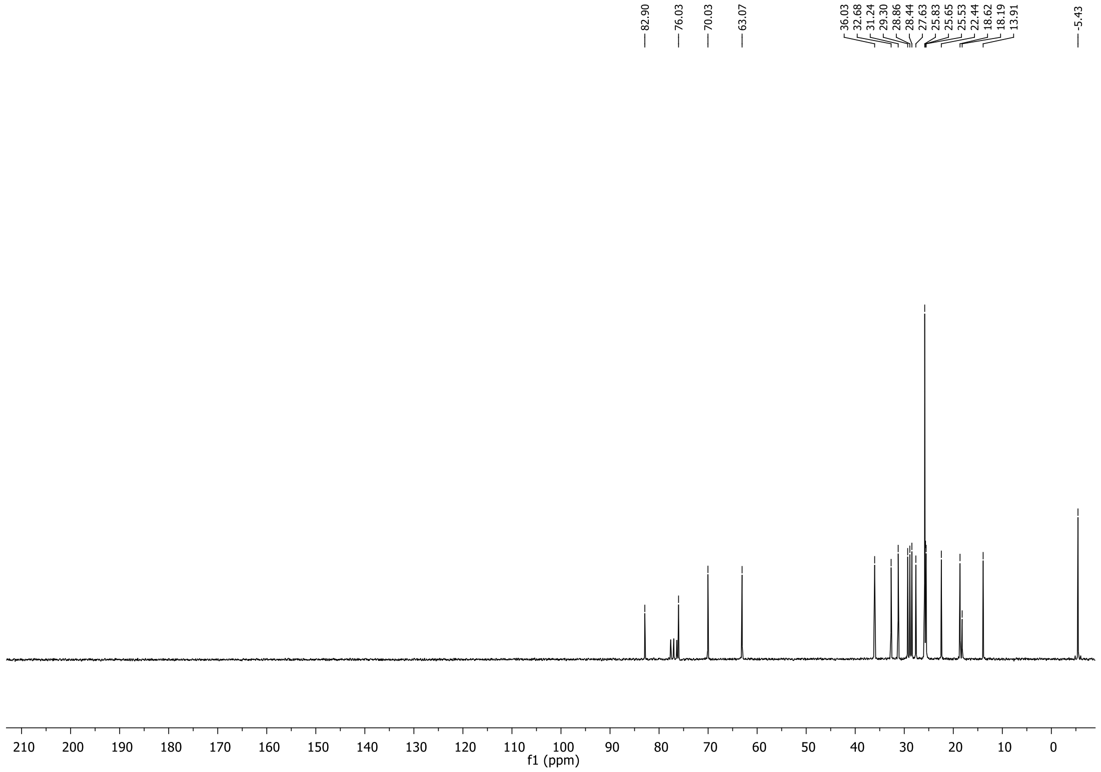
82.90
76.03
70.03
63.07
36.03
32.68
31.24
29.30
28.86
28.44
27.63
25.83
25.65
25.53
22.44
18.62
18.19
13.91
-5.43
210 200 190 180 170 160 150 140 130 120 110 100 90 80 70 60 50 40 30 20 10 0
f1 (ppm)

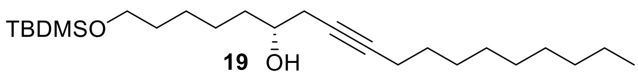
TBDMSO
OH

19

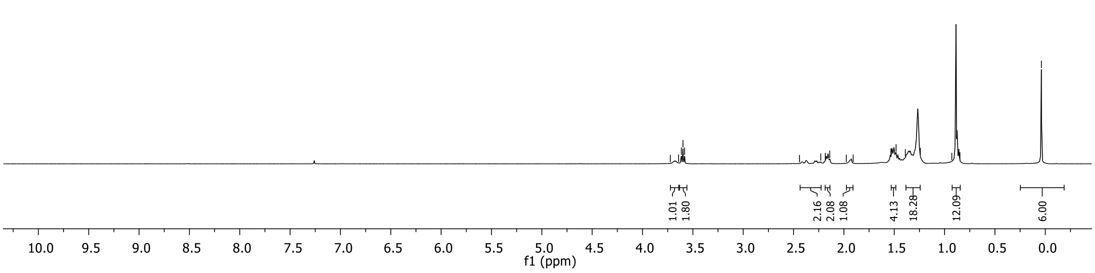
3.72
3.64
3.61
3.60
3.58
2.44
2.23
2.19
2.14
1.98
1.91
1.53
1.48
1.39
1.24
0.93
0.85
0.04
1.01
1.80
2.16
2.08
1.08
4.13
18.28
12.09
6.00
f1 (ppm)

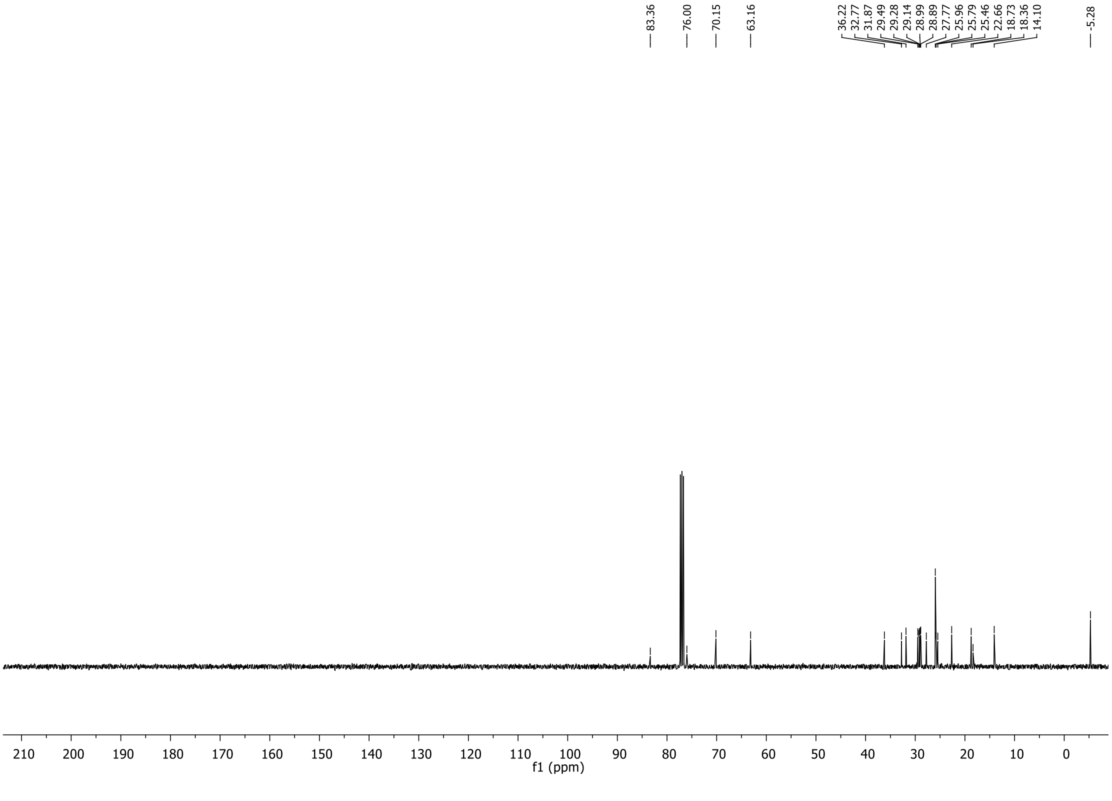
83.36
76.00
70.15
63.16
36.22
32.77
31.87
29.49
29.28
29.14
28.99
28.89
27.77
25.96
25.79
25.46
22.66
18.73
18.36
14.10
-5.28
f1 (ppm)

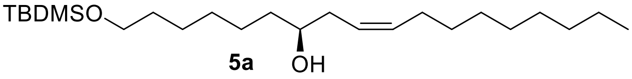
TBDMSO
OH

5a

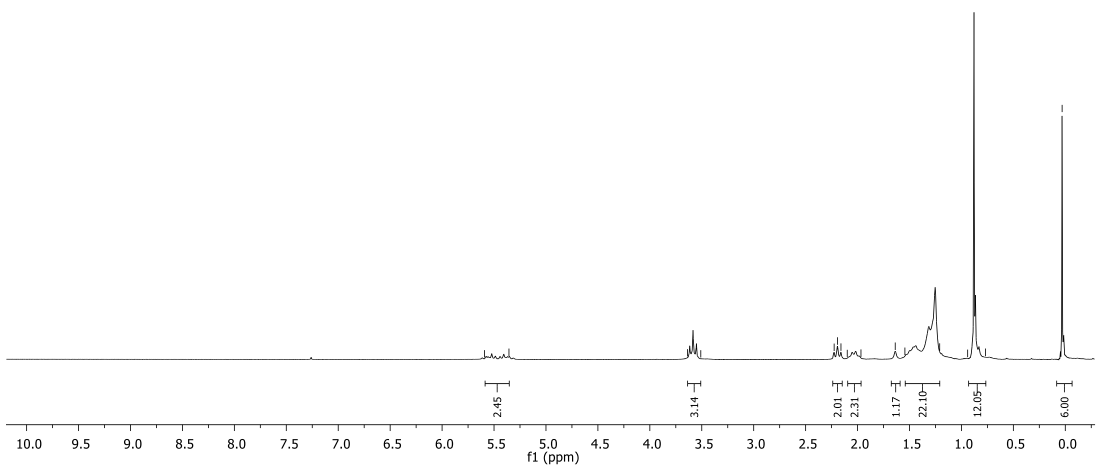
5.59
5.36
3.64
3.51
2.23
2.19
2.16
2.10
1.97
1.64
1.54
1.21
0.94
0.77
0.03
2.45
3.14
2.01
2.31
1.17
22.10
12.05
6.00
f1 (ppm)

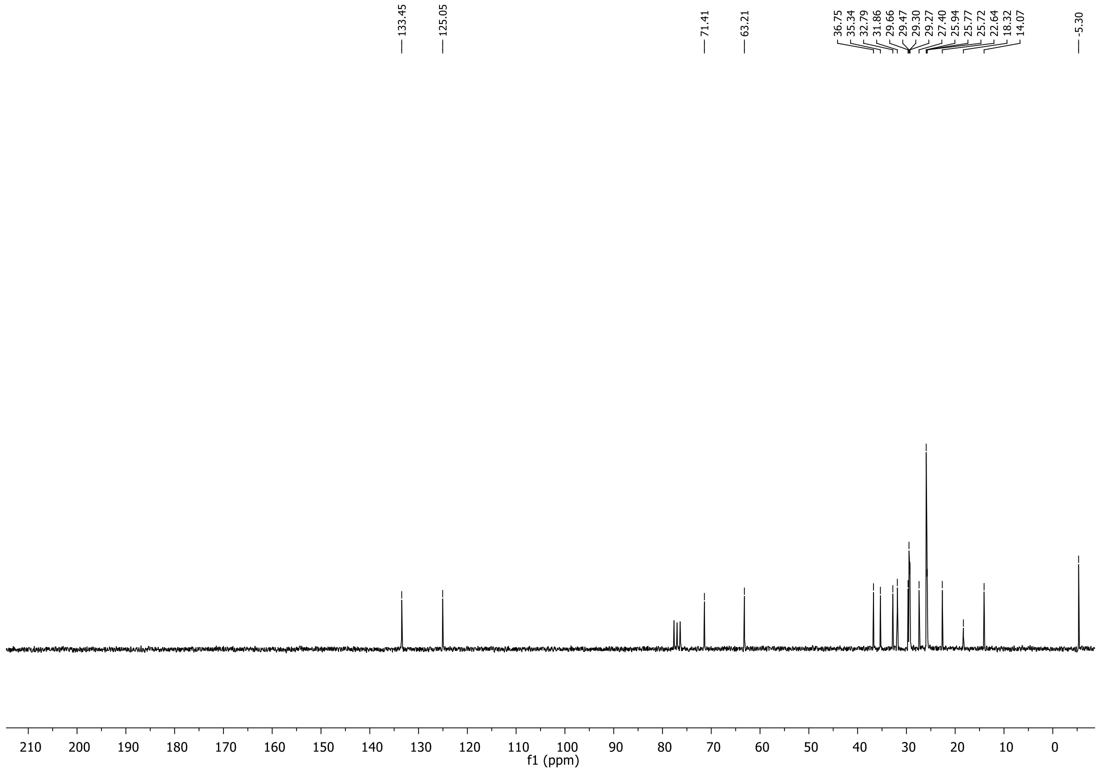
133.45
125.05
71.41
63.21
36.75
35.34
32.79
31.86
29.66
29.47
29.30
29.27
27.40
25.94
25.77
25.72
22.64
18.32
14.07
-5.30
f1 (ppm)

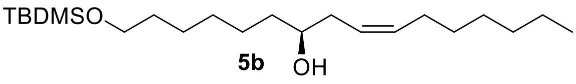
TBDMSO
OH

5b

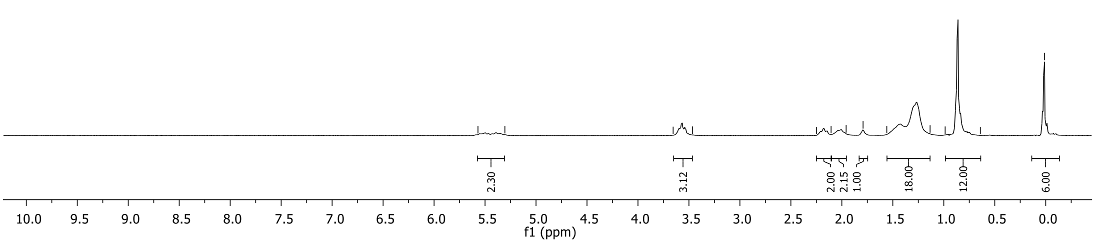
5.57
5.31
3.65
3.46
2.25
2.11
1.96
1.79
1.56
1.13
0.99
0.64
0.01
2.30
3.12
2.00
2.15
1.00
18.00
12.00
6.00
10.0 9.5 9.0 8.5 8.0 7.5 7.0 6.5 6.0 5.5 5.0 4.5 4.0 3.5 3.0 2.5 2.0 1.5 1.0 0.5 0.0
f1 (ppm)

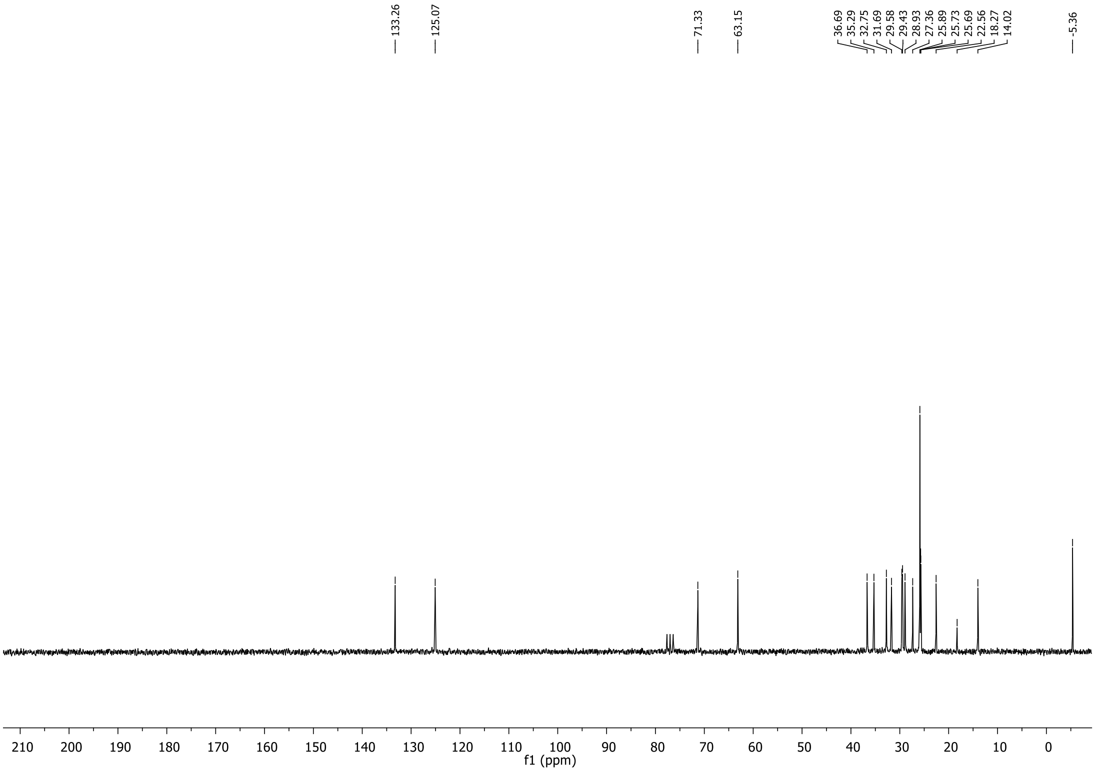
133.26
125.07
71.33
63.15
36.69
35.29
32.75
31.69
29.58
29.43
28.93
27.36
25.89
25.73
25.69
22.56
18.27
14.02
-5.36
210 200 190 180 170 160 150 140 130 120 110 100 90 80 70 60 50 40 30 20 10 0
f1 (ppm)

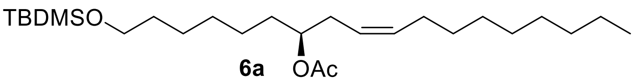
TBDMSO
OAc

6a

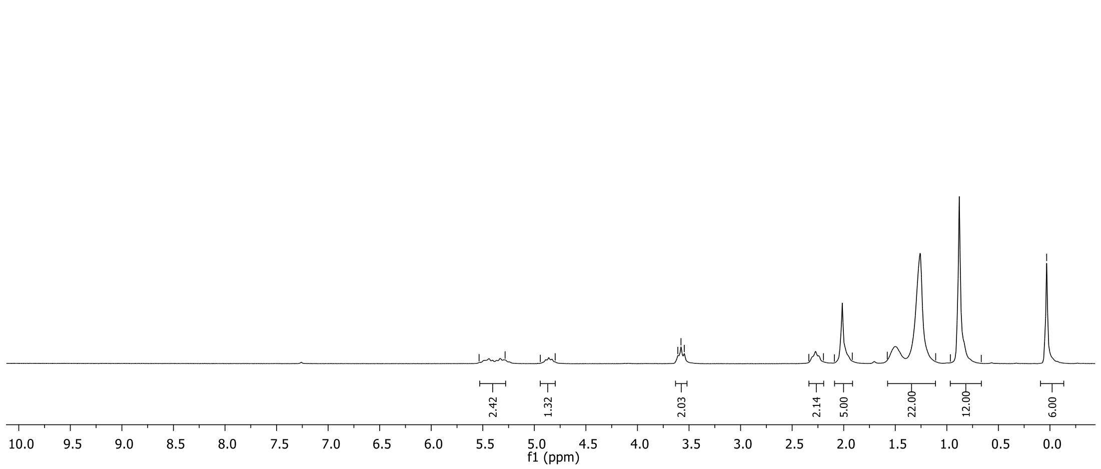
5.54
5.28
4.94
4.80
3.61
3.58
3.55
2.34
2.20
2.09
1.92
1.58
1.11
0.97
0.67
0.03
2.42
1.32
2.03
2.14
5.00
22.00
12.00
6.00
10.0 9.5 9.0 8.5 8.0 7.5 7.0 6.5 6.0 5.5 5.0 4.5 4.0 3.5 3.0 2.5 2.0 1.5 1.0 0.5 0.0
f1 (ppm)

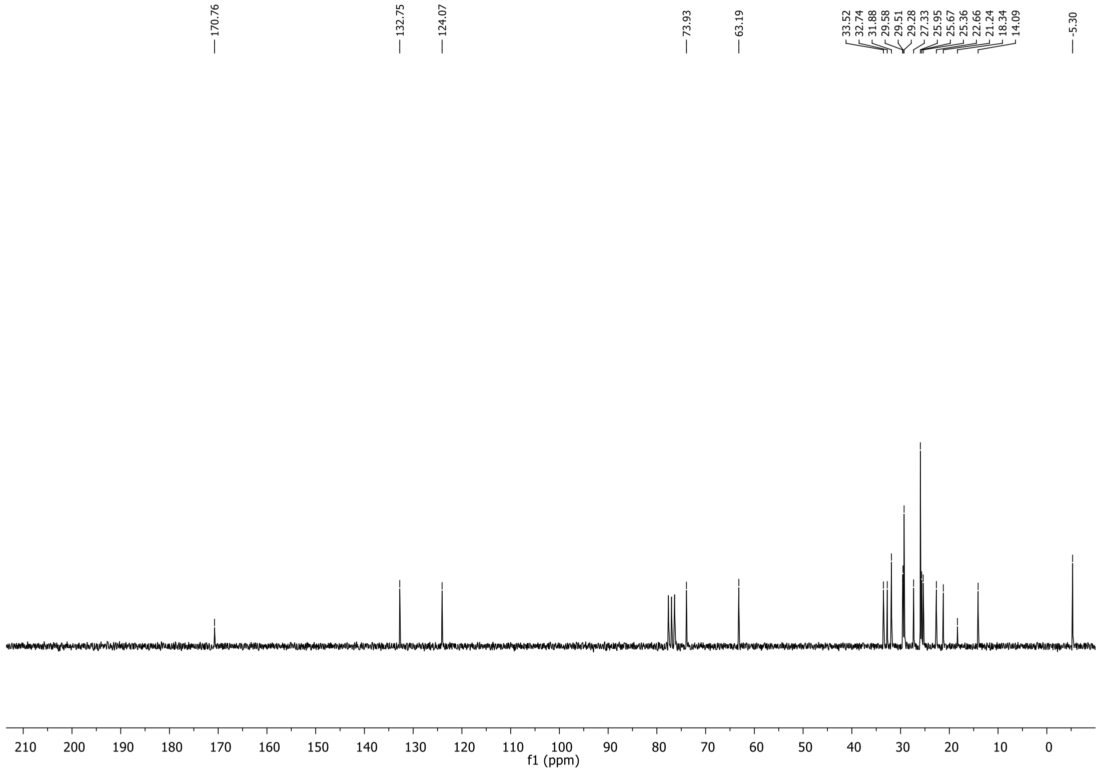
170.76
132.75
124.07
73.93
63.19
33.52
32.74
31.88
29.58
29.51
29.28
27.33
25.95
25.67
25.36
22.66
21.24
18.34
14.09
-5.30
210 200 190 180 170 160 150 140 130 120 110 100 90 80 70 60 50 40 30 20 10 0
f1 (ppm)

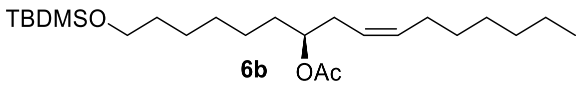
TBDMSO
OAc

6b

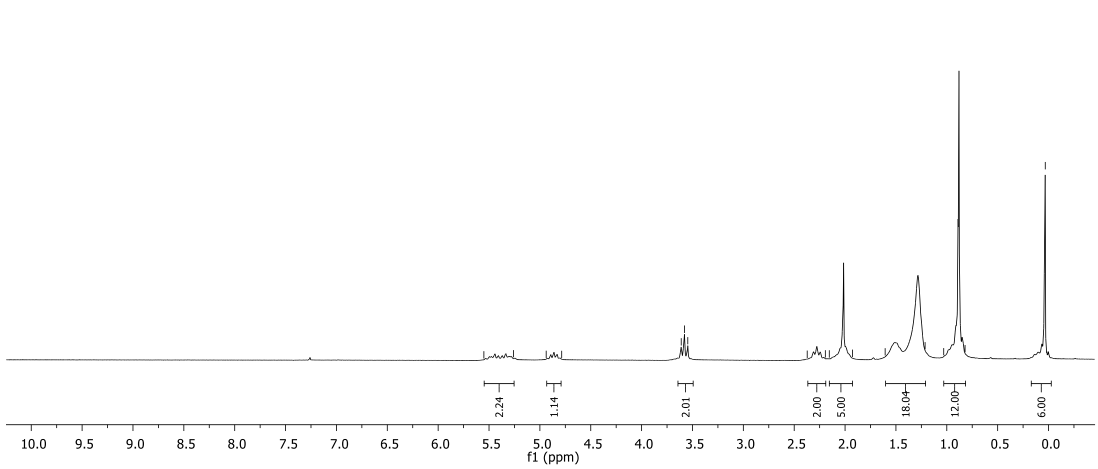
5.55
5.26
4.94
4.79
3.61
3.58
3.55
2.37
2.19
2.16
1.93
1.61
1.21
1.03
0.82
0.03
2.24
1.14
2.01
2.00
5.00
18.04
12.00
6.00
10.0 9.5 9.0 8.5 8.0 7.5 7.0 6.5 6.0 5.5 5.0 4.5 4.0 3.5 3.0 2.5 2.0 1.5 1.0 0.5 0.0
f1 (ppm)

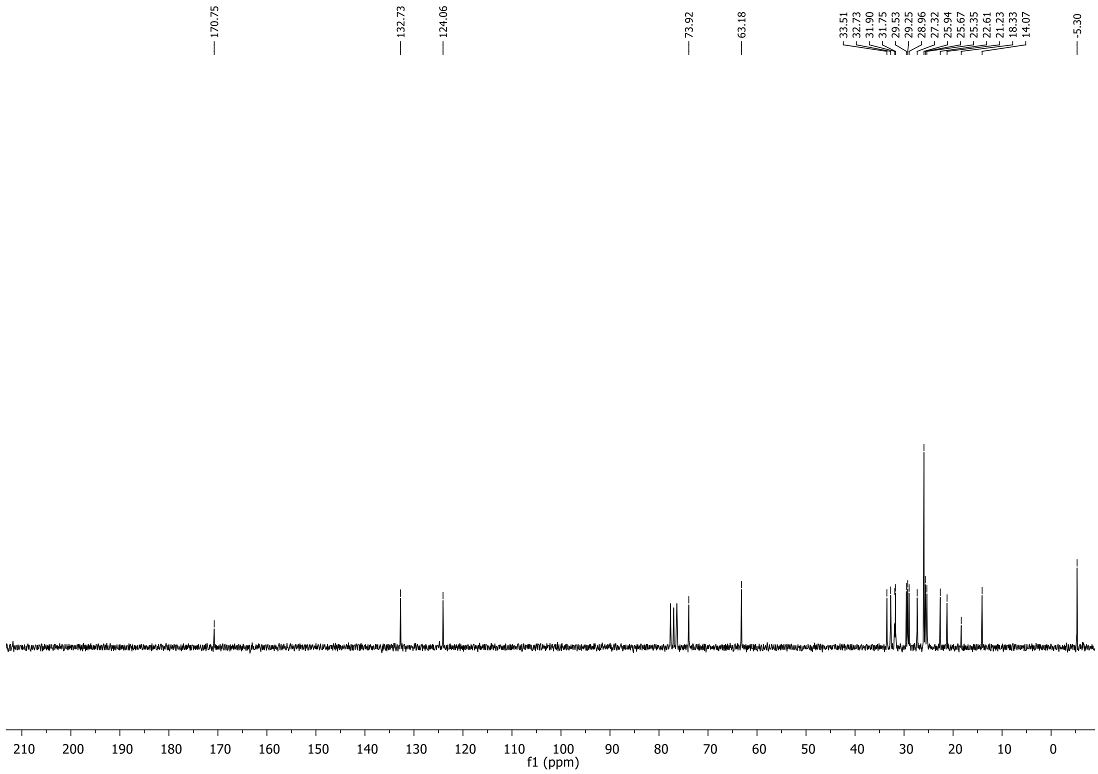
170.75
132.73
124.06
73.92
63.18
33.51
32.73
31.90
31.75
29.53
29.25
28.96
27.32
25.94
25.67
25.35
22.61
21.23
18.33
14.07
-5.30
210 200 190 180 170 160 150 140 130 120 110 100 90 80 70 60 50 40 30 20 10 0
f1 (ppm)

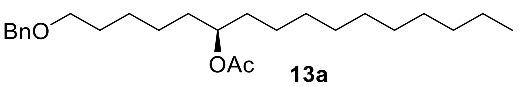
BnO
OAc

13a

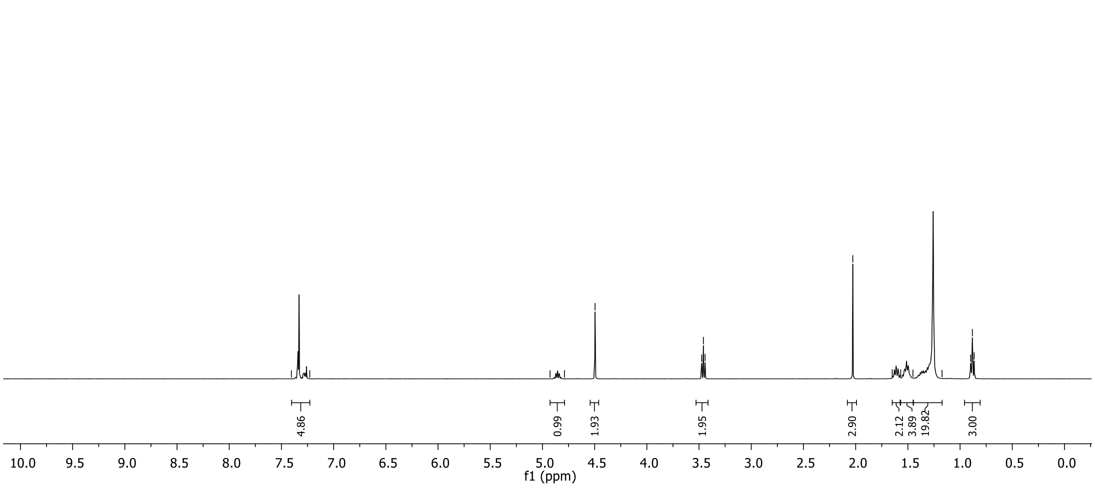
7.40
7.23
4.93
4.79
4.50
3.47
3.46
3.44
2.03
1.65
1.57
1.45
1.17
0.90
0.88
0.86
4.86
0.99
1.93
1.95
2.90
2.12
3.89
19.82
3.00
10.0 9.5 9.0 8.5 8.0 7.5 7.0 6.5 6.0 5.5 5.0 4.5 4.0 3.5 3.0 2.5 2.0 1.5 1.0 0.5 0.0
f1 (ppm)

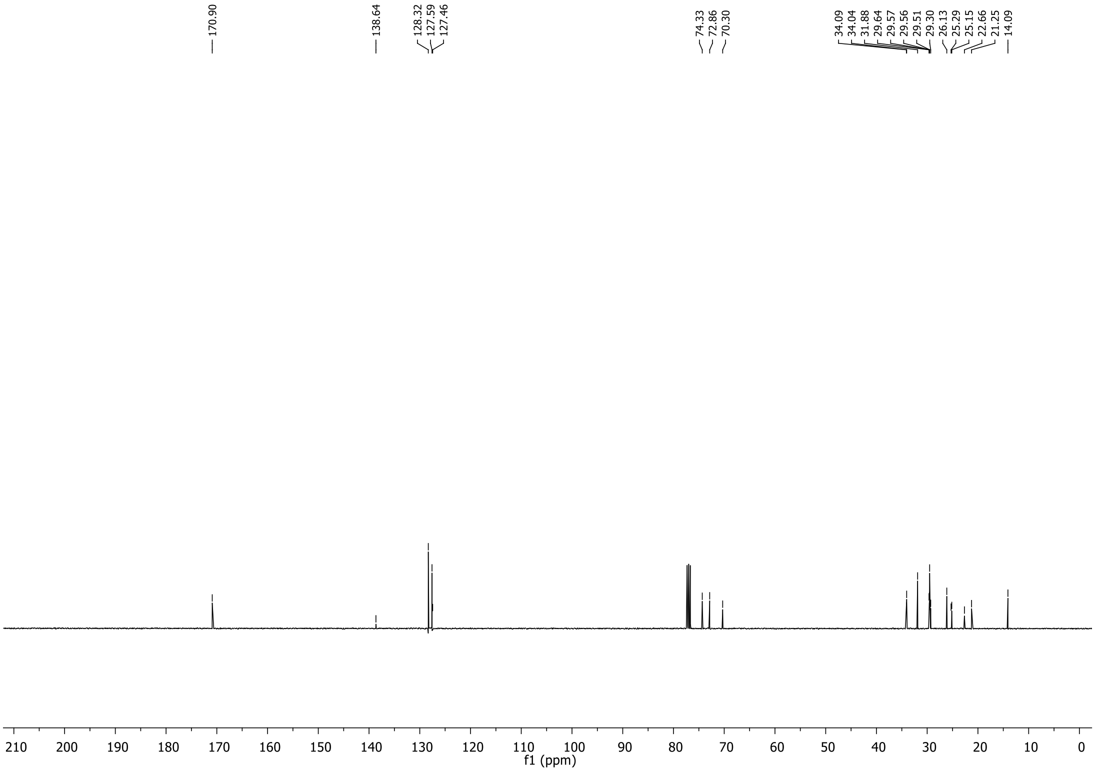
170.90
138.64
128.32
127.59
127.46
74.33
72.86
70.30
34.09
34.04
31.88
29.64
29.57
29.56
29.51
29.30
26.13
25.29
25.15
22.66
21.25
14.09
210 200 190 180 170 160 150 140 130 120 110 100 90 80 70 60 50 40 30 20 10 0
f1 (ppm)

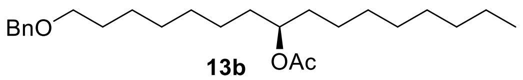
BnO
OAc

13b

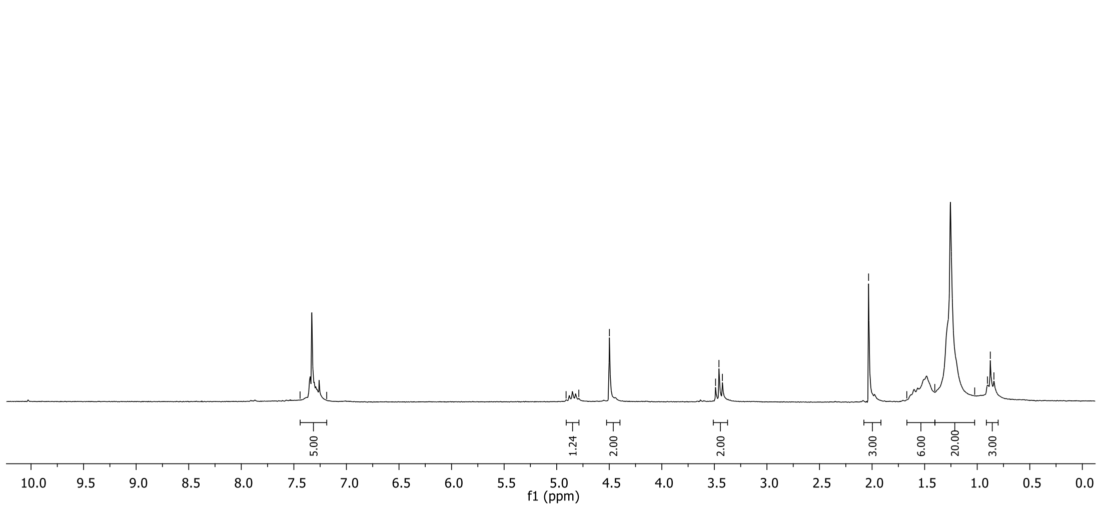
7.44
7.19
4.91
4.79
4.50
3.49
3.46
3.42
2.03
1.67
1.40
1.02
0.90
0.88
0.84
5.00
1.24
2.00
2.00
3.00
6.00
20.00
3.00
10.0 9.5 9.0 8.5 8.0 7.5 7.0 6.5 6.0 5.5 5.0 4.5 4.0 3.5 3.0 2.5 2.0 1.5 1.0 0.5 0.0
f1 (ppm)

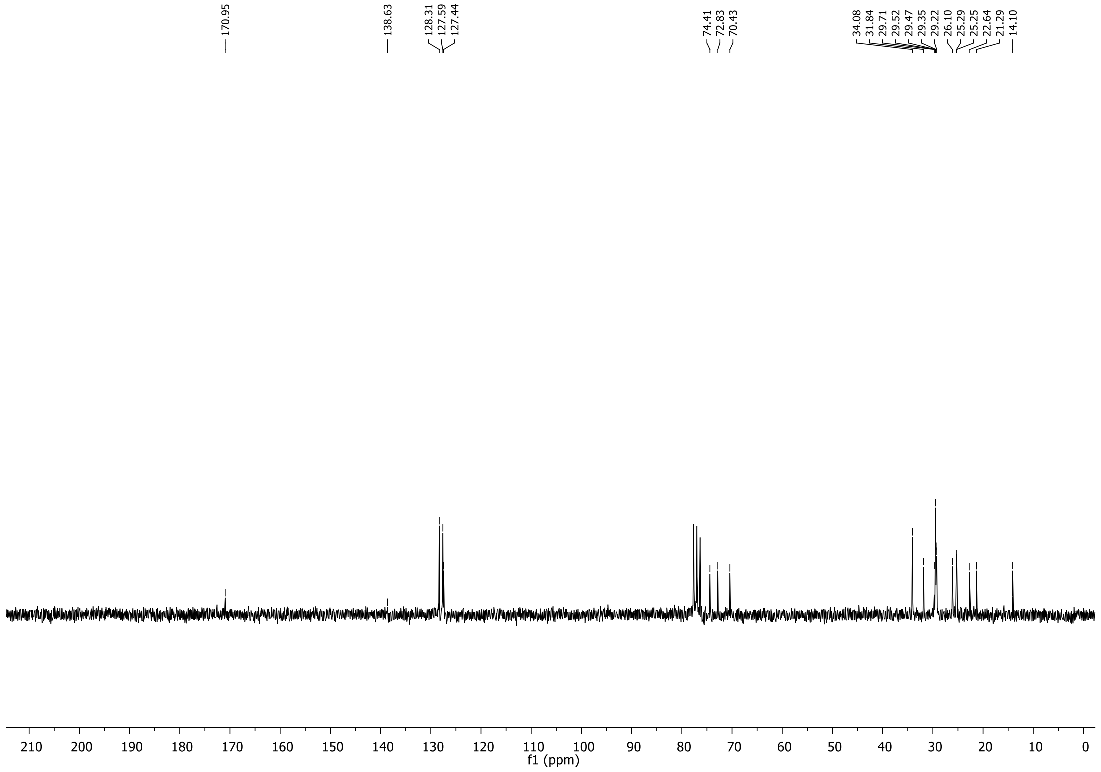
170.95
138.63
128.31
127.59
127.44
74.41
72.83
70.43
34.08
31.84
29.71
29.52
29.47
29.35
29.22
26.10
25.29
25.25
22.64
21.29
14.10
210 200 190 180 170 160 150 140 130 120 110 100 90 80 70 60 50 40 30 20 10 0
f1 (ppm)

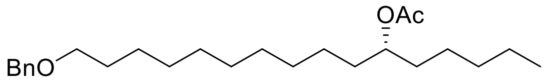
OAc
BnO

13c

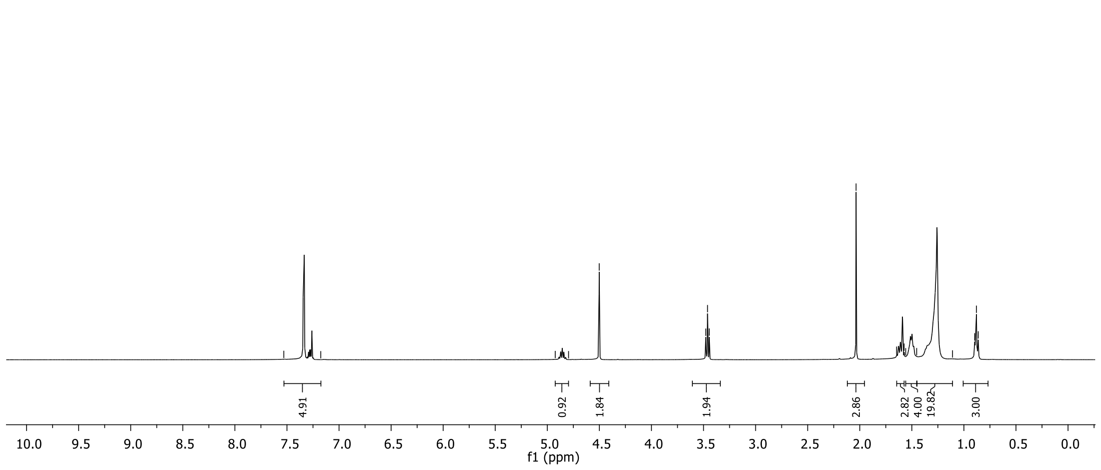
7.53
7.18
4.92
4.80
4.50
3.48
3.46
3.44
2.04
1.65
1.57
1.56
1.45
1.11
0.90
0.88
0.86
4.91
0.92
1.84
1.94
2.86
2.82
4.00
19.82
3.00
f1 (ppm)

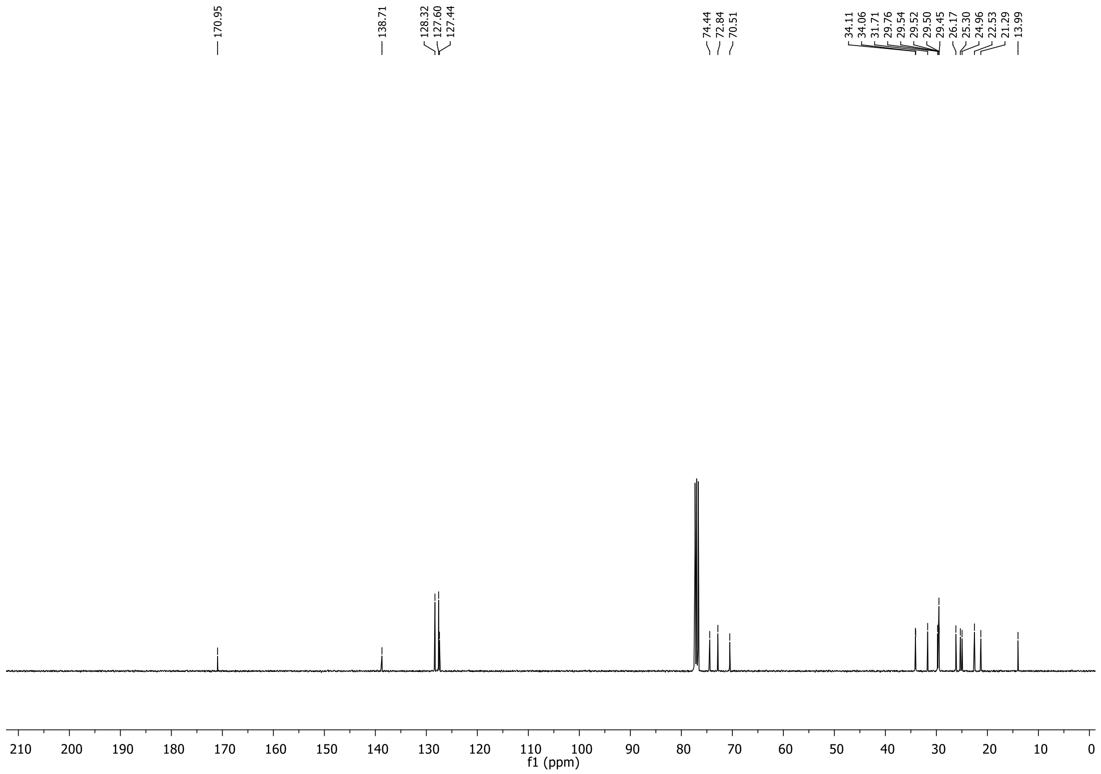
170.95
138.71
128.32
127.60
127.44
74.44
72.84
70.51
34.11
34.06
31.71
29.76
29.54
29.52
29.50
29.45
26.17
25.30
24.96
22.53
21.29
13.99
f1 (ppm)

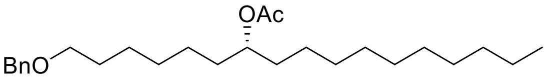
OAc
BnO

13d

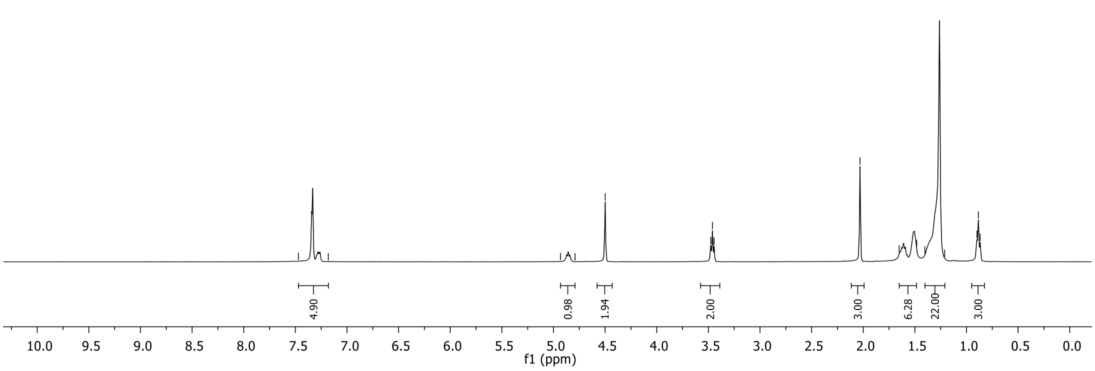
7.47
7.18
4.93
4.79
4.50
3.48
3.46
3.44
2.03
1.65
1.48
1.40
1.21
0.90
0.88
0.87
4.90
0.98
1.94
2.00
3.00
6.28
22.00
3.00
10.0 9.5 9.0 8.5 8.0 7.5 7.0 6.5 6.0 5.5 5.0 4.5 4.0 3.5 3.0 2.5 2.0 1.5 1.0 0.5 0.0
f1 (ppm)

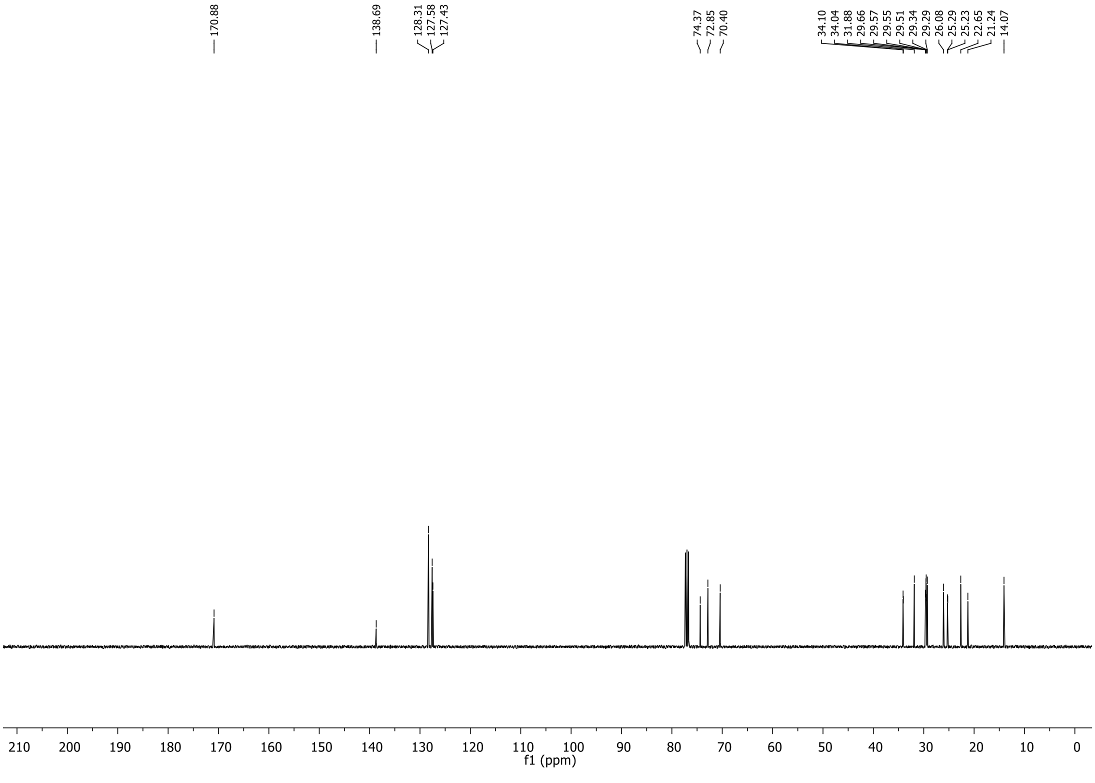
170.88
138.69
128.31
127.58
127.43
74.37
72.85
70.40
34.10
34.04
31.88
29.66
29.57
29.55
29.51
29.34
29.29
26.08
25.29
25.23
22.65
21.24
14.07
210 200 190 180 170 160 150 140 130 120 110 100 90 80 70 60 50 40 30 20 10 0
f1 (ppm)

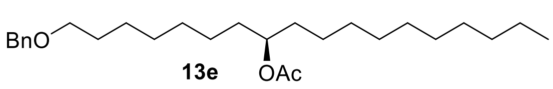
BnO
OAc

13e

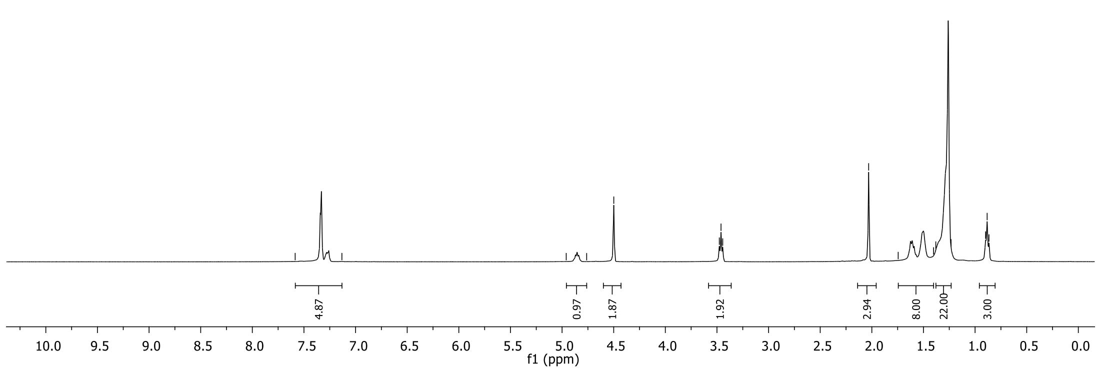
7.58
7.13
4.96
4.76
4.50
3.48
3.46
3.44
2.03
1.74
1.40
1.38
1.23
0.90
0.88
0.87
4.87
0.97
1.87
1.92
2.94
8.00
22.00
3.00
10.0 9.5 9.0 8.5 8.0 7.5 7.0 6.5 6.0 5.5 5.0 4.5 4.0 3.5 3.0 2.5 2.0 1.5 1.0 0.5 0.0
f1 (ppm)

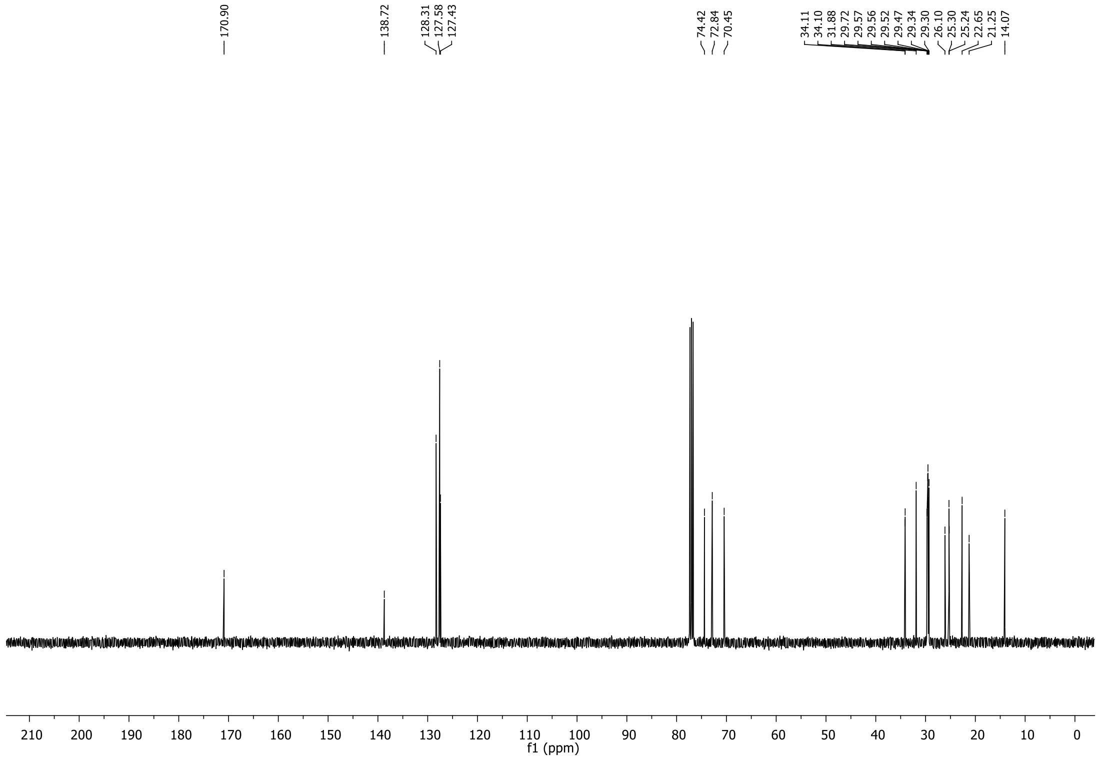
170.90
138.72
128.31
127.58
127.43
74.42
72.84
70.45
34.11
34.10
31.88
29.72
29.57
29.56
29.52
29.47
29.34
29.30
26.10
25.30
25.24
22.65
21.25
14.07
210 200 190 180 170 160 150 140 130 120 110 100 90 80 70 60 50 40 30 20 10 0
f1 (ppm)

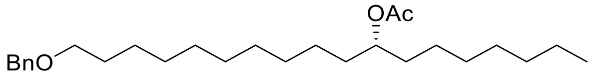
OAc
BnO

13f

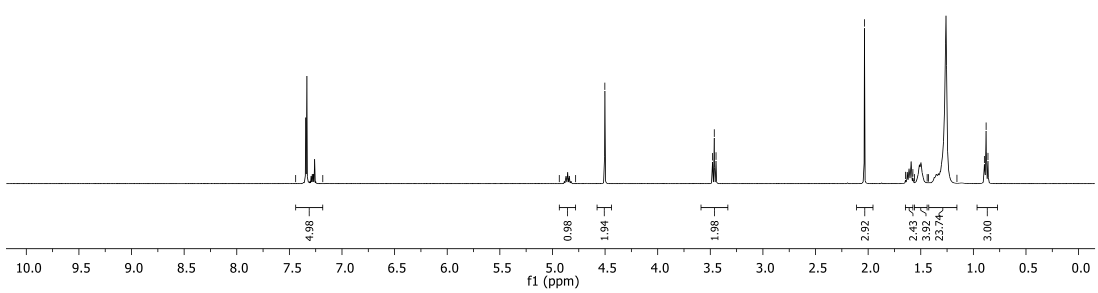
7.44
7.18
4.94
4.78
4.50
3.48
3.46
3.45
2.04
1.65
1.57
1.56
1.44
1.43
1.16
0.90
0.88
0.86
4.98
0.98
1.94
1.98
2.92
2.43
3.92
23.74
3.00
10.0 9.5 9.0 8.5 8.0 7.5 7.0 6.5 6.0 5.5 5.0 4.5 4.0 3.5 3.0 2.5 2.0 1.5 1.0 0.5 0.0
f1 (ppm)

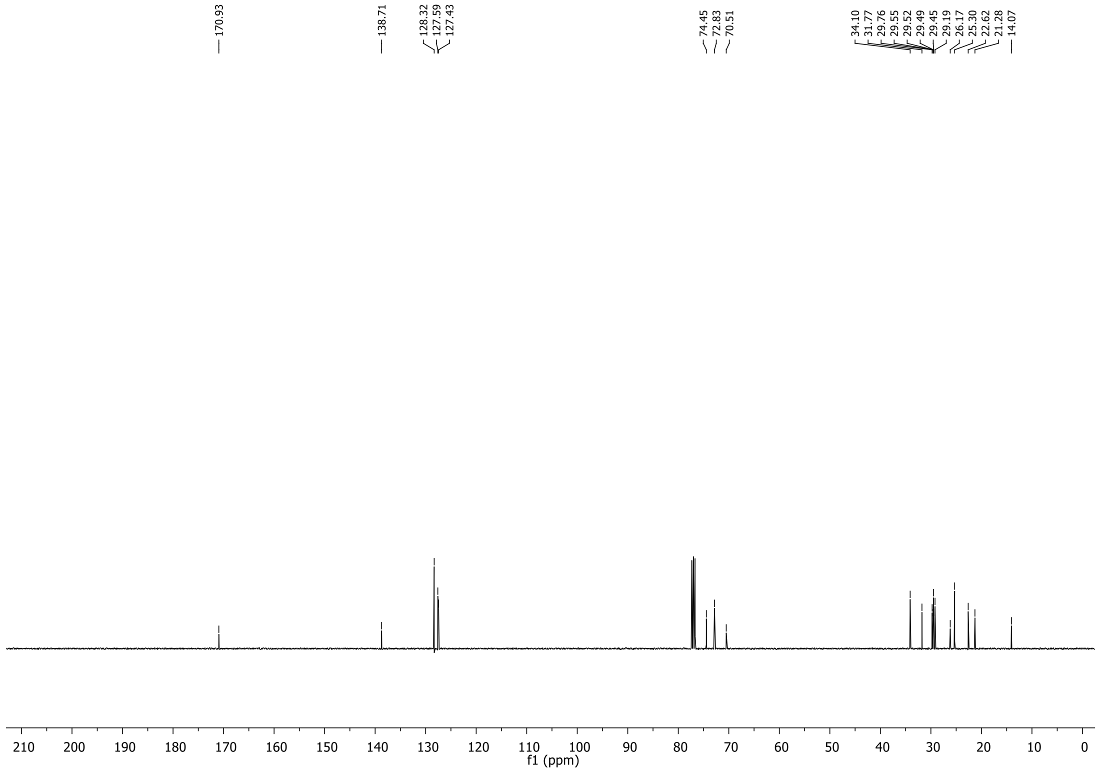
170.93
138.71
128.32
127.59
127.43
74.45
72.83
70.51
34.10
31.77
29.76
29.55
29.52
29.49
29.45
29.19
26.17
25.30
22.62
21.28
14.07
210 200 190 180 170 160 150 140 130 120 110 100 90 80 70 60 50 40 30 20 10 0
f1 (ppm)

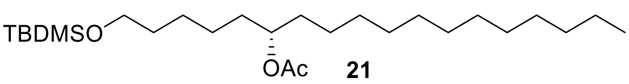
TBDMSO
OAc

21

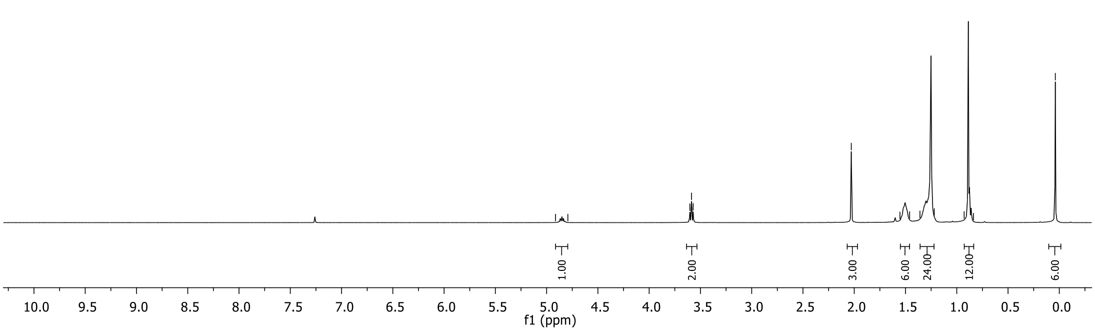
4.91
4.79
3.60
3.59
3.57
2.03
1.55
1.46
1.36
1.22
0.93
0.84
0.04
1.00
2.00
3.00
6.00
24.00
12.00
6.00
10.0 9.5 9.0 8.5 8.0 7.5 7.0 6.5 6.0 5.5 5.0 4.5 4.0 3.5 3.0 2.5 2.0 1.5 1.0 0.5 0.0
f1 (ppm)

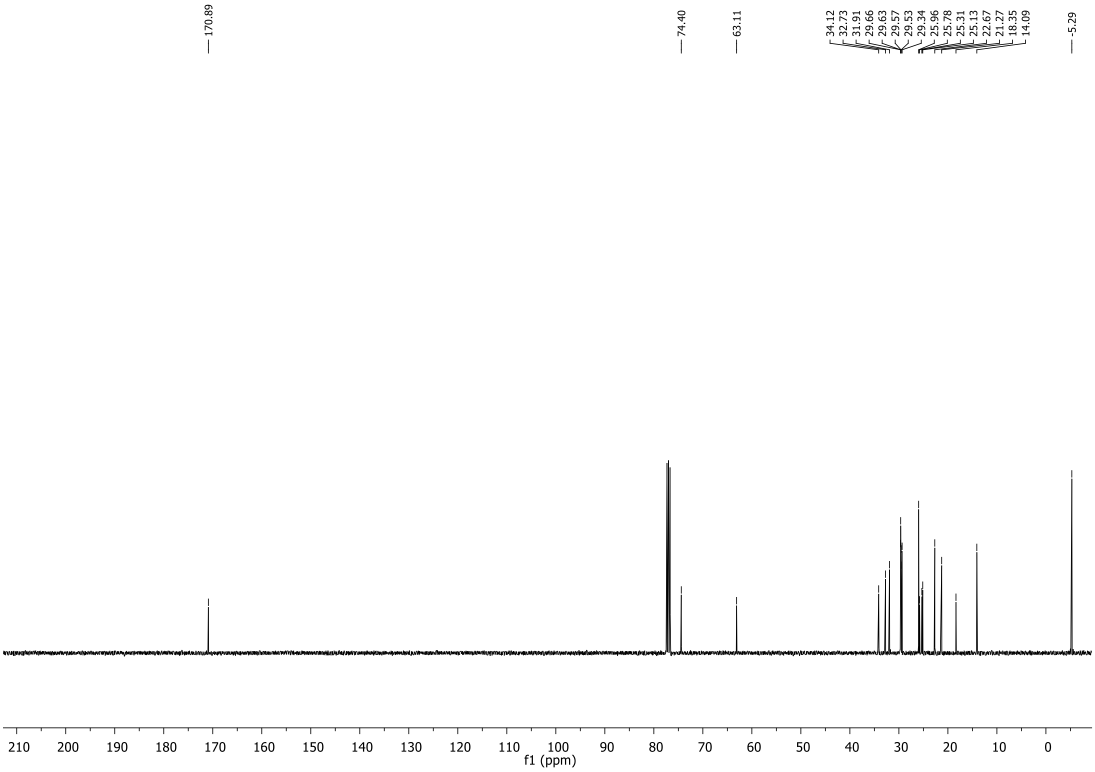
170.89
74.40
63.11
34.12
32.73
31.91
29.66
29.63
29.57
29.53
29.34
25.96
25.78
25.31
25.13
22.67
21.27
18.35
14.09
-5.29
210 200 190 180 170 160 150 140 130 120 110 100 90 80 70 60 50 40 30 20 10 0
f1 (ppm)

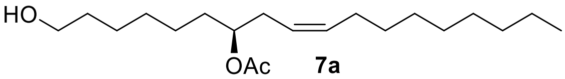
HO
OAc

7a

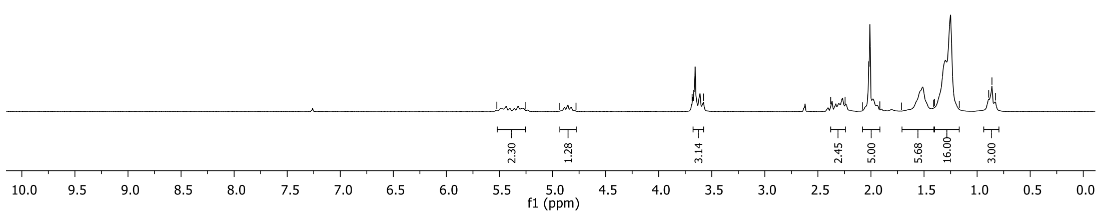
5.52
5.25
4.94
4.78
3.68
3.58
2.38
2.24
2.08
1.92
1.71
1.41
1.40
1.17
0.89
0.86
0.83
2.30
1.28
3.14
2.45
5.00
5.68
16.00
3.00
10.0 9.5 9.0 8.5 8.0 7.5 7.0 6.5 6.0 5.5 5.0 4.5 4.0 3.5 3.0 2.5 2.0 1.5 1.0 0.5 0.0
f1 (ppm)

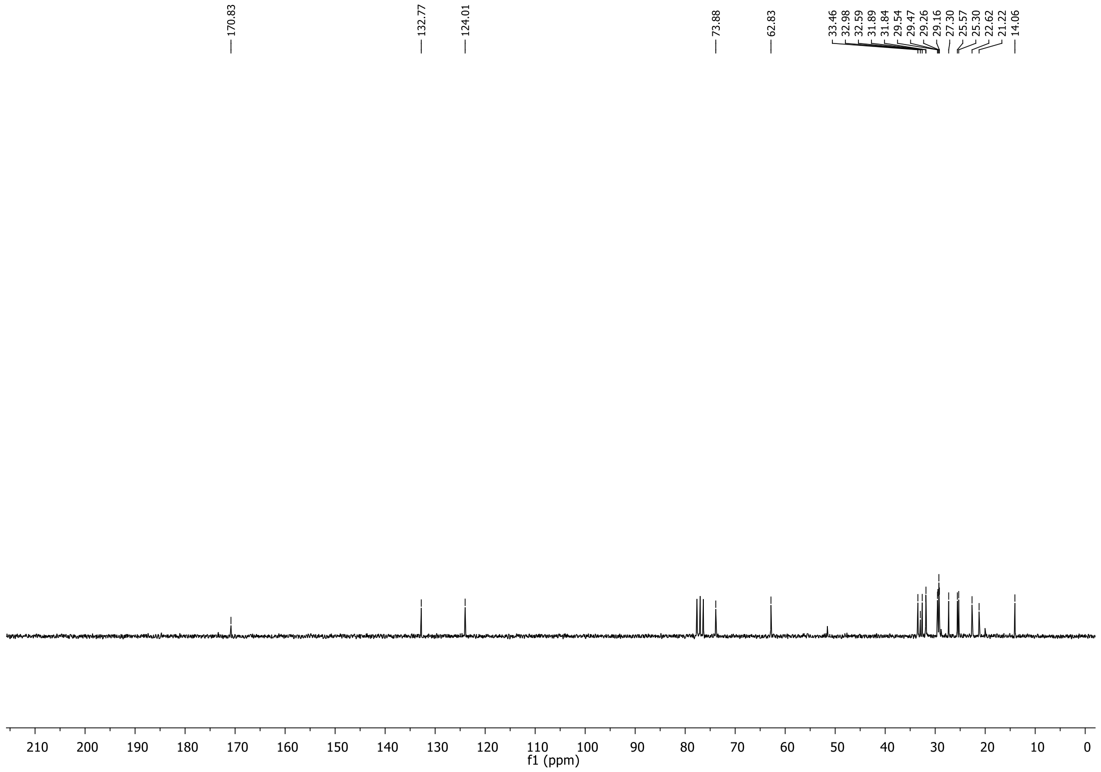
170.83
132.77
124.01
73.88
62.83
33.46
32.98
32.59
31.89
31.84
29.54
29.47
29.26
29.16
27.30
25.57
25.30
22.62
21.22
14.06
210 200 190 180 170 160 150 140 130 120 110 100 90 80 70 60 50 40 30 20 10 0
f1 (ppm)

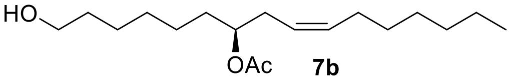
HO
OAc

7b

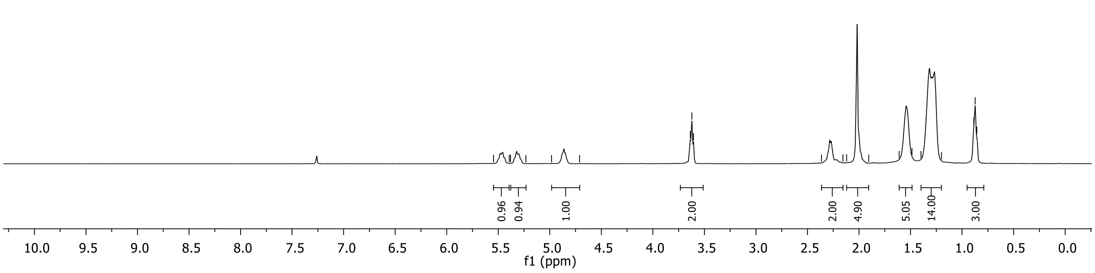
5.55
5.39
5.38
5.23
4.98
4.71
3.64
3.62
3.61
2.36
2.16
2.12
1.91
1.61
1.49
1.40
1.20
0.89
0.87
0.86
0.96
0.94
1.00
2.00
2.00
4.90
5.05
14.00
3.00
10.0 9.5 9.0 8.5 8.0 7.5 7.0 6.5 6.0 5.5 5.0 4.5 4.0 3.5 3.0 2.5 2.0 1.5 1.0 0.5 0.0
f1 (ppm)

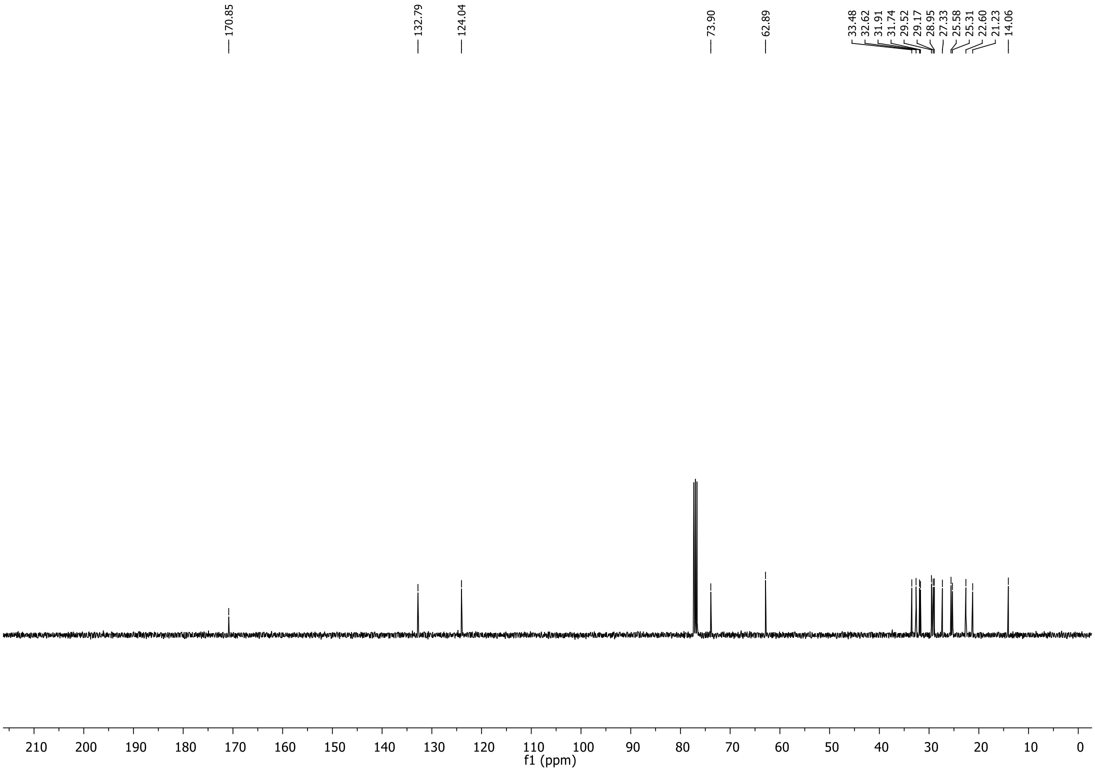
170.85
132.79
124.04
73.90
62.89
33.48
32.62
31.91
31.74
29.52
29.17
28.95
27.33
25.58
25.31
22.60
21.23
14.06
210 200 190 180 170 160 150 140 130 120 110 100 90 80 70 60 50 40 30 20 10 0
f1 (ppm)

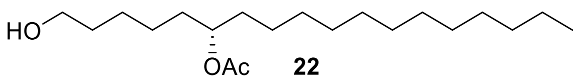
HO
OAc

22

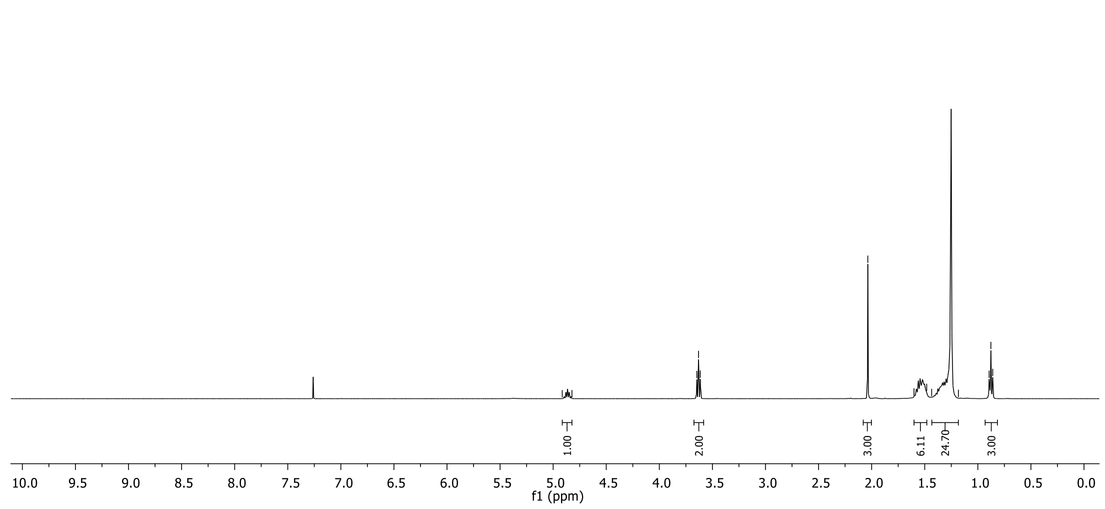
4.91
4.82
3.65
3.63
3.61
2.04
1.60
1.48
1.43
1.18
0.89
0.88
0.86
1.00
2.00
3.00
6.11
24.70
3.00
10.0 9.5 9.0 8.5 8.0 7.5 7.0 6.5 6.0 5.5 5.0 4.5 4.0 3.5 3.0 2.5 2.0 1.5 1.0 0.5 0.0
f1 (ppm)

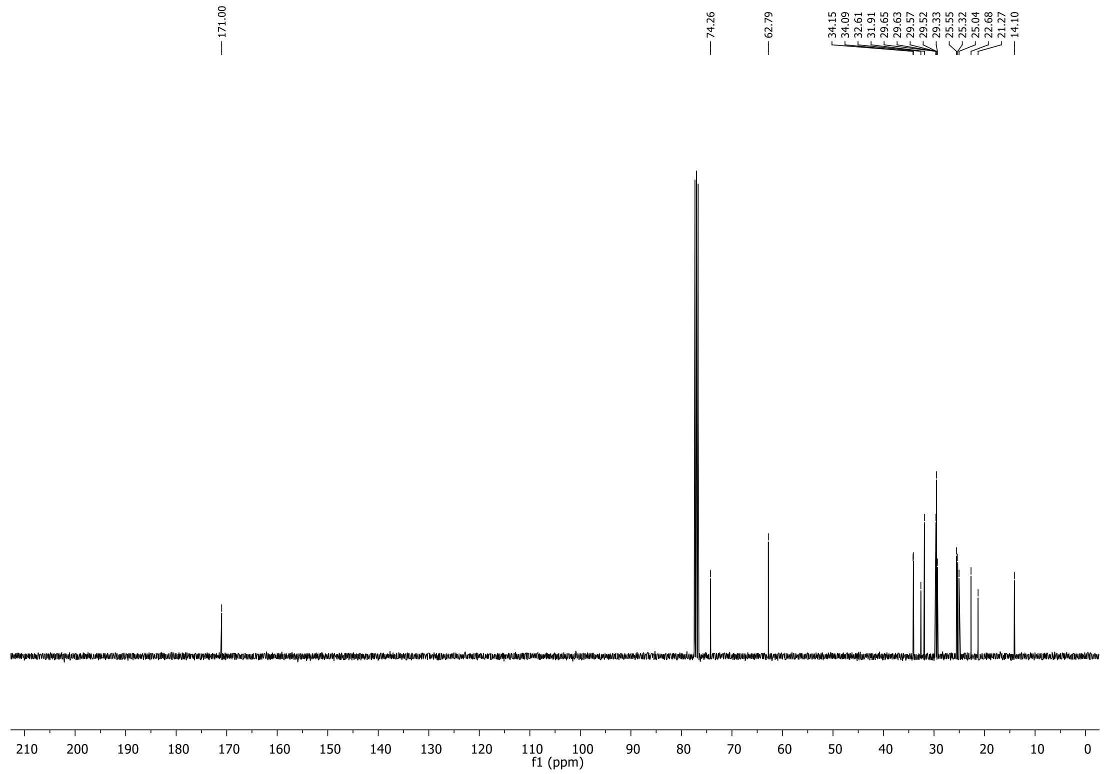
171.00
74.26
62.79
34.15
34.09
32.61
31.91
29.65
29.63
29.57
29.52
29.33
25.55
25.32
25.04
22.68
21.27
14.10
210 200 190 180 170 160 150 140 130 120 110 100 90 80 70 60 50 40 30 20 10 0
f1 (ppm)

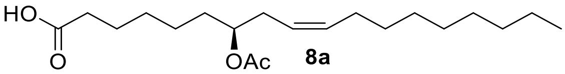
HO
O
OAc

8a

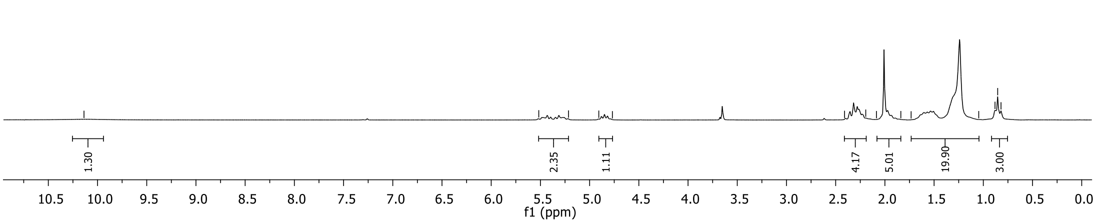
10.14
5.52
5.21
4.91
4.77
2.41
2.19
2.09
1.84
1.73
1.05
0.88
0.85
0.82
1.30
2.35
1.11
4.17
5.01
19.90
3.00
f1 (ppm)

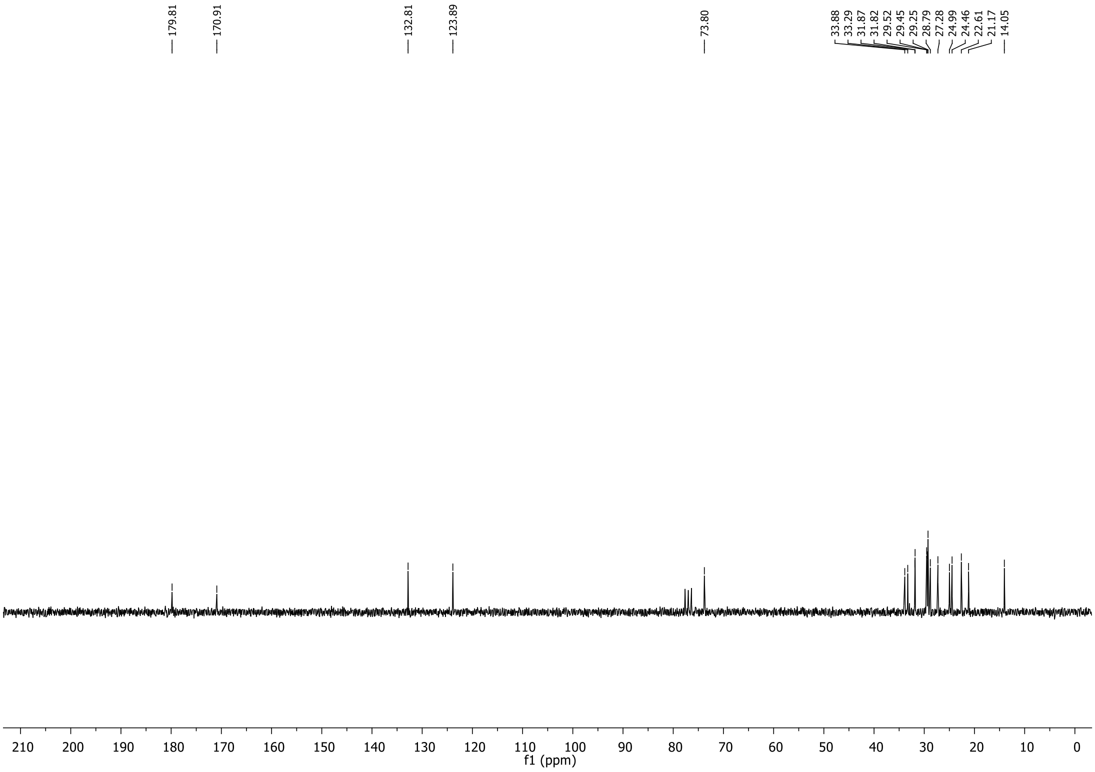
179.81
170.91
132.81
123.89
73.80
33.88
33.29
31.87
31.82
29.52
29.45
29.25
28.79
27.28
24.99
24.46
22.61
21.17
14.05
f1 (ppm)

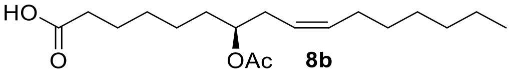
HO
O
OAc

8b

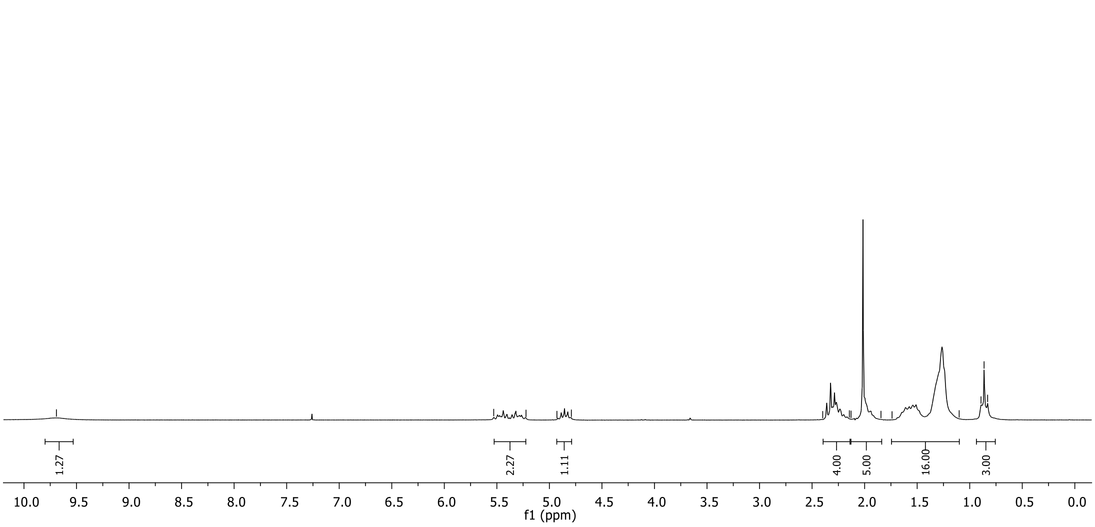
9.69
5.53
5.22
4.93
4.79
2.40
2.15
2.13
1.84
1.74
1.10
0.89
0.86
0.83
1.27
2.27
1.11
4.00
5.00
16.00
3.00
10.0 9.5 9.0 8.5 8.0 7.5 7.0 6.5 6.0 5.5 5.0 4.5 4.0 3.5 3.0 2.5 2.0 1.5 1.0 0.5 0.0
f1 (ppm)

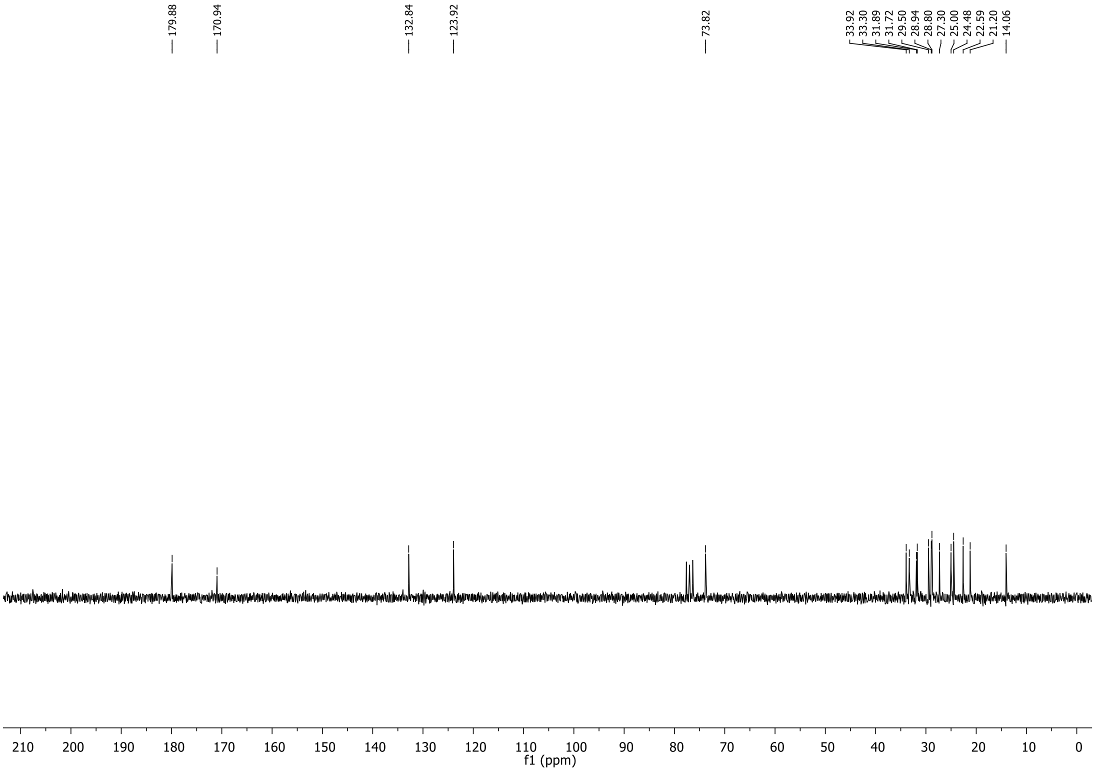
179.88
170.94
132.84
123.92
73.82
33.92
33.30
31.89
31.72
29.50
28.94
28.80
27.30
25.00
24.48
22.59
21.20
14.06
210 200 190 180 170 160 150 140 130 120 110 100 90 80 70 60 50 40 30 20 10 0
f1 (ppm)

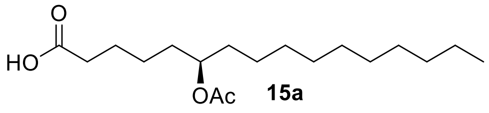

15a

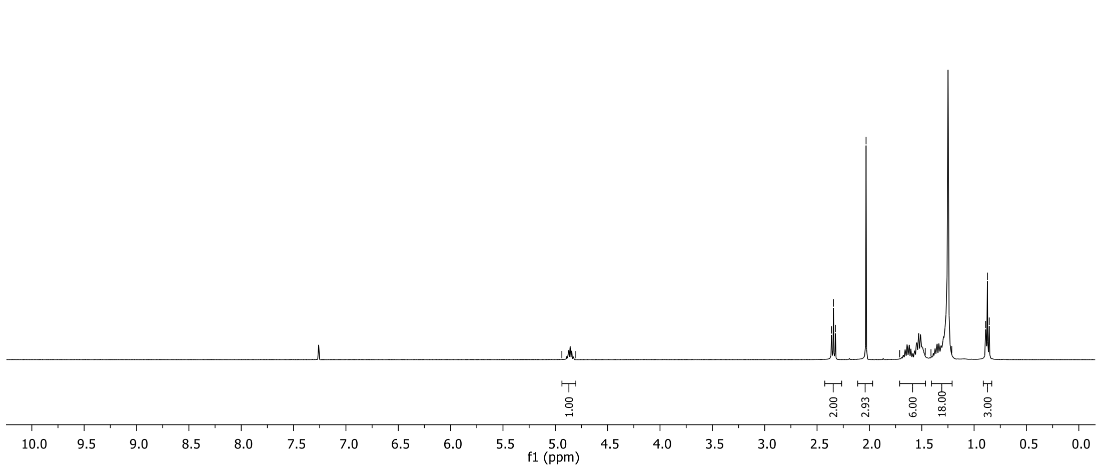

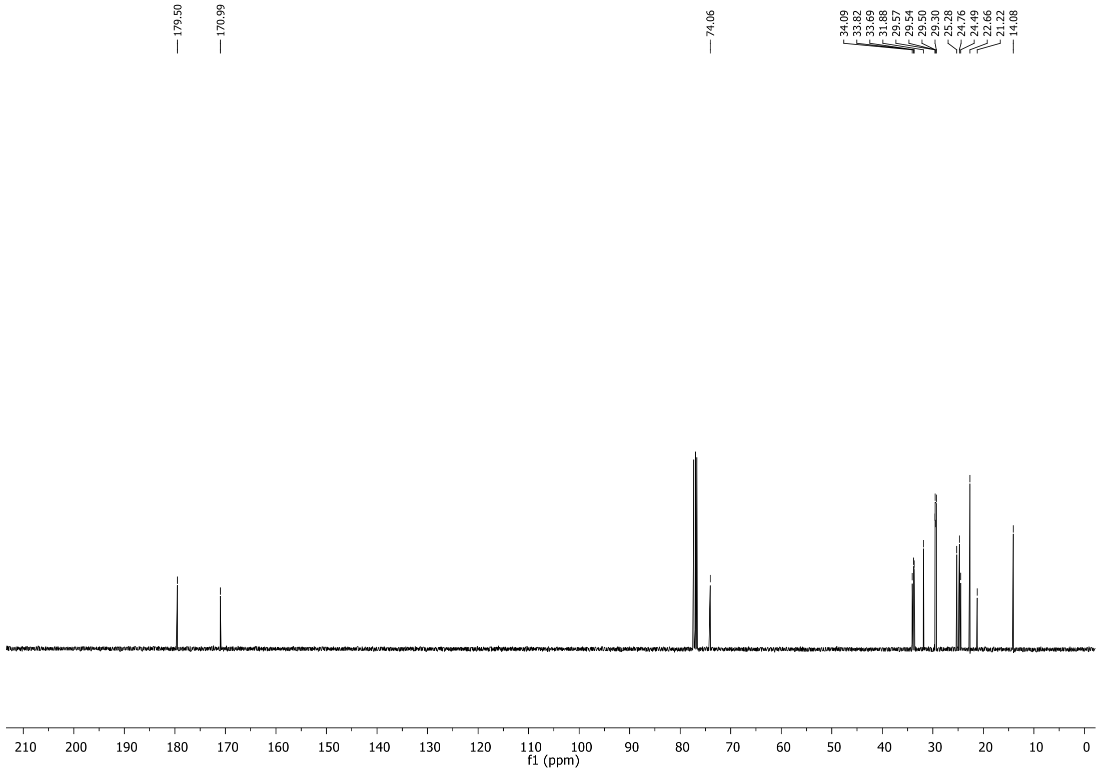

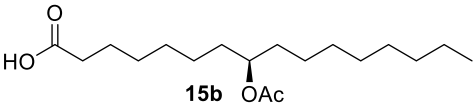
HO
O
OAc

15b

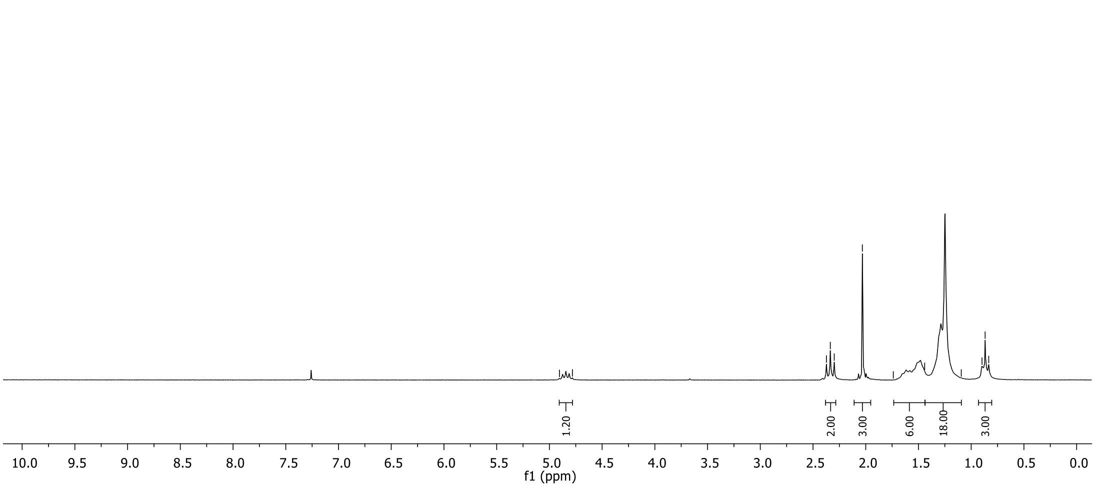
4.90
4.78
2.37
2.34
2.30
2.03
1.74
1.44
1.09
0.90
0.87
0.83
1.20
2.00
3.00
6.00
18.00
3.00
f1 (ppm)

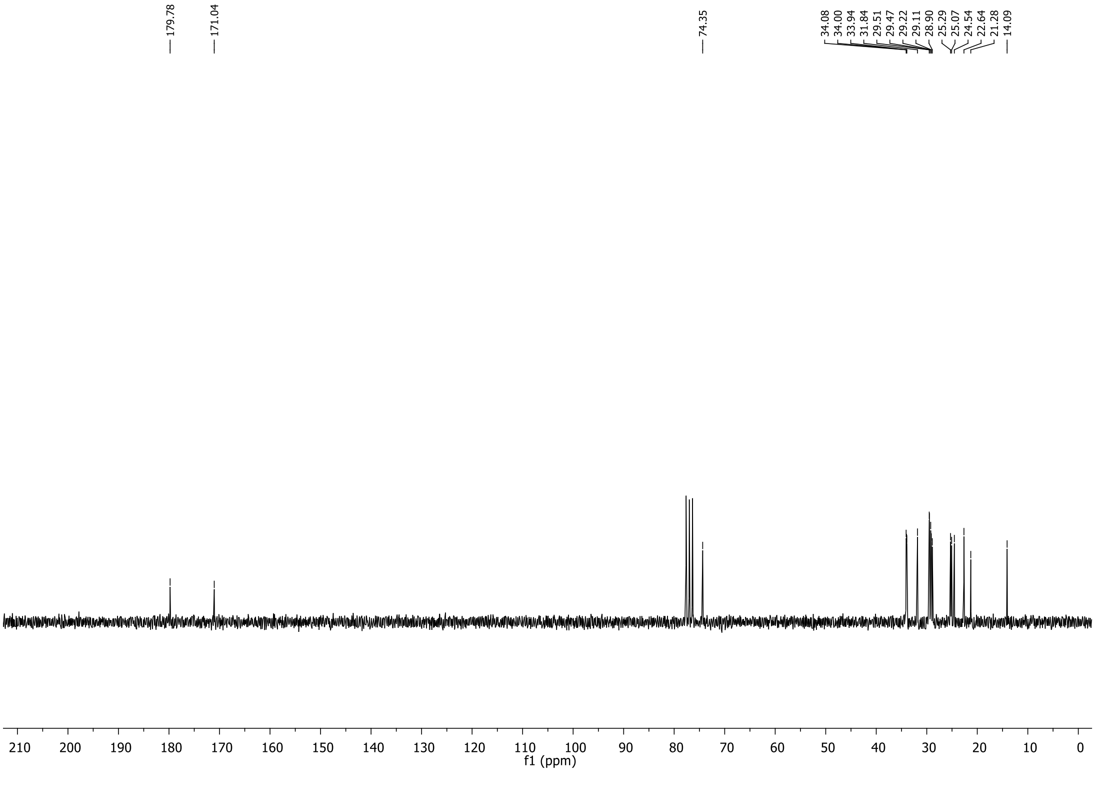
179.78
171.04
74.35
34.08
34.00
33.94
31.84
29.51
29.47
29.22
29.11
28.90
25.29
25.07
24.54
22.64
21.28
14.09
f1 (ppm)

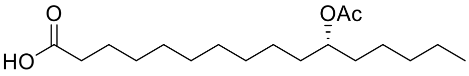
O
OAc
HO

15c

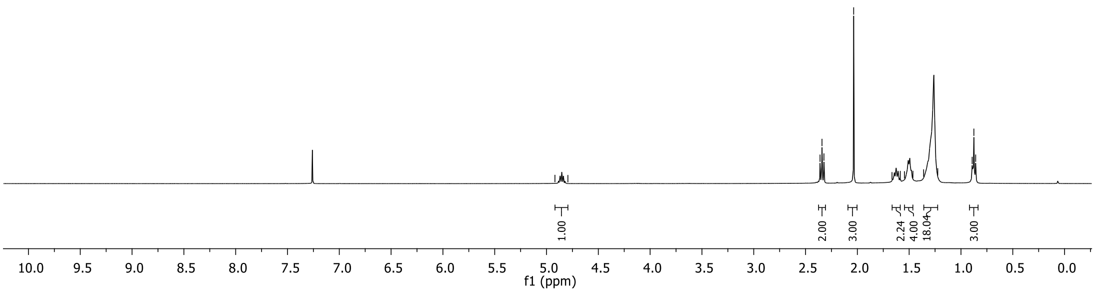
4.92
4.79
2.36
2.34
2.32
2.04
1.67
1.59
1.55
1.46
1.36
1.22
0.89
0.88
0.86
1.00
2.00
3.00
2.24
4.00
18.04
3.00
10.0 9.5 9.0 8.5 8.0 7.5 7.0 6.5 6.0 5.5 5.0 4.5 4.0 3.5 3.0 2.5 2.0 1.5 1.0 0.5 0.0
f1 (ppm)

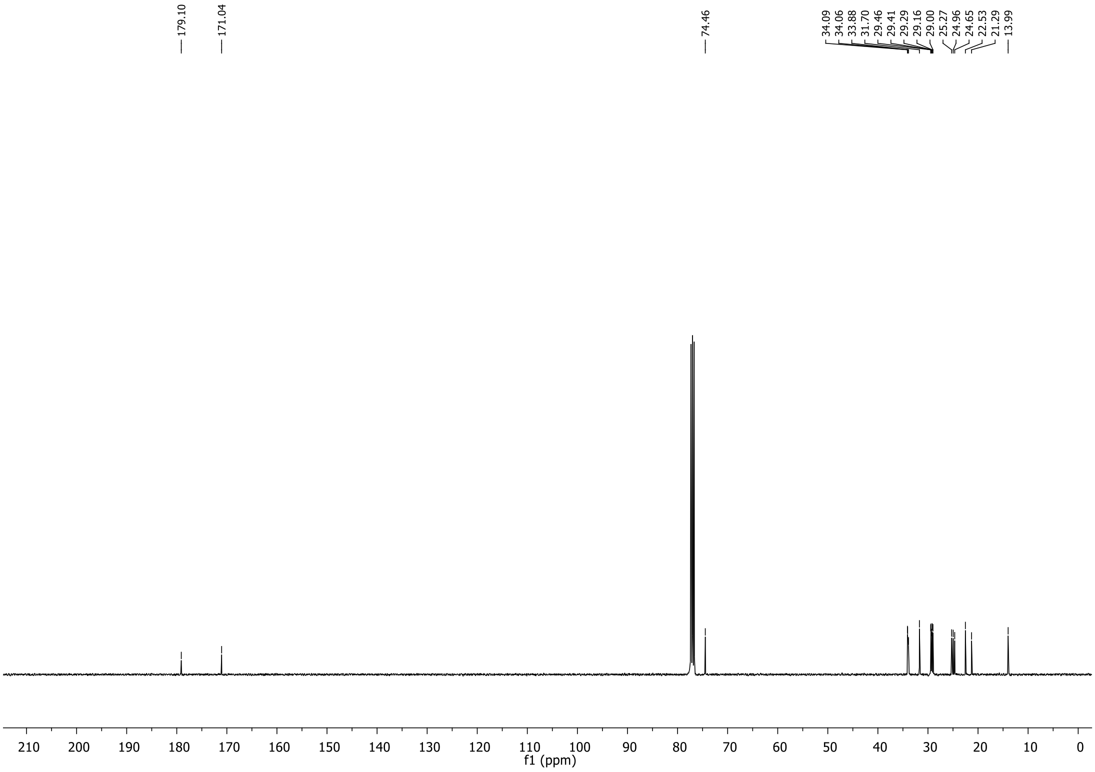
179.10
171.04
74.46
34.09
34.06
33.88
31.70
29.46
29.41
29.29
29.16
29.00
25.27
24.96
24.65
22.53
21.29
13.99
210 200 190 180 170 160 150 140 130 120 110 100 90 80 70 60 50 40 30 20 10 0
f1 (ppm)

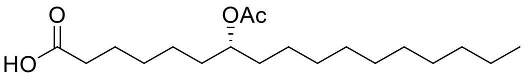
O
OAc
HO

15d

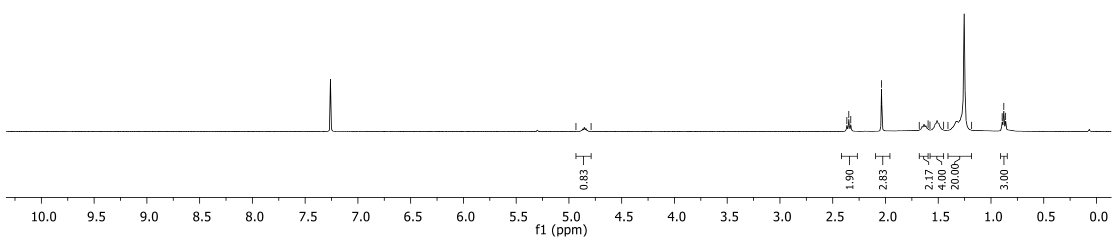
4.93
4.79
2.37
2.35
2.33
2.04
1.68
1.60
1.58
1.45
1.41
1.18
0.89
0.88
0.86
0.83
1.90
2.83
2.17
4.00
20.00
3.00
f1 (ppm)

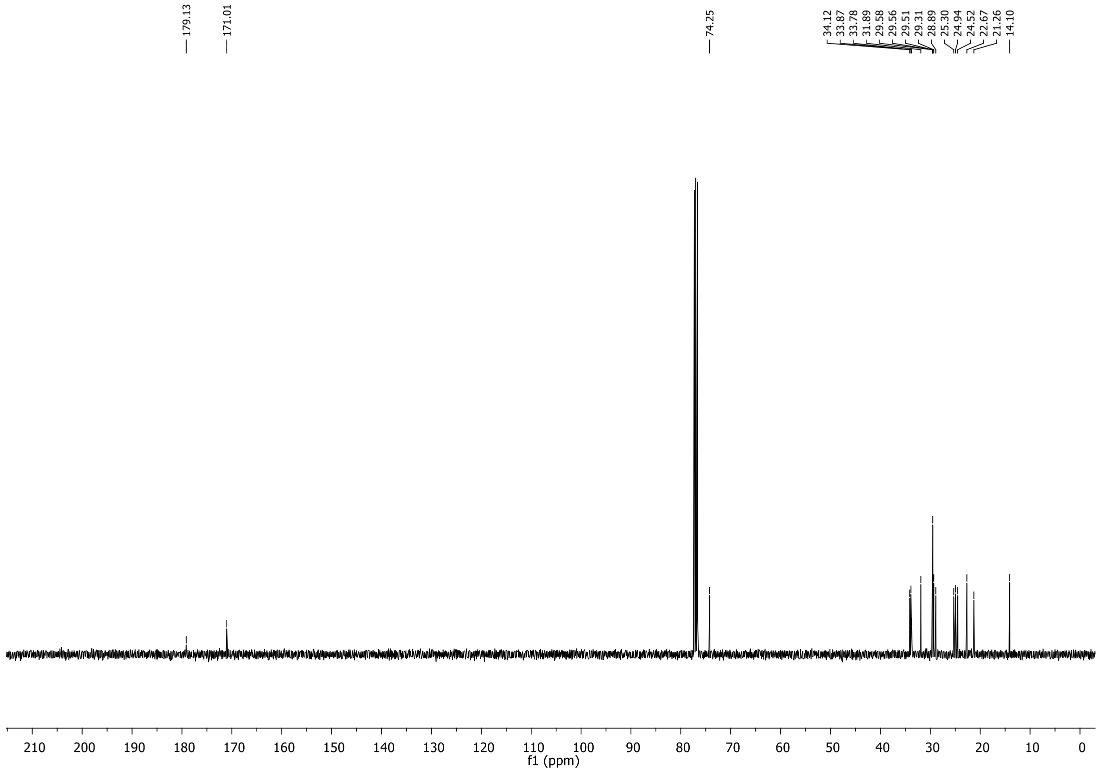
179.13
171.01
74.25
34.12
33.87
33.78
31.89
29.58
29.56
29.51
29.31
28.89
25.30
24.94
24.52
22.67
21.26
14.10
f1 (ppm)

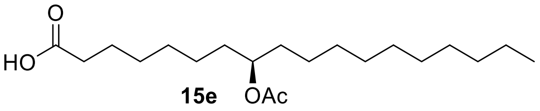
HO
O
OAc

15e

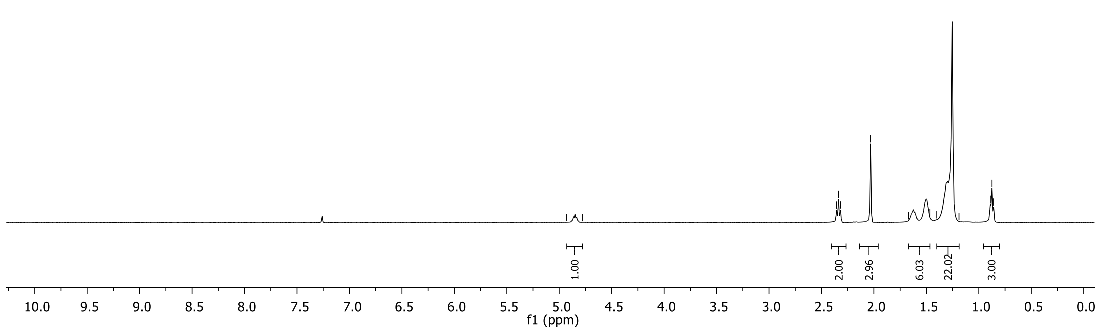
4.93
4.78
2.36
2.34
2.32
2.03
1.67
1.47
1.40
1.19
0.89
0.88
0.86
1.00
2.00
2.96
6.03
22.02
3.00
10.0 9.5 9.0 8.5 8.0 7.5 7.0 6.5 6.0 5.5 5.0 4.5 4.0 3.5 3.0 2.5 2.0 1.5 1.0 0.5 0.0
f1 (ppm)

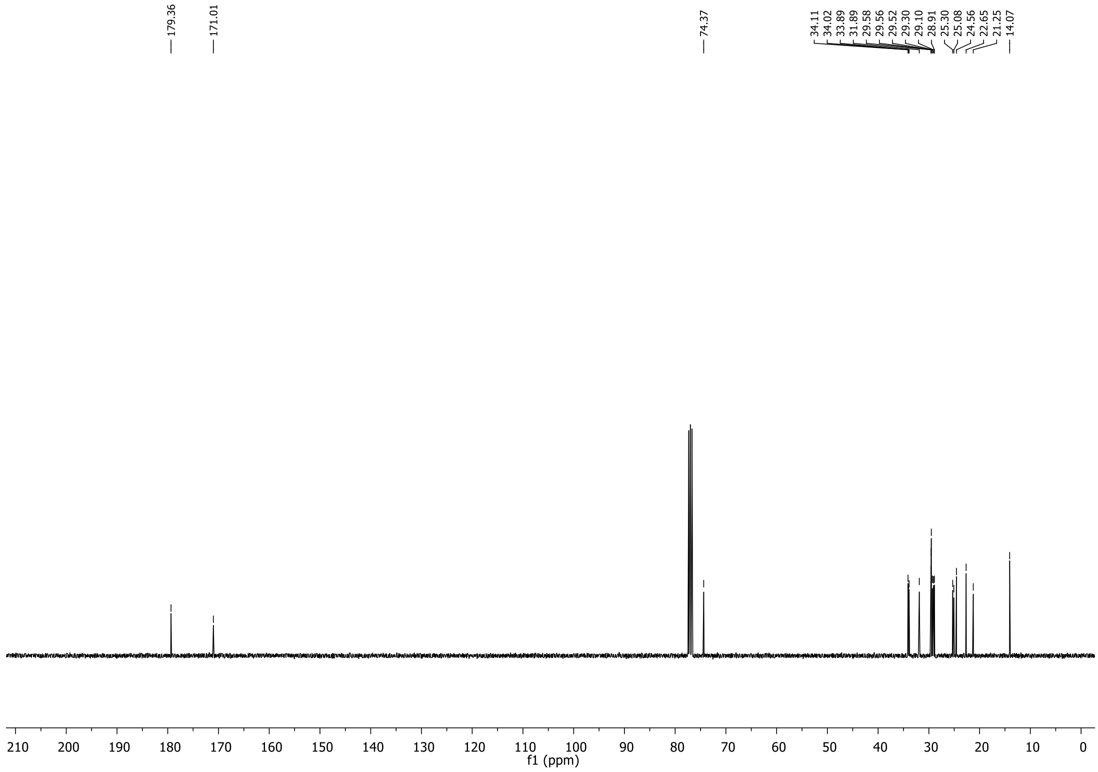
179.36
171.01
74.37
34.11
34.02
33.89
31.89
29.58
29.56
29.52
29.30
29.10
28.91
25.30
25.08
24.56
22.65
21.25
14.07
210 200 190 180 170 160 150 140 130 120 110 100 90 80 70 60 50 40 30 20 10 0
f1 (ppm)

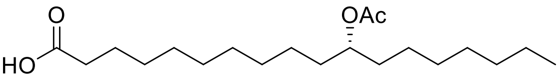
OAc

15f

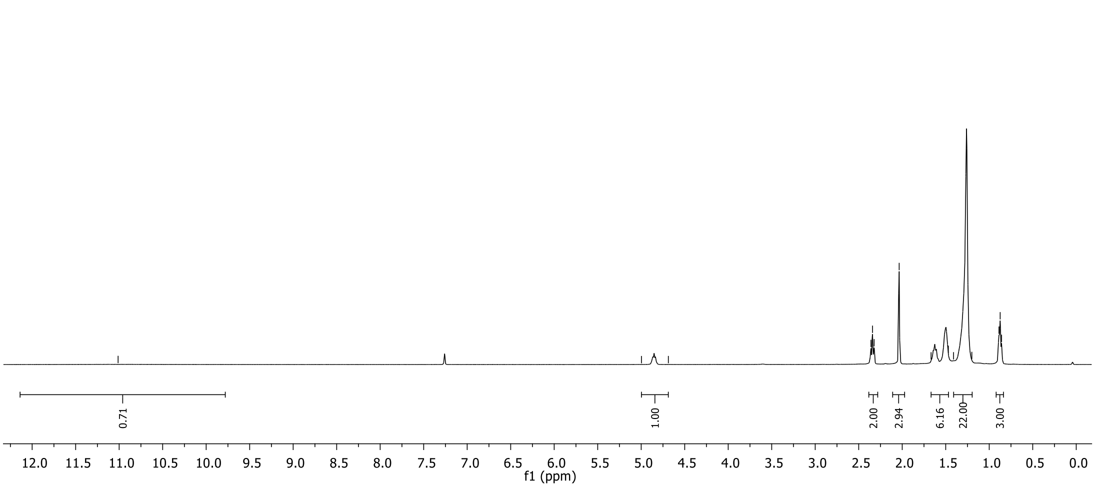
11.01
5.00
4.69
2.36
2.34
2.32
2.03
1.67
1.47
1.41
1.20
0.89
0.87
0.86
0.71
1.00
2.00
2.94
6.16
22.00
3.00
f1 (ppm)

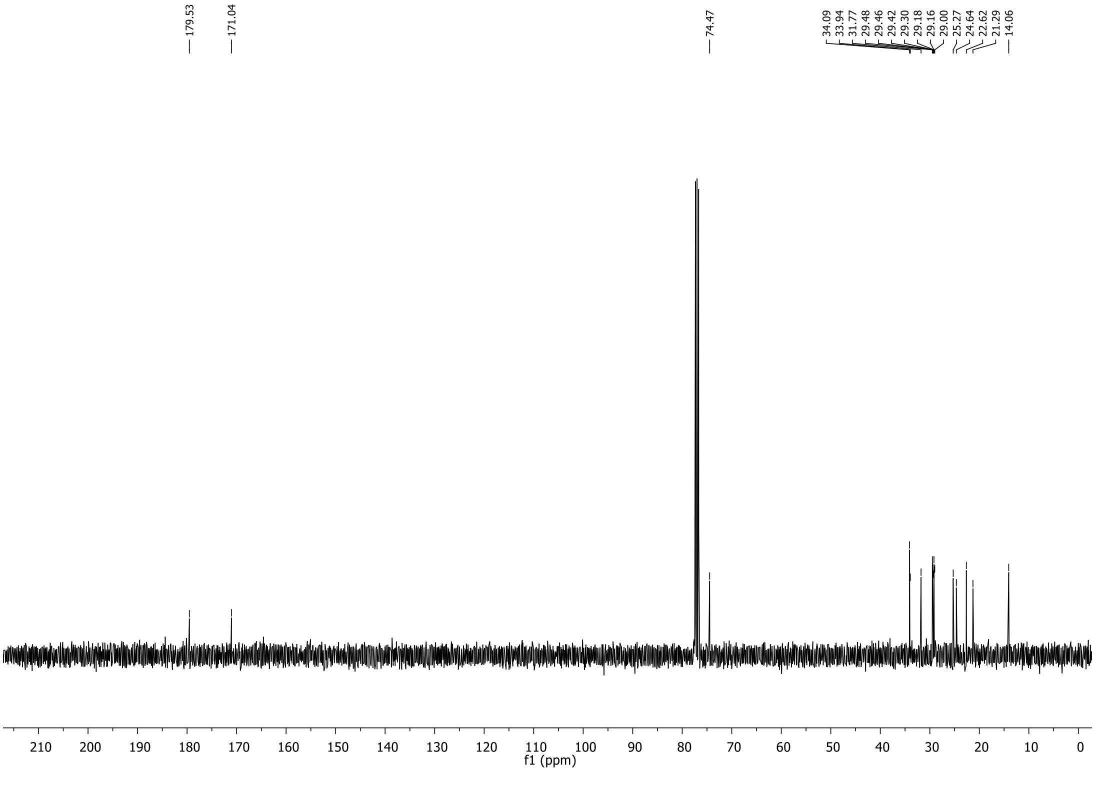
179.53
171.04
74.47
34.09
33.94
31.77
29.48
29.46
29.42
29.30
29.18
29.16
29.00
25.27
24.64
22.62
21.29
14.06
f1 (ppm)

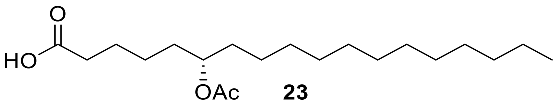

23

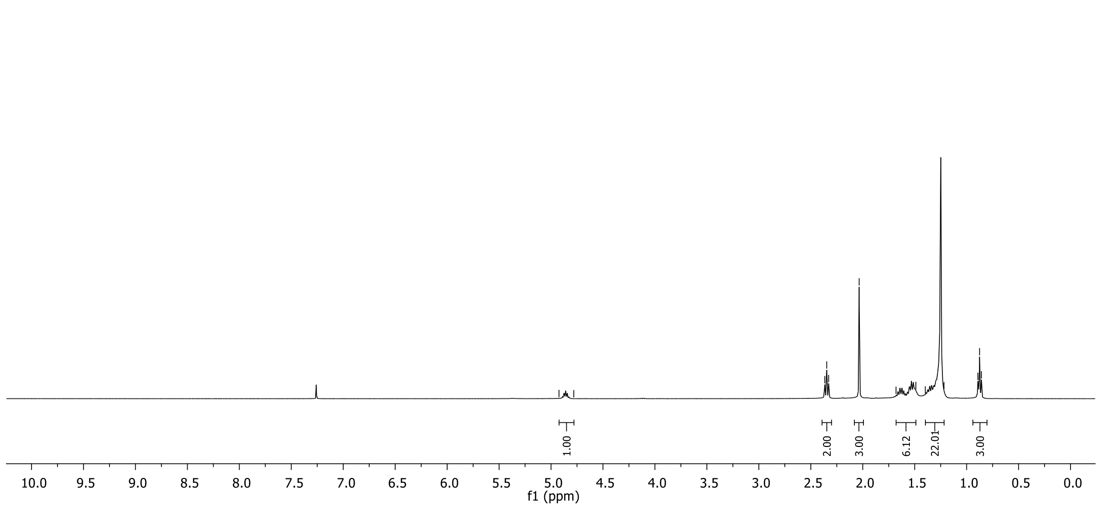
4.92
4.78
2.37
2.35
2.33
2.03
1.68
1.49
1.40
1.22
0.89
0.88
0.86
1.00
2.00
3.00
6.12
22.01
3.00
f1 (ppm)

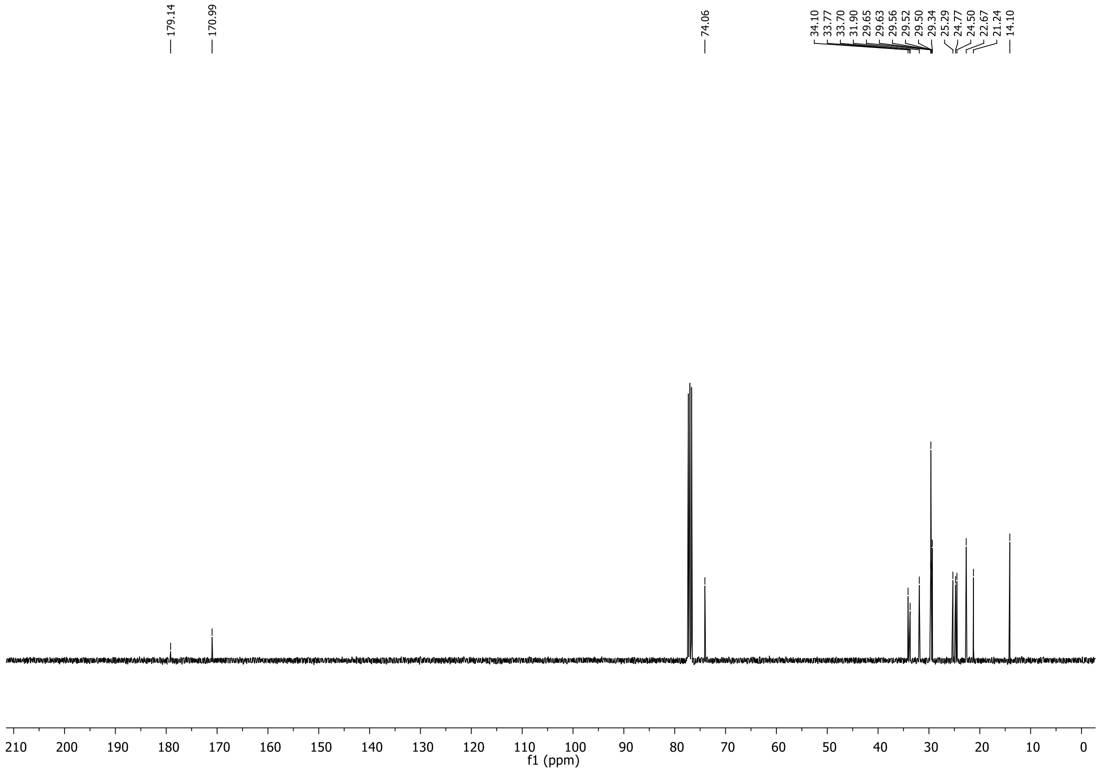
179.14
170.99
74.06
34.10
33.77
33.70
31.90
29.65
29.63
29.56
29.52
29.50
29.34
25.29
24.77
24.50
22.67
21.24
14.10
f1 (ppm)

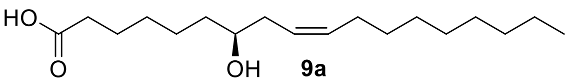
HO
O
OH

9a

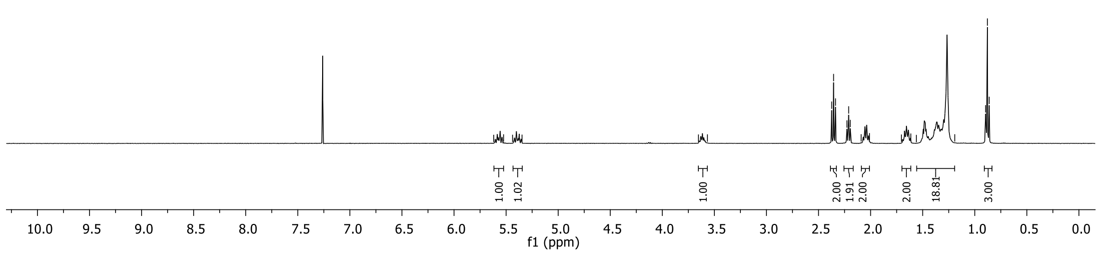
5.62
5.52
5.44
5.35
3.65
3.57
2.37
2.36
2.34
2.23
2.21
2.20
2.09
2.01
1.70
1.61
1.56
1.19
0.90
0.88
0.86
1.00
1.02
1.00
2.00
1.91
2.00
2.00
18.81
3.00
10.0 9.5 9.0 8.5 8.0 7.5 7.0 6.5 6.0 5.5 5.0 4.5 4.0 3.5 3.0 2.5 2.0 1.5 1.0 0.5 0.0
f1 (ppm)

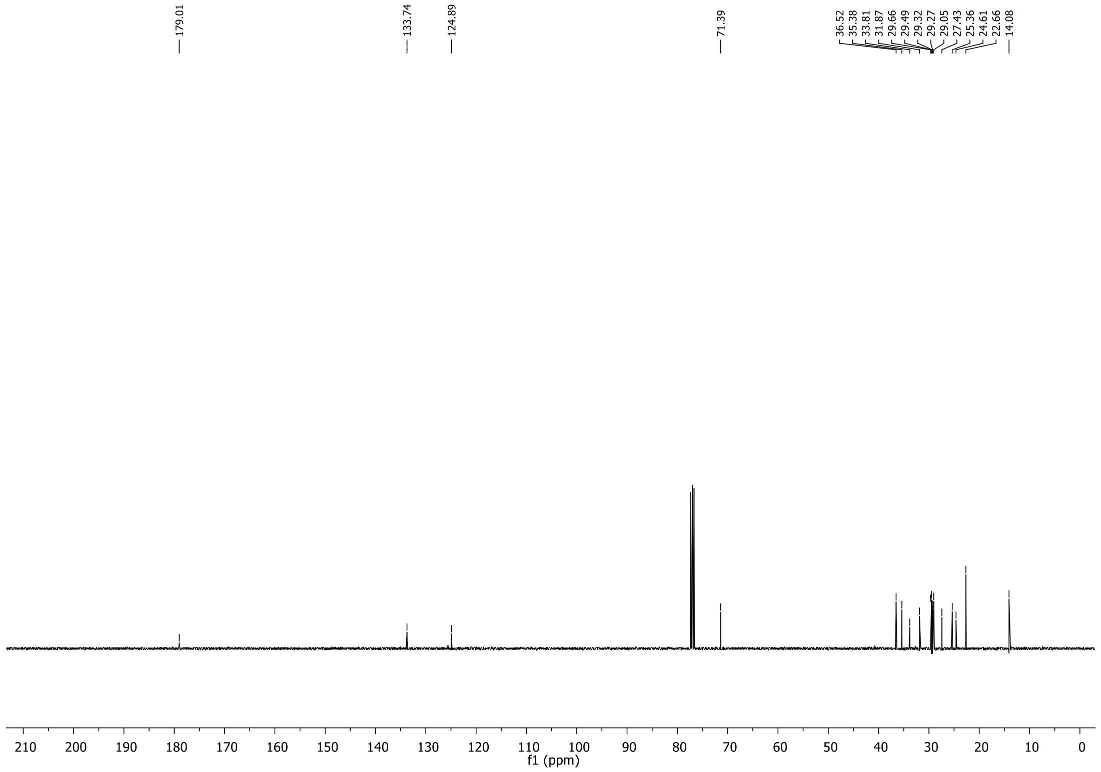
179.01
133.74
124.89
71.39
36.52
35.38
33.81
31.87
29.66
29.49
29.32
29.27
29.05
27.43
25.36
24.61
22.66
14.08
210 200 190 180 170 160 150 140 130 120 110 100 90 80 70 60 50 40 30 20 10 0
f1 (ppm)

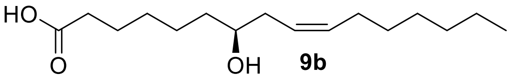
HO
O
OH

9b

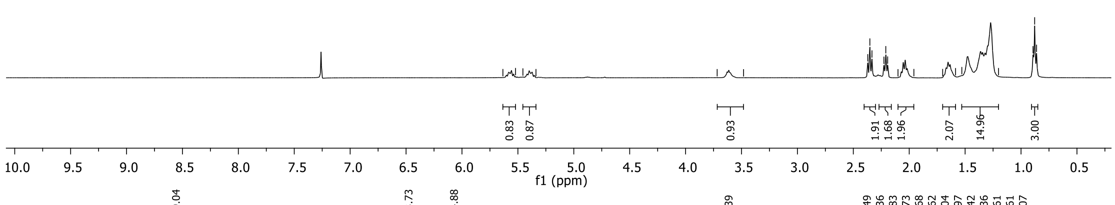
5.63
5.52
5.46
5.34
3.72
3.48
2.37
2.35
2.33
2.23
2.21
2.19
2.10
1.96
1.70
1.59
1.53
1.20
0.89
0.88
0.86
0.83
0.87
0.93
1.91
1.68
1.96
2.07
14.96
3.00
10.0 9.5 9.0 8.5 8.0 7.5 7.0 6.5 6.0 5.5 5.0 4.5 4.0 3.5 3.0 2.5 2.0 1.5 1.0 0.5
f1 (ppm)

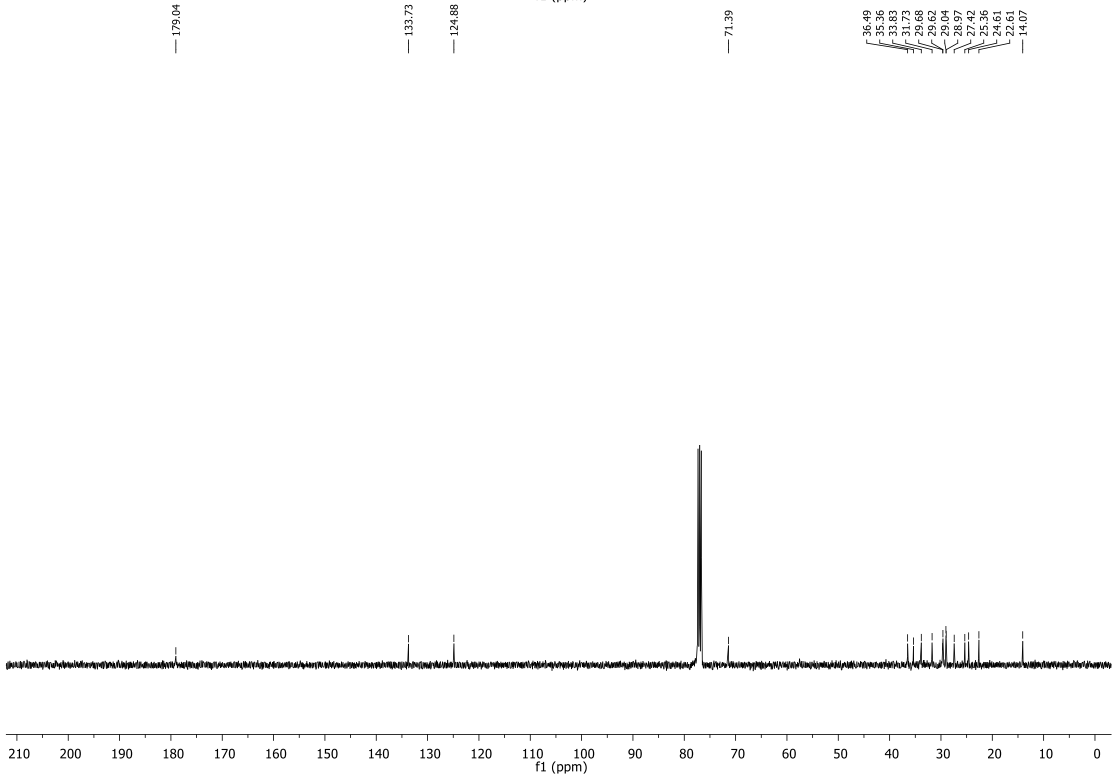
179.04
133.73
124.88
71.39
36.49
35.36
33.83
31.73
29.68
29.62
29.04
28.97
27.42
25.36
24.61
22.61
14.07
210 200 190 180 170 160 150 140 130 120 110 100 90 80 70 60 50 40 30 20 10 0
f1 (ppm)

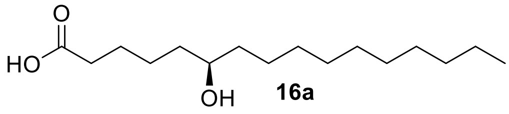
O
HO
OH

16a

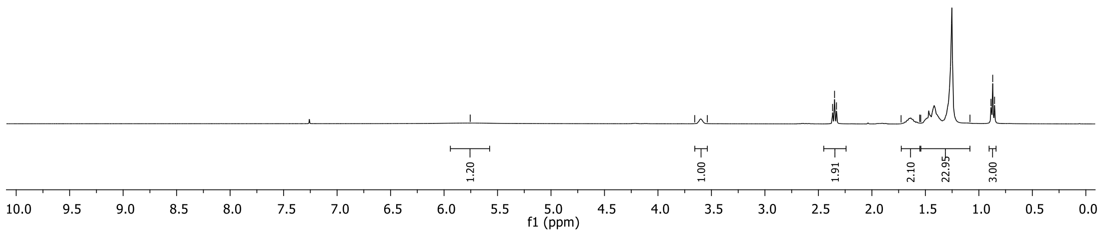
5.76
3.66
3.54
2.37
2.35
2.33
1.73
1.55
1.54
1.08
0.89
0.87
0.85
1.20
1.00
1.91
2.10
22.95
3.00
10.0 9.5 9.0 8.5 8.0 7.5 7.0 6.5 6.0 5.5 5.0 4.5 4.0 3.5 3.0 2.5 2.0 1.5 1.0 0.5 0.0
f1 (ppm)

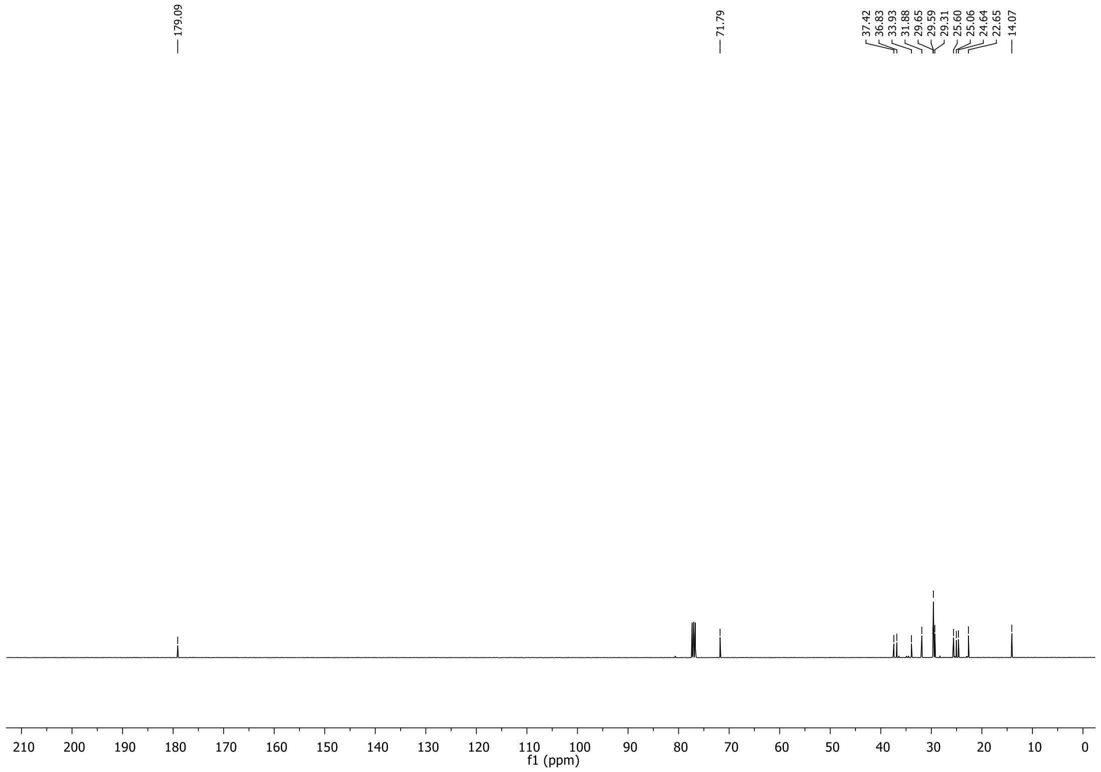
179.09
71.79
37.42
36.83
33.93
31.88
29.65
29.59
29.31
25.60
25.06
24.64
22.65
14.07
210 200 190 180 170 160 150 140 130 120 110 100 90 80 70 60 50 40 30 20 10 0
f1 (ppm)

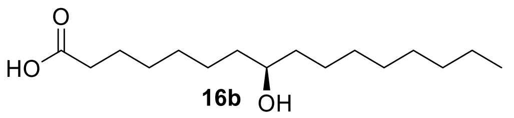
O
HO
OH

16b

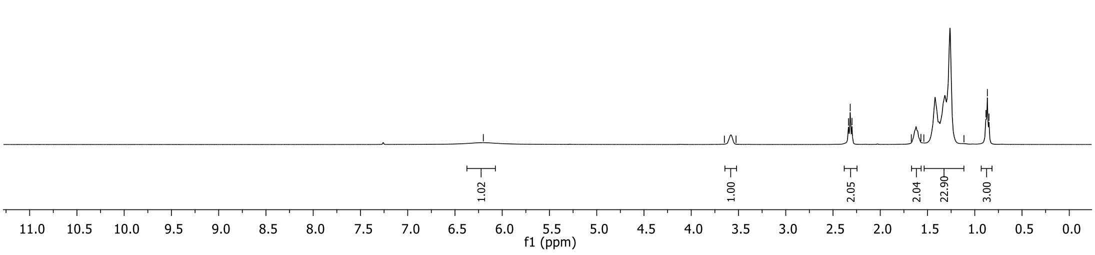
6.20
3.65
3.53
2.34
2.32
2.30
1.67
1.57
1.54
1.11
0.88
0.87
0.85
1.02
1.00
2.05
2.04
22.90
3.00
11.0 10.5 10.0 9.5 9.0 8.5 8.0 7.5 7.0 6.5 6.0 5.5 5.0 4.5 4.0 3.5 3.0 2.5 2.0 1.5 1.0 0.5 0.0
f1 (ppm)

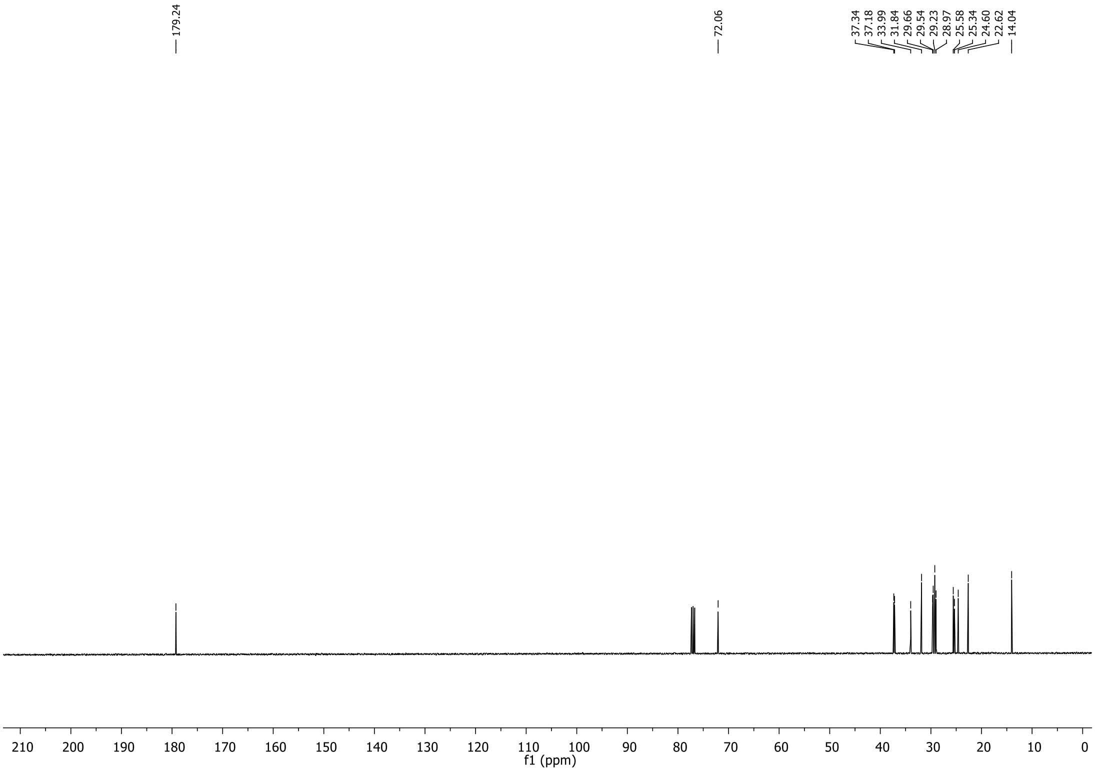
179.24
72.06
37.34
37.18
33.99
31.84
29.66
29.54
29.23
28.97
25.58
25.34
24.60
22.62
14.04
210 200 190 180 170 160 150 140 130 120 110 100 90 80 70 60 50 40 30 20 10 0
f1 (ppm)

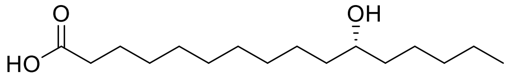
O
OH
HO

16c

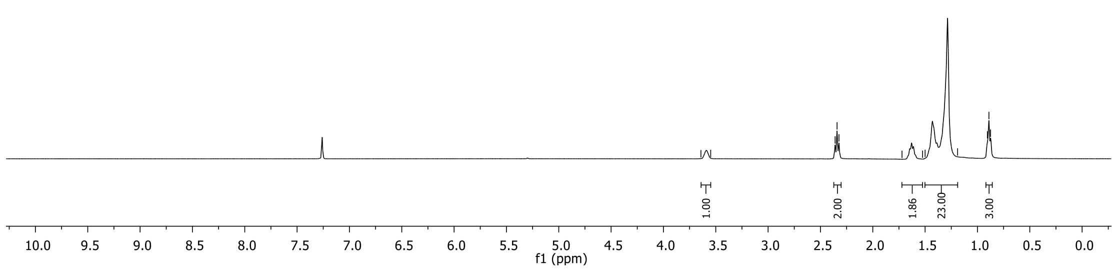
3.64
3.55
2.36
2.34
2.32
1.72
1.52
1.50
1.19
0.91
0.89
0.87
1.00
2.00
1.86
23.00
3.00
10.0 9.5 9.0 8.5 8.0 7.5 7.0 6.5 6.0 5.5 5.0 4.5 4.0 3.5 3.0 2.5 2.0 1.5 1.0 0.5 0.0
f1 (ppm)

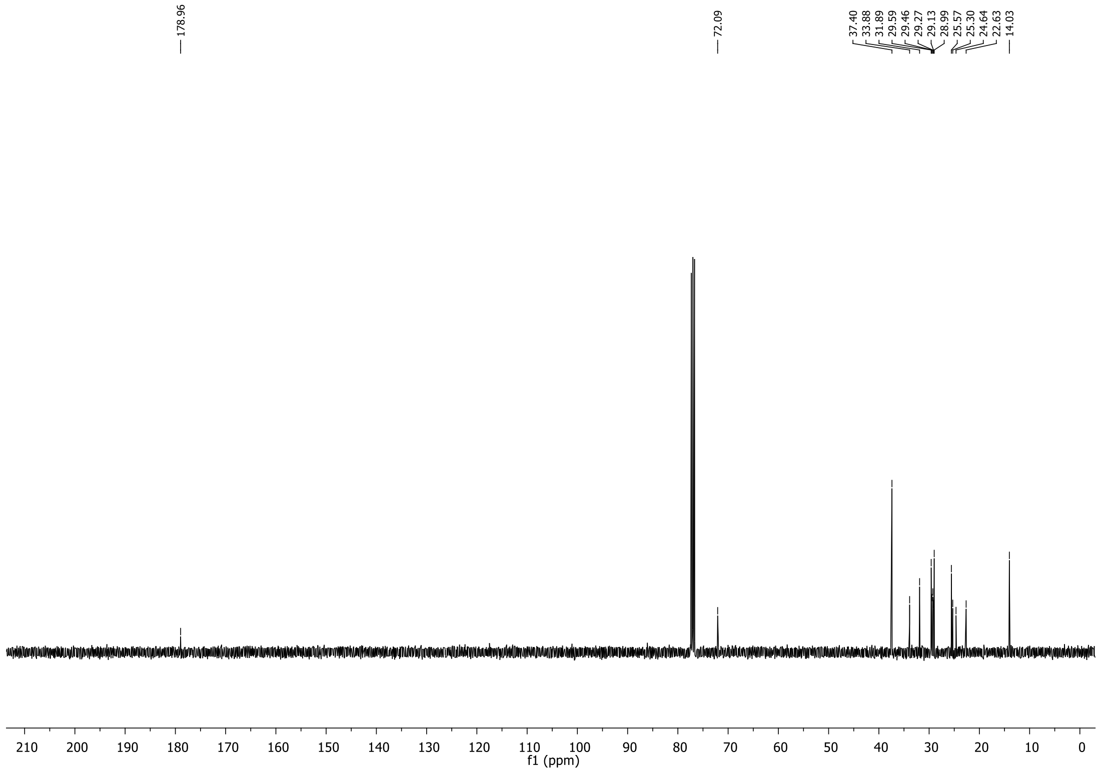
178.96
72.09
37.40
33.88
31.89
29.59
29.46
29.27
29.13
28.99
25.57
25.30
24.64
22.63
14.03
210 200 190 180 170 160 150 140 130 120 110 100 90 80 70 60 50 40 30 20 10 0
f1 (ppm)

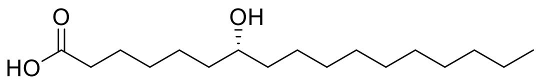
OH
HO
O

16d

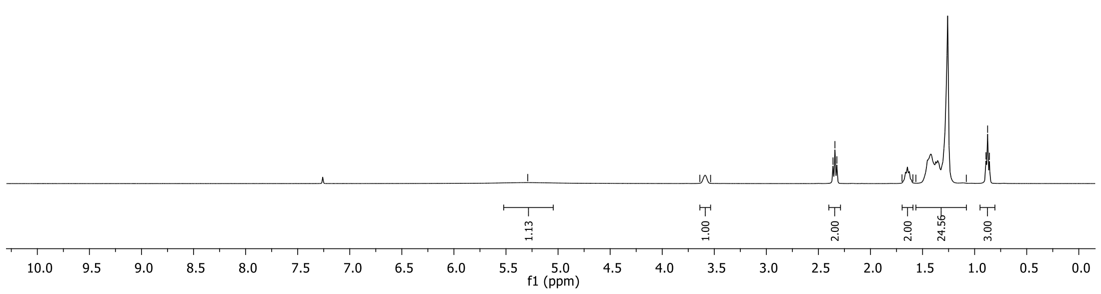
5.29
3.64
3.54
2.36
2.34
2.32
1.70
1.59
1.56
1.08
0.89
0.88
0.86
1.13
1.00
2.00
2.00
24.56
3.00
f1 (ppm)

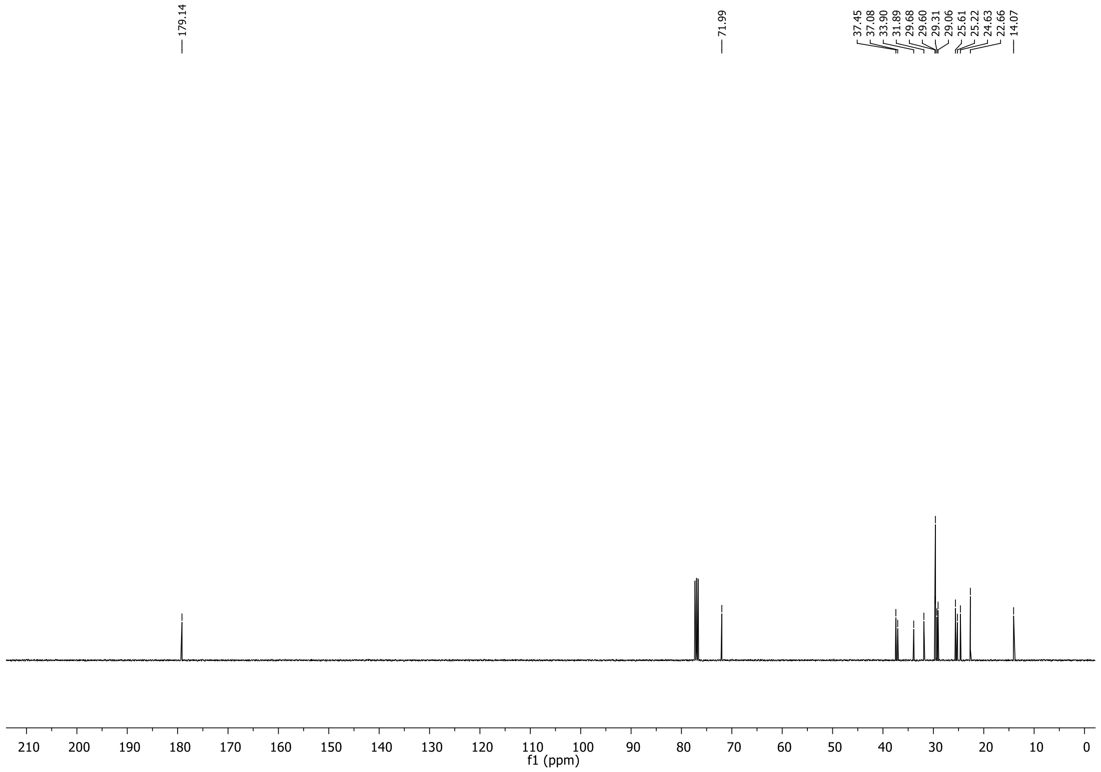
179.14
71.99
37.45
37.08
33.90
31.89
29.68
29.60
29.31
29.06
25.61
25.22
24.63
22.66
14.07
f1 (ppm)

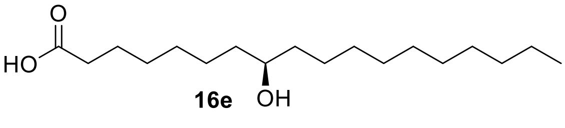

16e

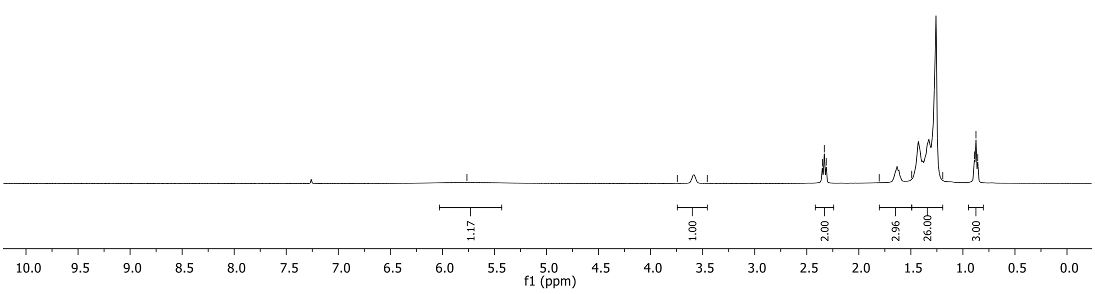
5.76
3.74
3.46
2.35
2.33
2.31
1.80
1.49
1.19
0.89
0.87
0.86
1.17
1.00
2.00
2.96
26.00
3.00
f1 (ppm)

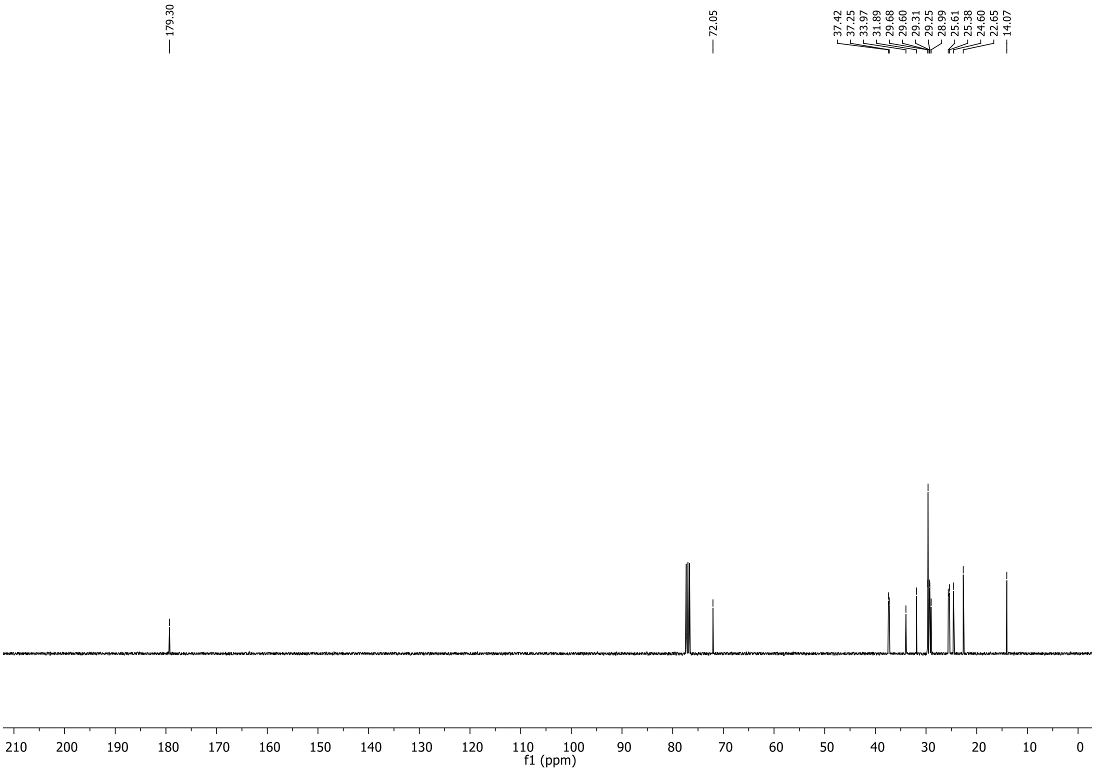
179.30
72.05
37.42
37.25
33.97
31.89
29.68
29.60
29.31
29.25
28.99
25.61
25.38
24.60
22.65
14.07
f1 (ppm)

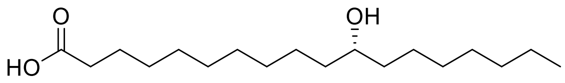
O
OH
HO

16f

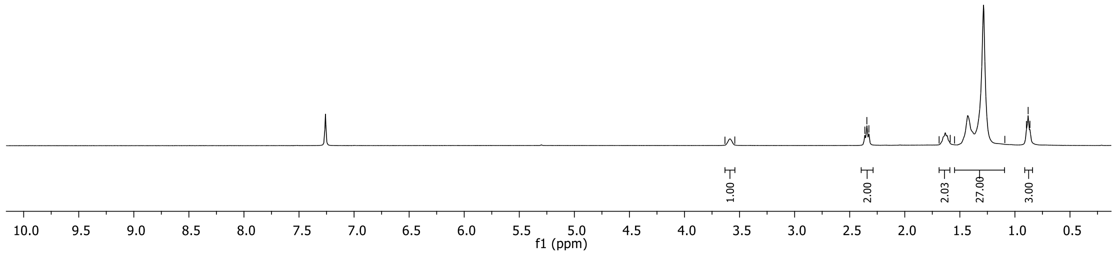
3.63
3.54
2.36
2.34
2.33
1.69
1.59
1.55
1.09
0.90
0.88
0.86
1.00
2.00
2.03
27.00
3.00
10.0 9.5 9.0 8.5 8.0 7.5 7.0 6.5 6.0 5.5 5.0 4.5 4.0 3.5 3.0 2.5 2.0 1.5 1.0 0.5
f1 (ppm)

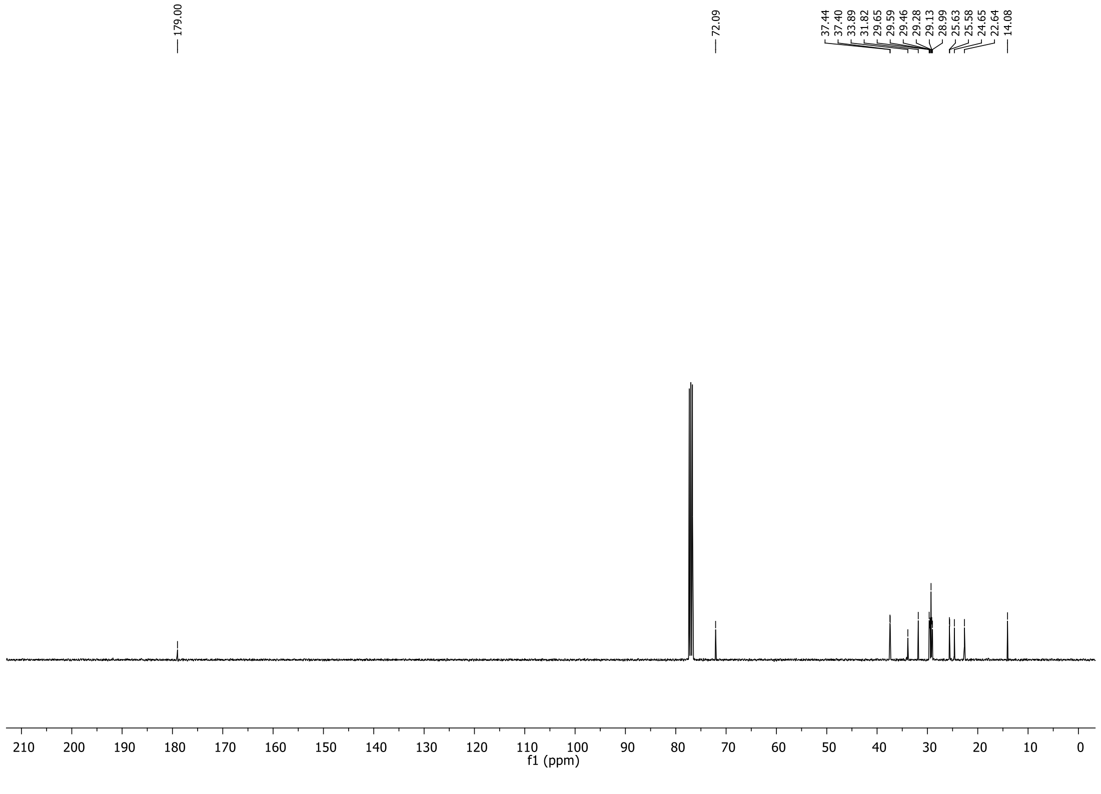
179.00
72.09
37.44
37.40
33.89
31.82
29.65
29.59
29.46
29.28
29.13
28.99
25.63
25.58
24.65
22.64
14.08
210 200 190 180 170 160 150 140 130 120 110 100 90 80 70 60 50 40 30 20 10 0
f1 (ppm)

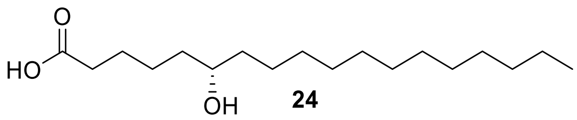
O
HO
OH

24

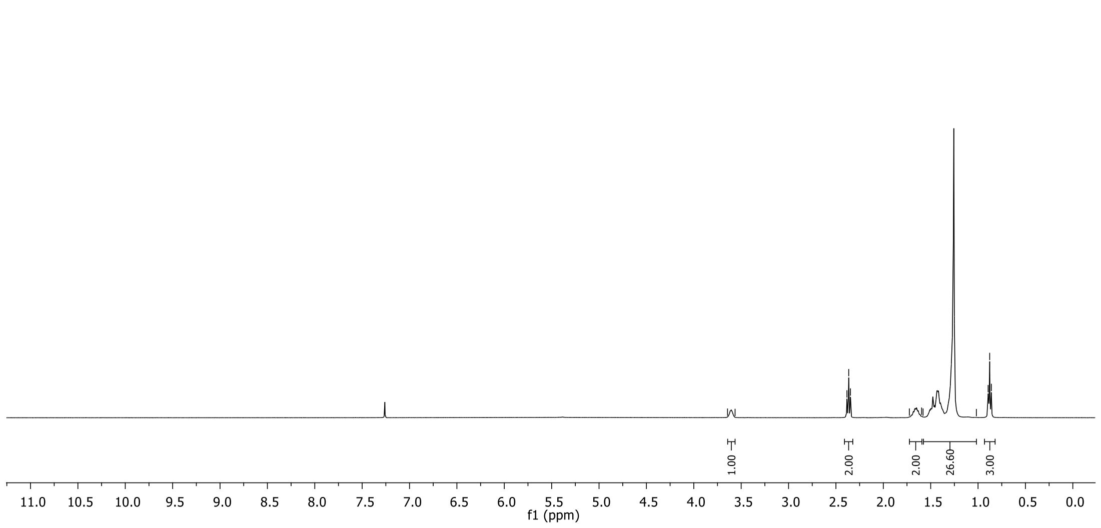
3.64
3.56
2.38
2.37
2.35
1.73
1.60
1.58
1.02
0.89
0.88
0.86
1.00
2.00
2.00
26.60
3.00
11.0 10.5 10.0 9.5 9.0 8.5 8.0 7.5 7.0 6.5 6.0 5.5 5.0 4.5 4.0 3.5 3.0 2.5 2.0 1.5 1.0 0.5 0.0
f1 (ppm)

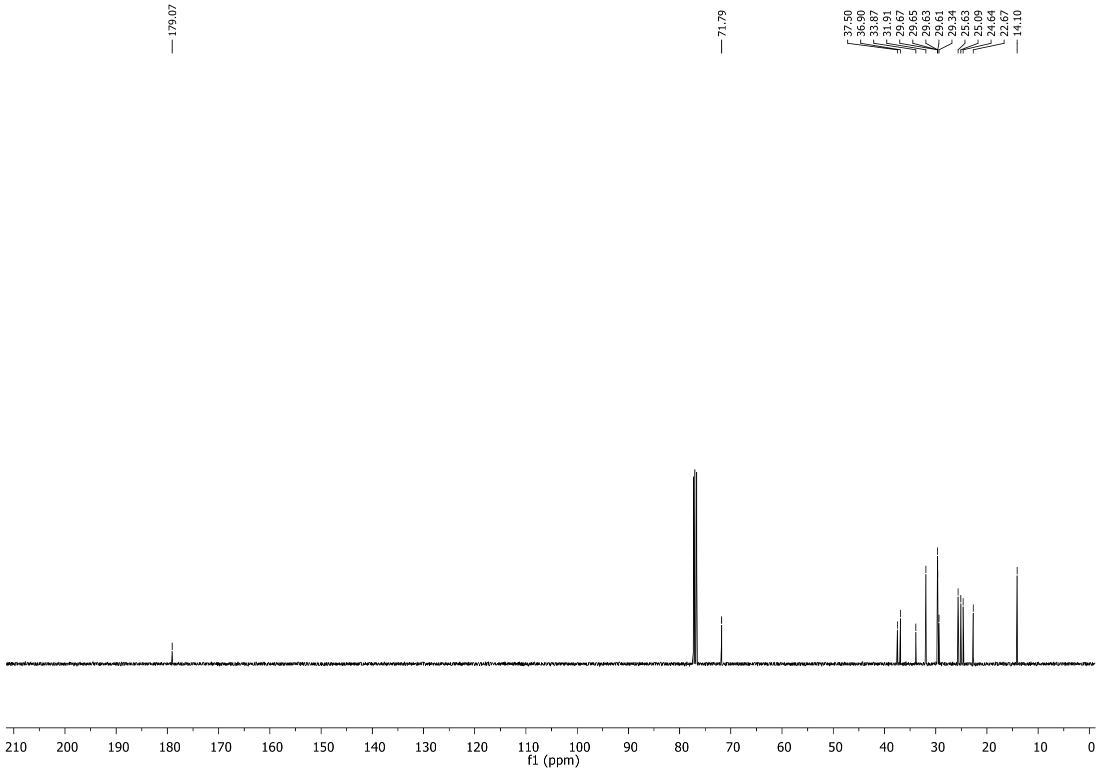
179.07
71.79
37.50
36.90
33.87
31.91
29.67
29.65
29.63
29.61
29.34
25.63
25.09
24.64
22.67
14.10
210 200 190 180 170 160 150 140 130 120 110 100 90 80 70 60 50 40 30 20 10 0
f1 (ppm)

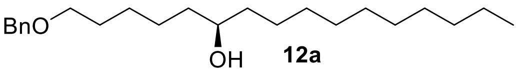
BnO
OH

12a

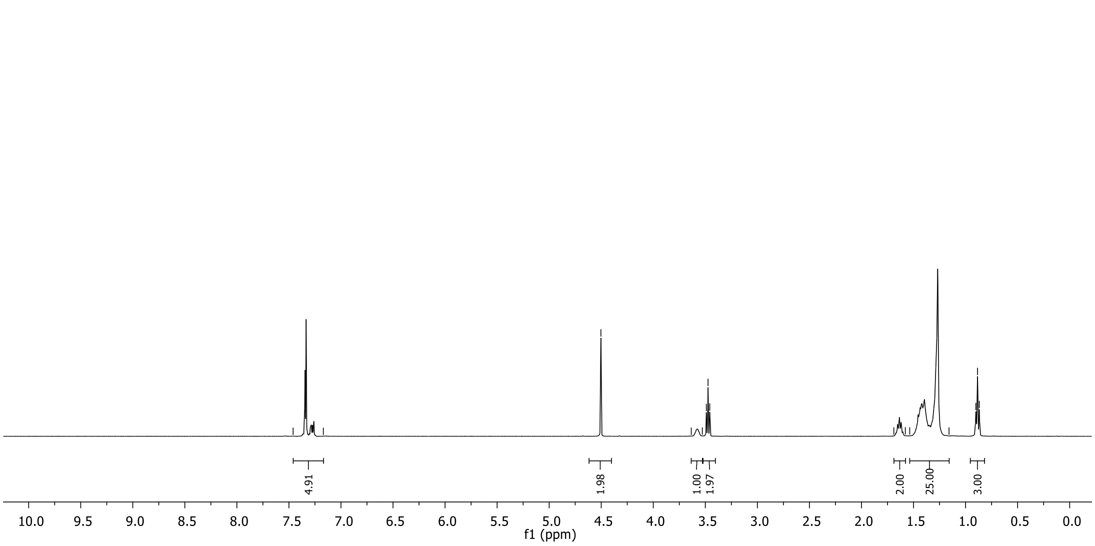
7.46
7.17
4.50
3.63
3.53
3.49
3.47
3.46
1.69
1.58
1.54
1.16
0.90
0.89
0.87
4.91
1.98
1.00
1.97
2.00
25.00
3.00
10.0 9.5 9.0 8.5 8.0 7.5 7.0 6.5 6.0 5.5 5.0 4.5 4.0 3.5 3.0 2.5 2.0 1.5 1.0 0.5 0.0
f1 (ppm)

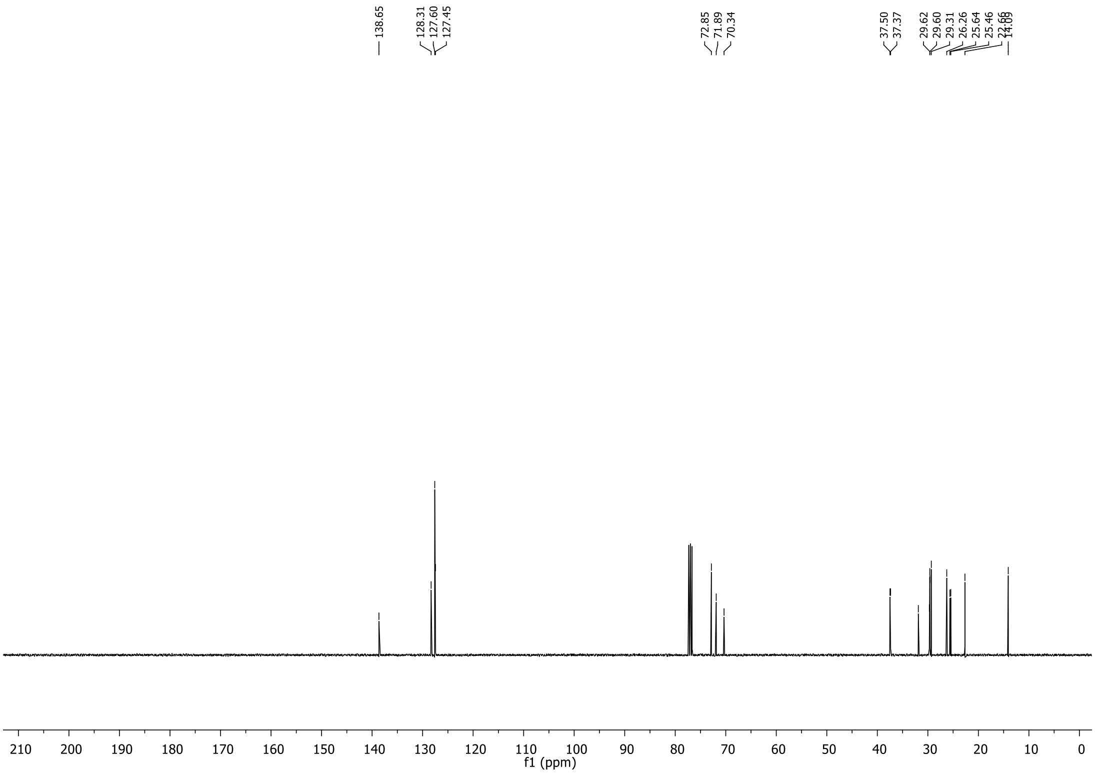
138.65
128.31
127.60
127.45
72.85
71.89
70.34
37.50
37.37
29.62
29.60
29.31
26.26
25.64
25.46
210 200 190 180 170 160 150 140 130 120 110 100 90 80 70 60 50 40 30 20 10 0
f1 (ppm)

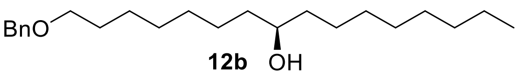
BnO
OH

12b

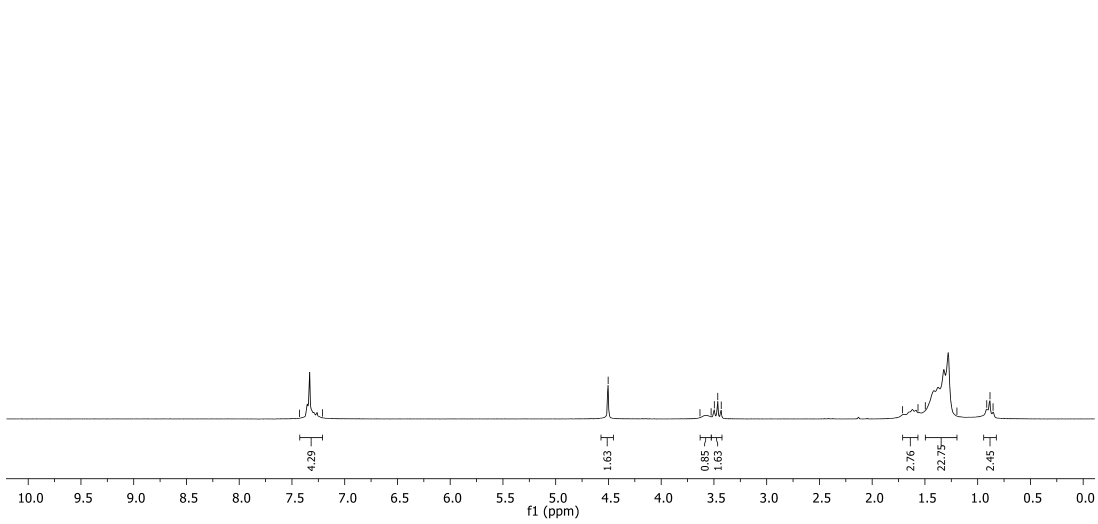
7.43
7.21
4.50
3.63
3.53
3.50
3.46
3.43
1.71
1.57
1.50
1.20
0.91
0.88
0.85
4.29
1.63
0.85
1.63
2.76
22.75
2.45
10.0 9.5 9.0 8.5 8.0 7.5 7.0 6.5 6.0 5.5 5.0 4.5 4.0 3.5 3.0 2.5 2.0 1.5 1.0 0.5 0.0
f1 (ppm)

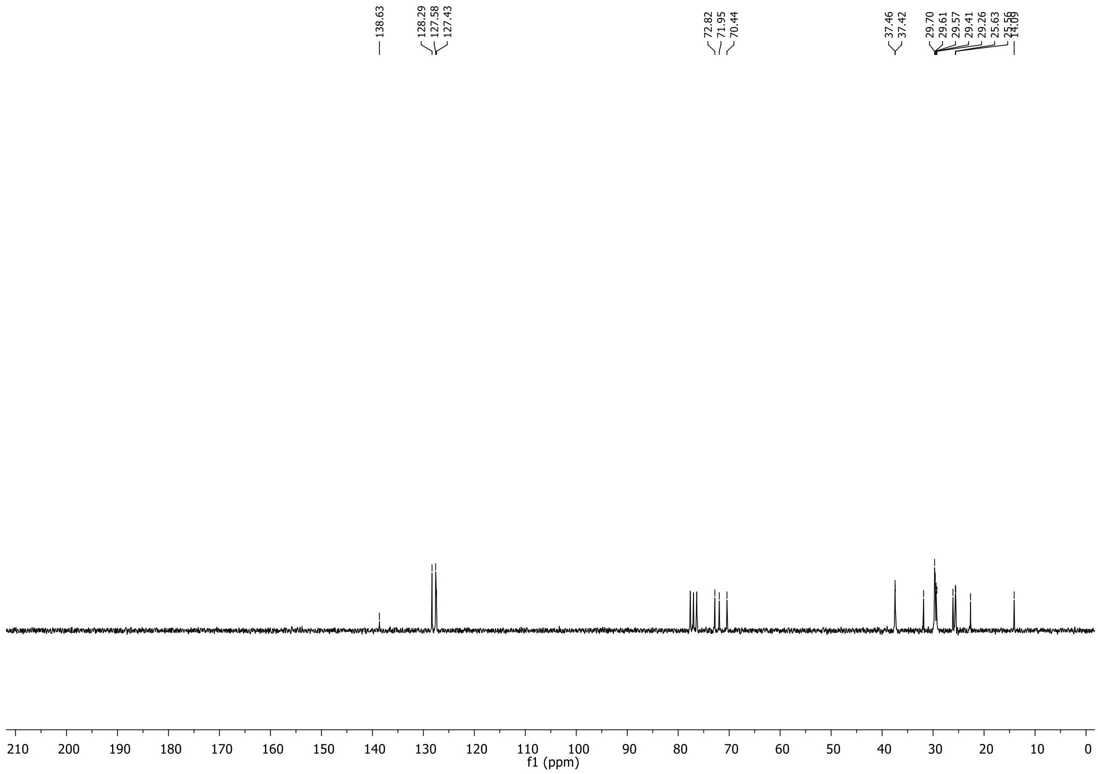
138.63
128.29
127.58
127.43
72.82
71.95
70.44
37.46
37.42
29.70
29.61
29.57
29.41
29.26
25.63
210 200 190 180 170 160 150 140 130 120 110 100 90 80 70 60 50 40 30 20 10 0
f1 (ppm)

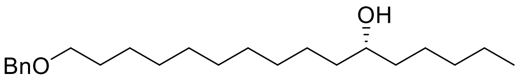

12c

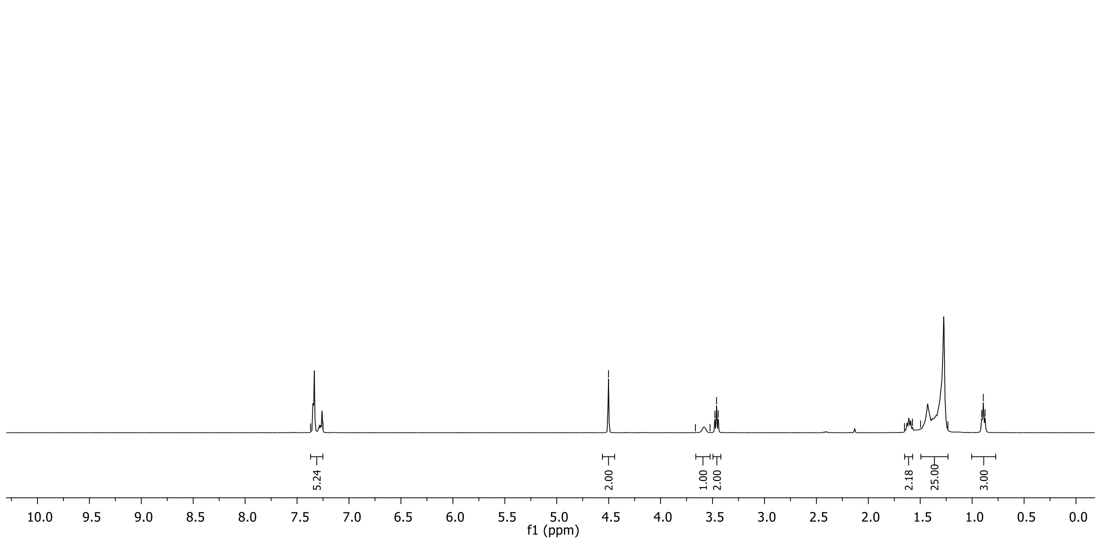

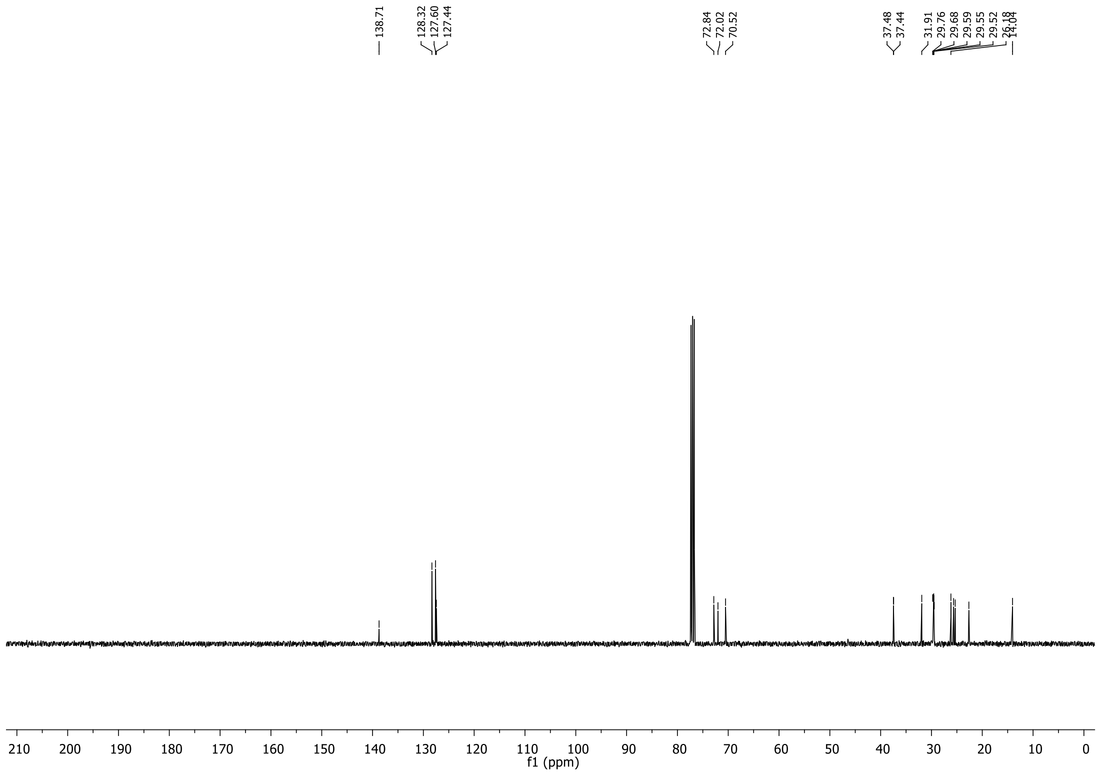

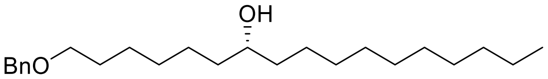
OH
BnO

12d

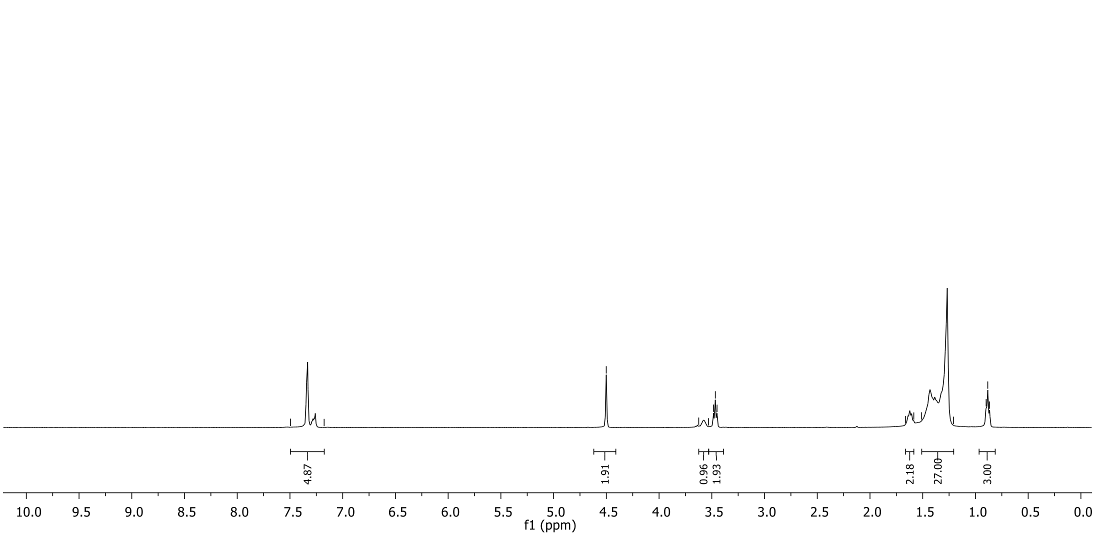
7.50
7.18
4.50
3.62
3.53
3.48
3.47
3.45
1.66
1.58
1.51
1.21
0.90
0.88
0.87
4.87
1.91
0.96
1.93
2.18
27.00
3.00
10.0 9.5 9.0 8.5 8.0 7.5 7.0 6.5 6.0 5.5 5.0 4.5 4.0 3.5 3.0 2.5 2.0 1.5 1.0 0.5 0.0
f1 (ppm)

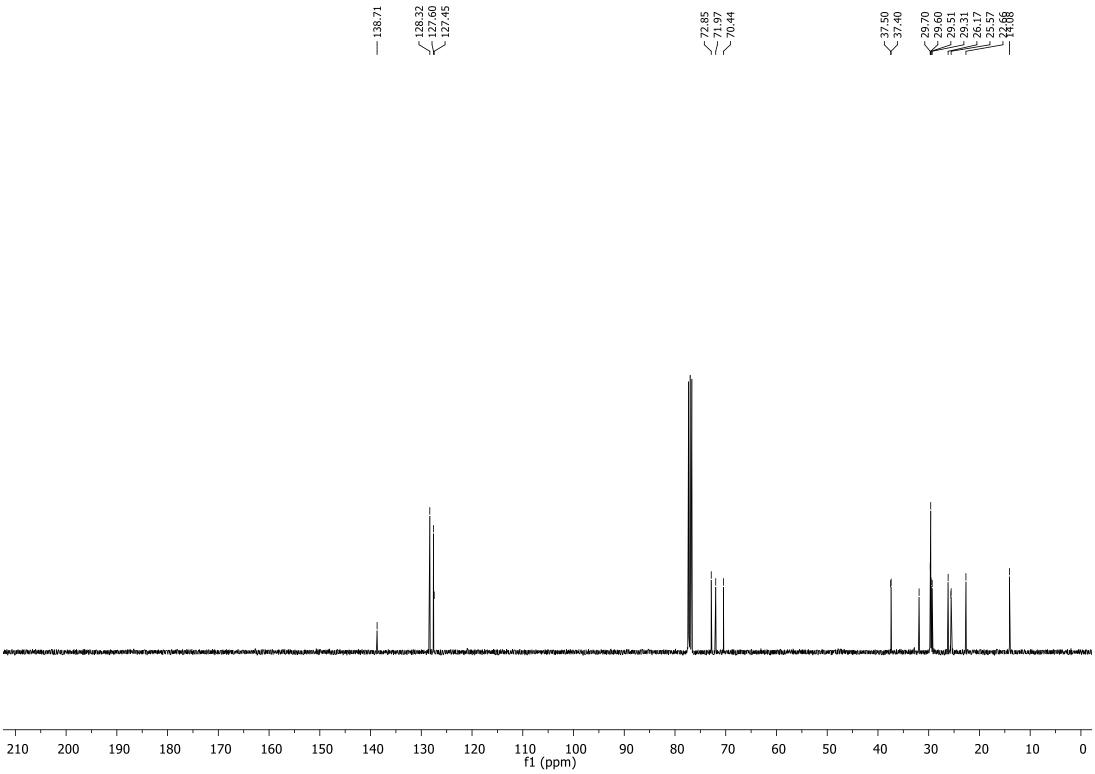
138.71
128.32
127.60
127.45
72.85
71.97
70.44
37.50
37.40
29.70
29.60
29.51
29.31
26.17
25.57
14.08
210 200 190 180 170 160 150 140 130 120 110 100 90 80 70 60 50 40 30 20 10 0
f1 (ppm)

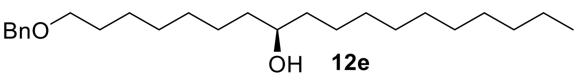
BnO
OH

12e

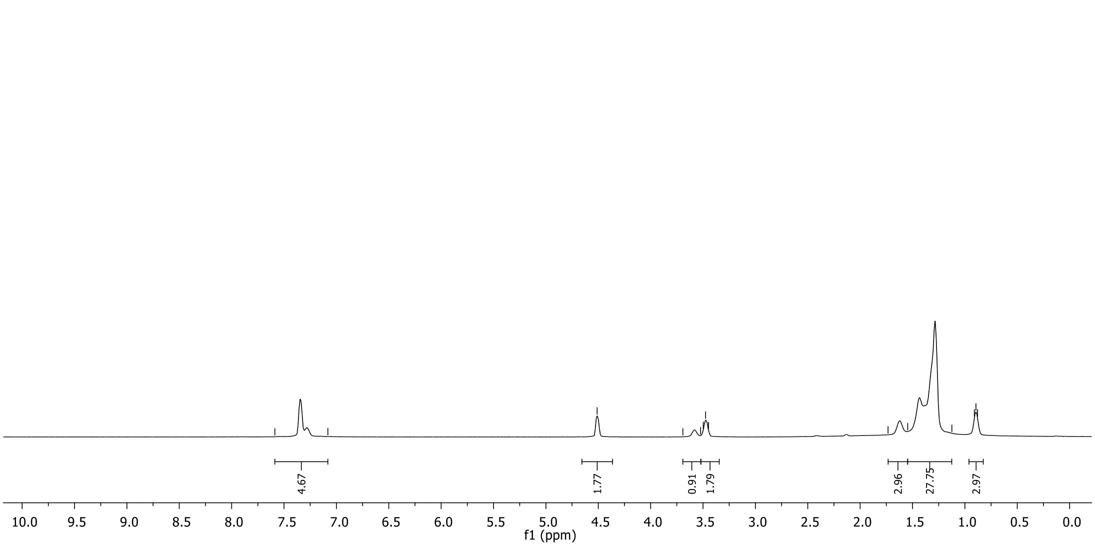
7.59
7.08
4.51
3.69
3.52
3.50
3.48
3.45
1.73
1.55
1.12
0.91
0.89
0.88
4.67
1.77
0.91
1.79
2.96
27.75
2.97
10.0 9.5 9.0 8.5 8.0 7.5 7.0 6.5 6.0 5.5 5.0 4.5 4.0 3.5 3.0 2.5 2.0 1.5 1.0 0.5 0.0
f1 (ppm)

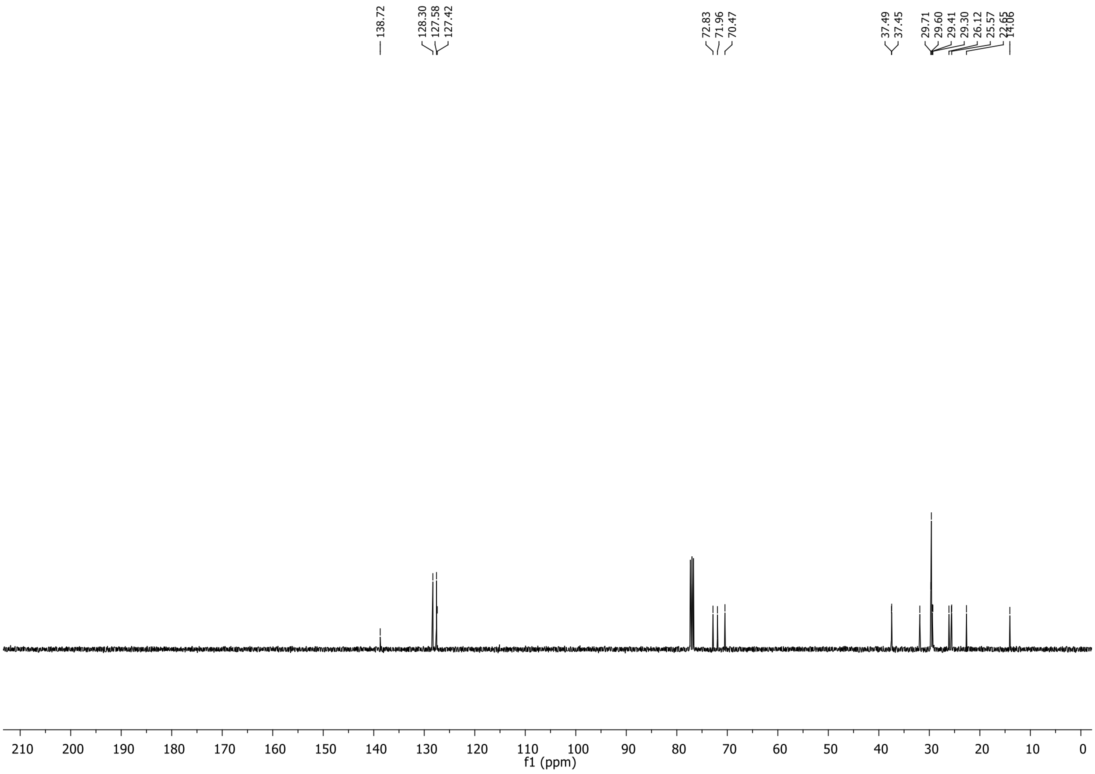
138.72
128.30
127.58
127.42
72.83
71.96
70.47
37.49
37.45
29.71
29.60
29.41
29.30
26.12
25.57
210 200 190 180 170 160 150 140 130 120 110 100 90 80 70 60 50 40 30 20 10 0
f1 (ppm)

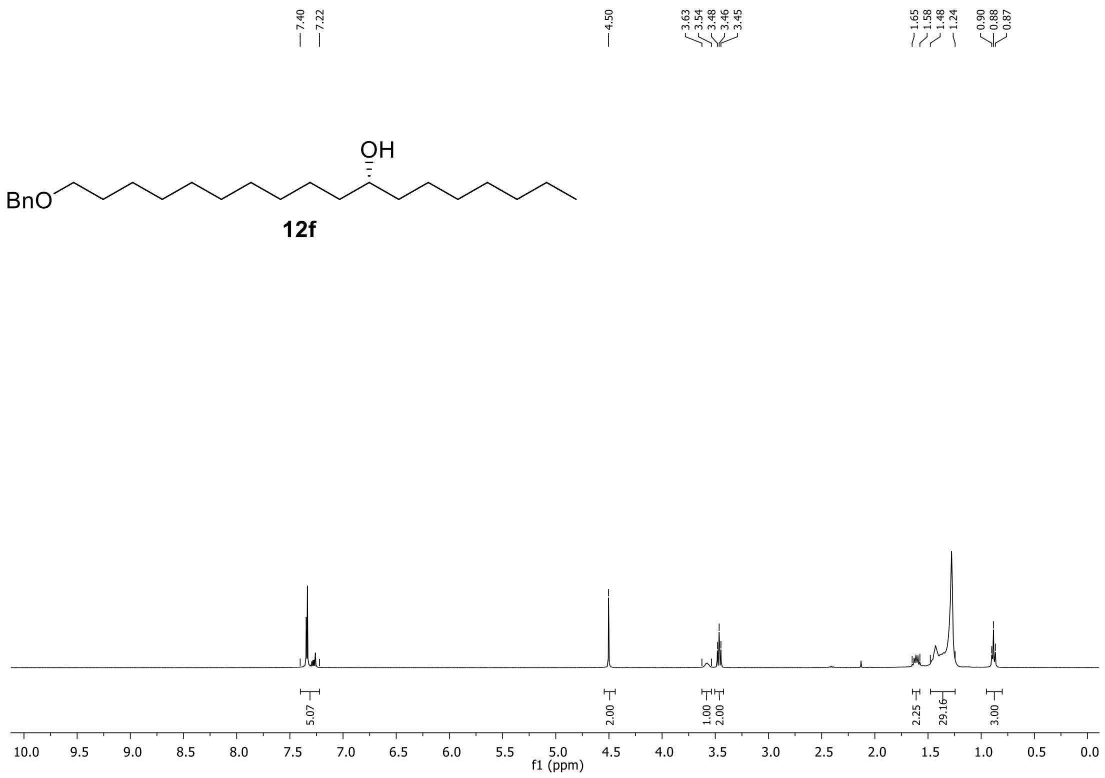

OH
BnO
12f
7.40
7.22
4.50
3.63
3.54
3.48
3.46
3.45
1.65
1.58
1.48
1.24
0.90
0.88
0.87
5.07
2.00
1.00
2.00
2.25
29.16
3.00
f1 (ppm)

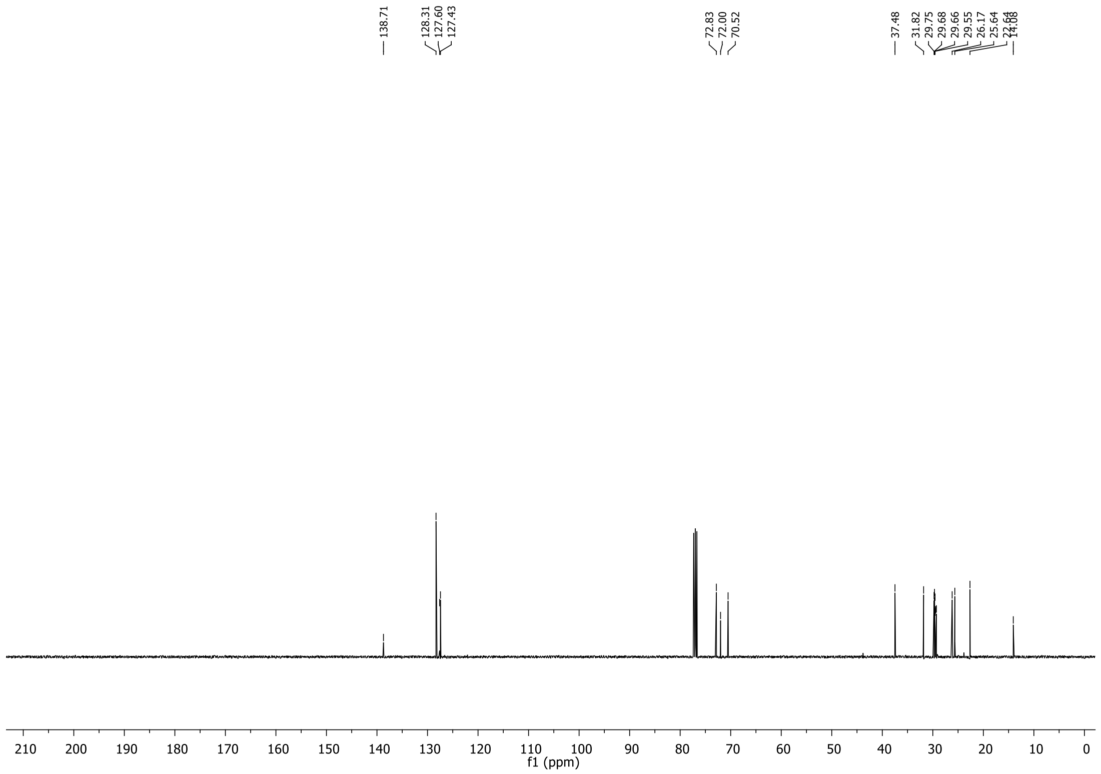

138.71
128.31
127.60
127.43
72.83
72.00
70.52
37.48
31.82
29.75
29.68
29.66
29.55
26.17
25.64
14.08
f1 (ppm)

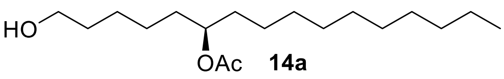
HO
OAc

14a

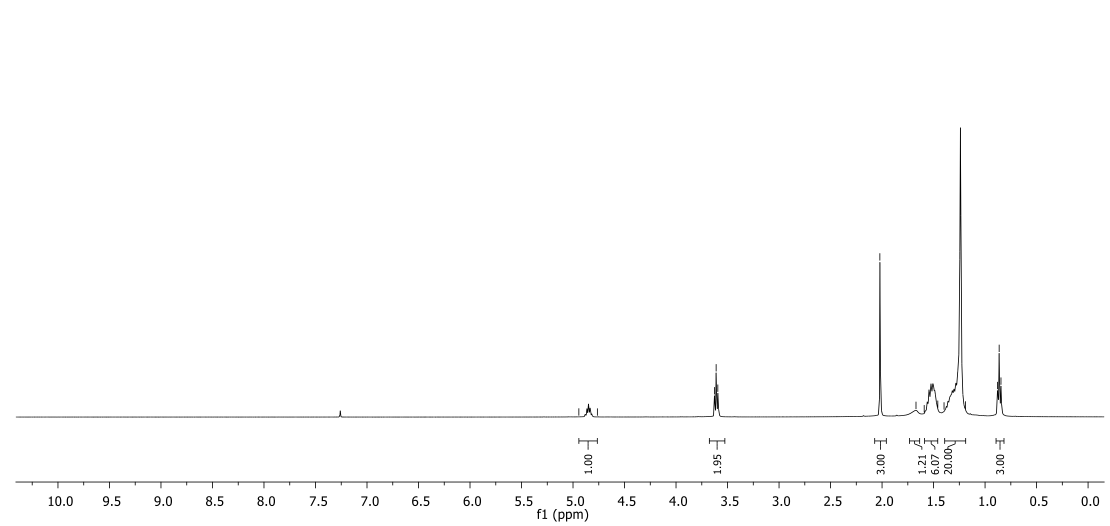
4.94
4.76
3.63
3.61
3.59
2.02
1.67
1.59
1.46
1.40
1.19
0.88
0.86
0.85
1.00
1.95
3.00
1.21
6.07
20.00
3.00
10.0 9.5 9.0 8.5 8.0 7.5 7.0 6.5 6.0 5.5 5.0 4.5 4.0 3.5 3.0 2.5 2.0 1.5 1.0 0.5 0.0
f1 (ppm)

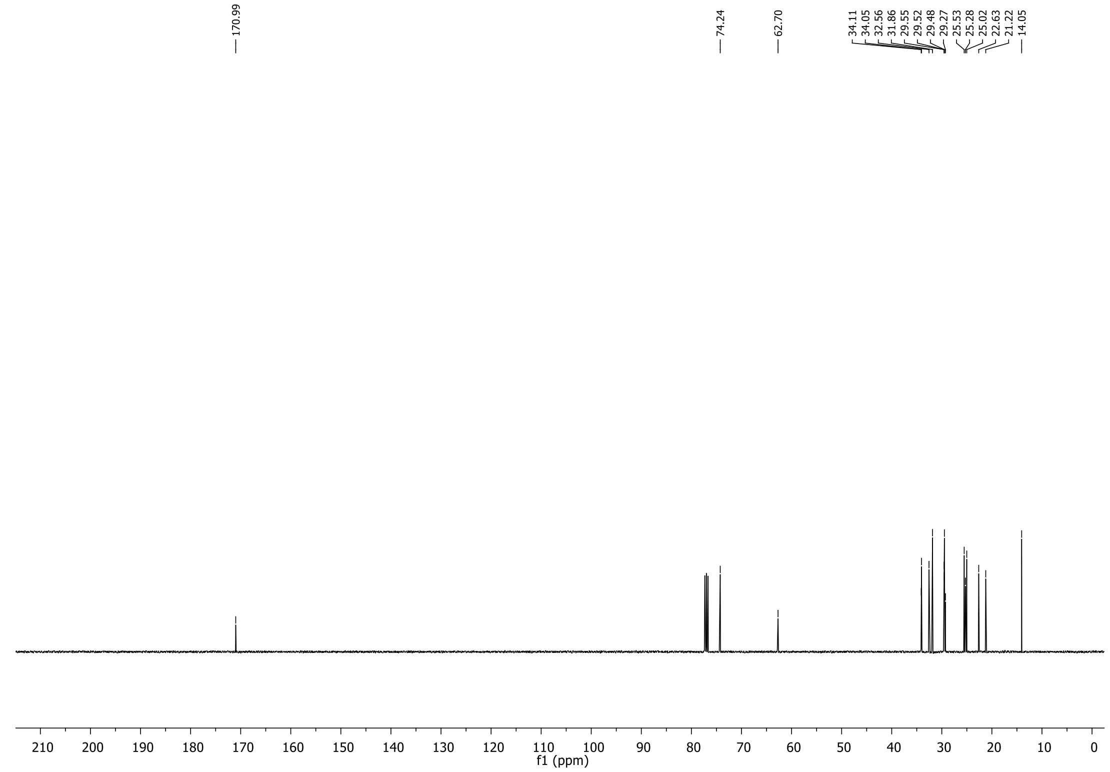
170.99
74.24
62.70
34.11
34.05
32.56
31.86
29.55
29.52
29.48
29.27
25.53
25.28
25.02
22.63
21.22
14.05
210 200 190 180 170 160 150 140 130 120 110 100 90 80 70 60 50 40 30 20 10 0
f1 (ppm)

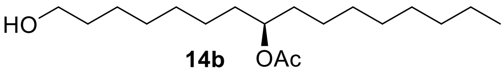
HO
OAc

14b

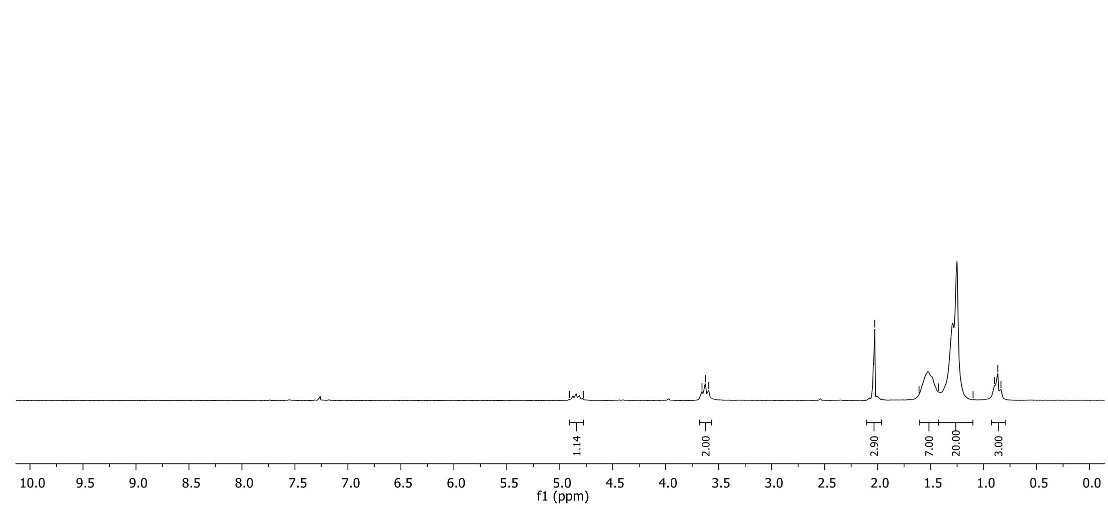
4.91
4.78
3.66
3.63
3.59
2.03
1.61
1.43
1.10
0.90
0.87
0.84
1.14
2.00
2.90
7.00
20.00
3.00
10.0 9.5 9.0 8.5 8.0 7.5 7.0 6.5 6.0 5.5 5.0 4.5 4.0 3.5 3.0 2.5 2.0 1.5 1.0 0.5 0.0
f1 (ppm)

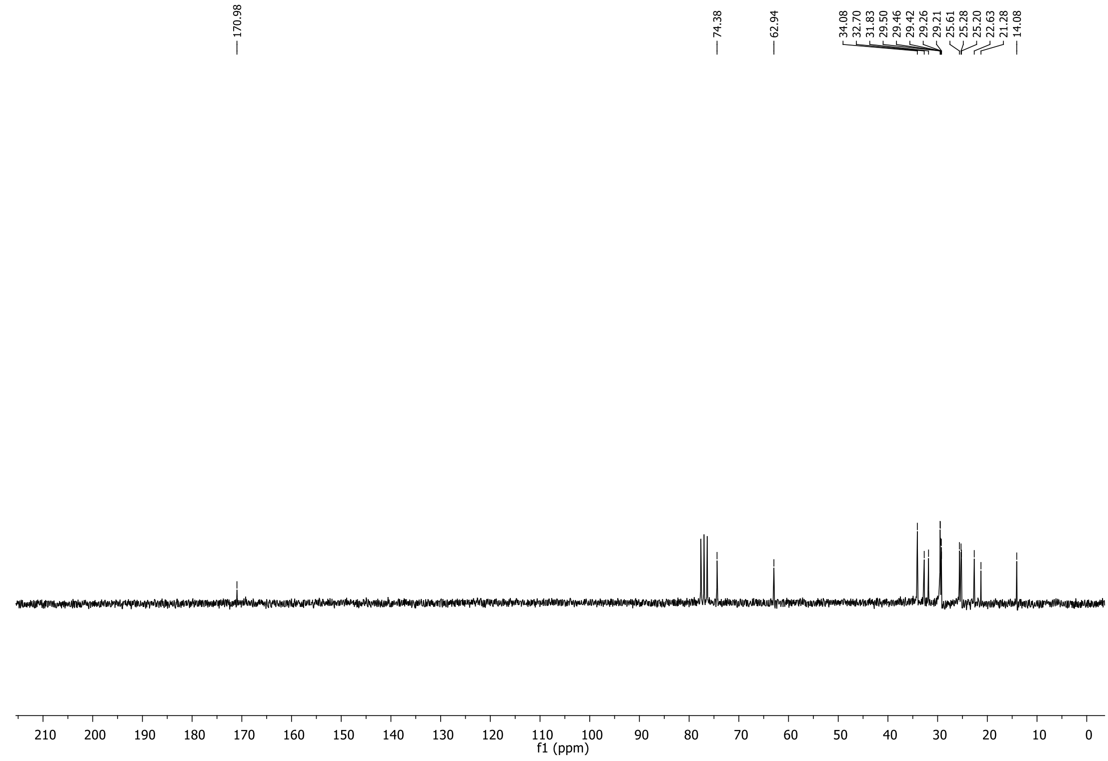
170.98
74.38
62.94
34.08
32.70
31.83
29.50
29.46
29.42
29.26
29.21
25.61
25.28
25.20
22.63
21.28
14.08
210 200 190 180 170 160 150 140 130 120 110 100 90 80 70 60 50 40 30 20 10 0
f1 (ppm)

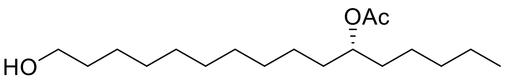
OAc
HO

14c

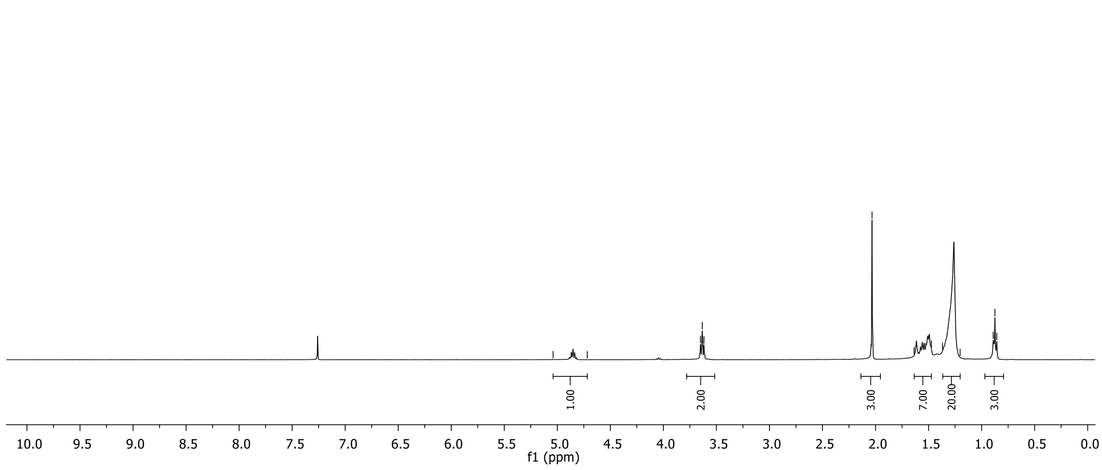
5.04
4.72
3.65
3.63
3.62
2.03
1.64
1.47
1.37
1.20
0.89
0.87
0.86
1.00
2.00
3.00
7.00
20.00
3.00
f1 (ppm)

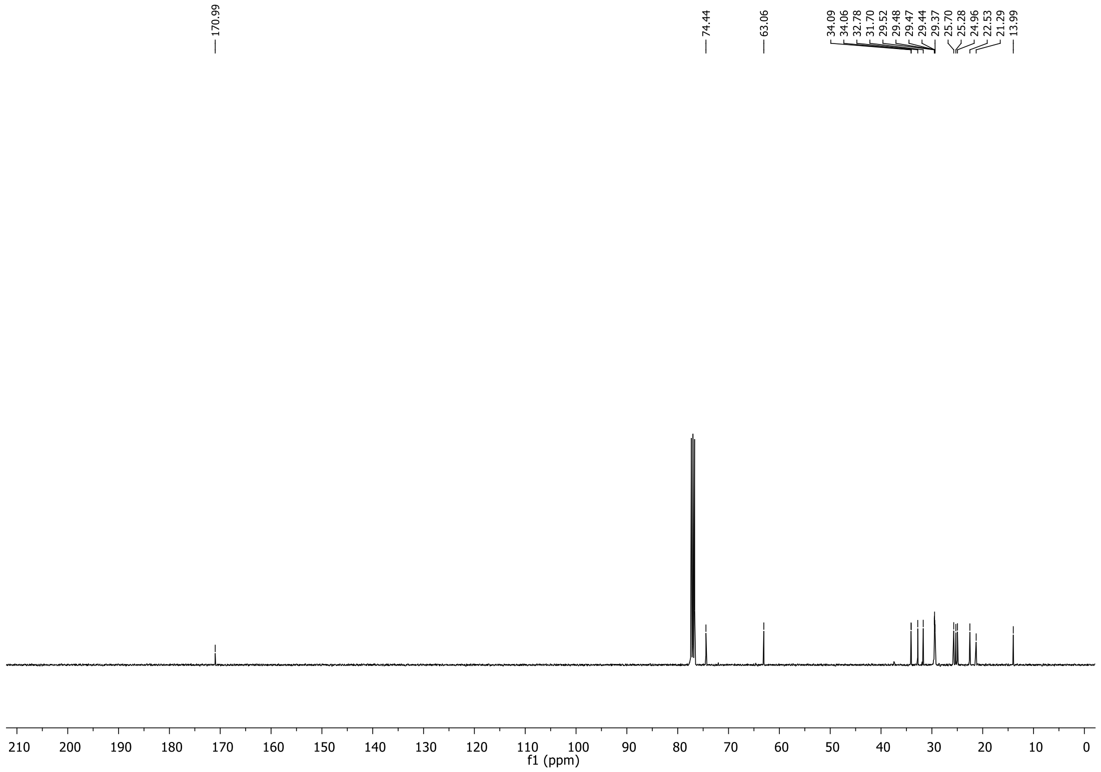
170.99
74.44
63.06
34.09
34.06
32.78
31.70
29.52
29.48
29.47
29.44
29.37
25.70
25.28
24.96
22.53
21.29
13.99
f1 (ppm)

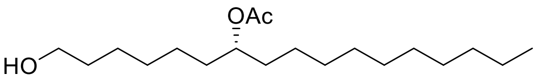
OAc
HO

14d

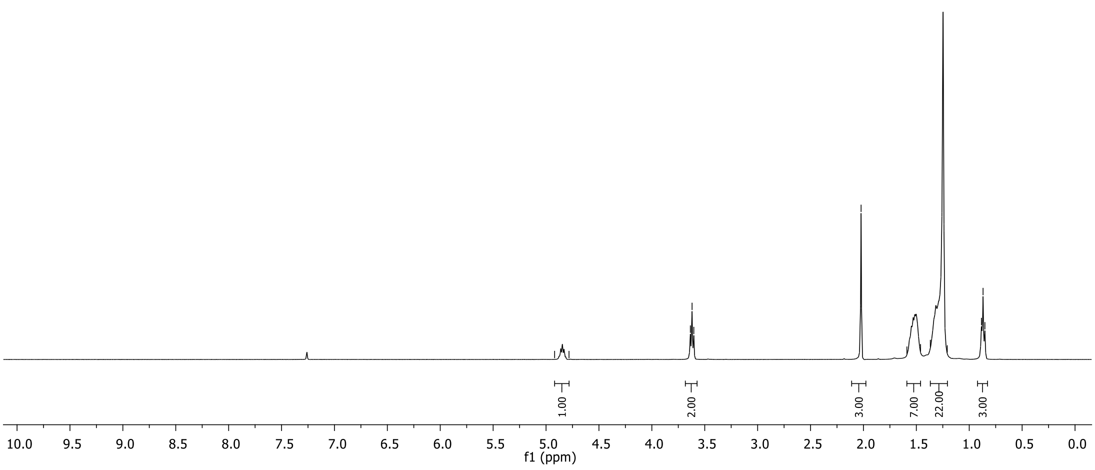
4.92
4.78
3.64
3.62
3.60
2.02
1.59
1.46
1.37
1.21
0.88
0.87
0.85
1.00
2.00
3.00
7.00
22.00
3.00
10.0 9.5 9.0 8.5 8.0 7.5 7.0 6.5 6.0 5.5 5.0 4.5 4.0 3.5 3.0 2.5 2.0 1.5 1.0 0.5 0.0
f1 (ppm)

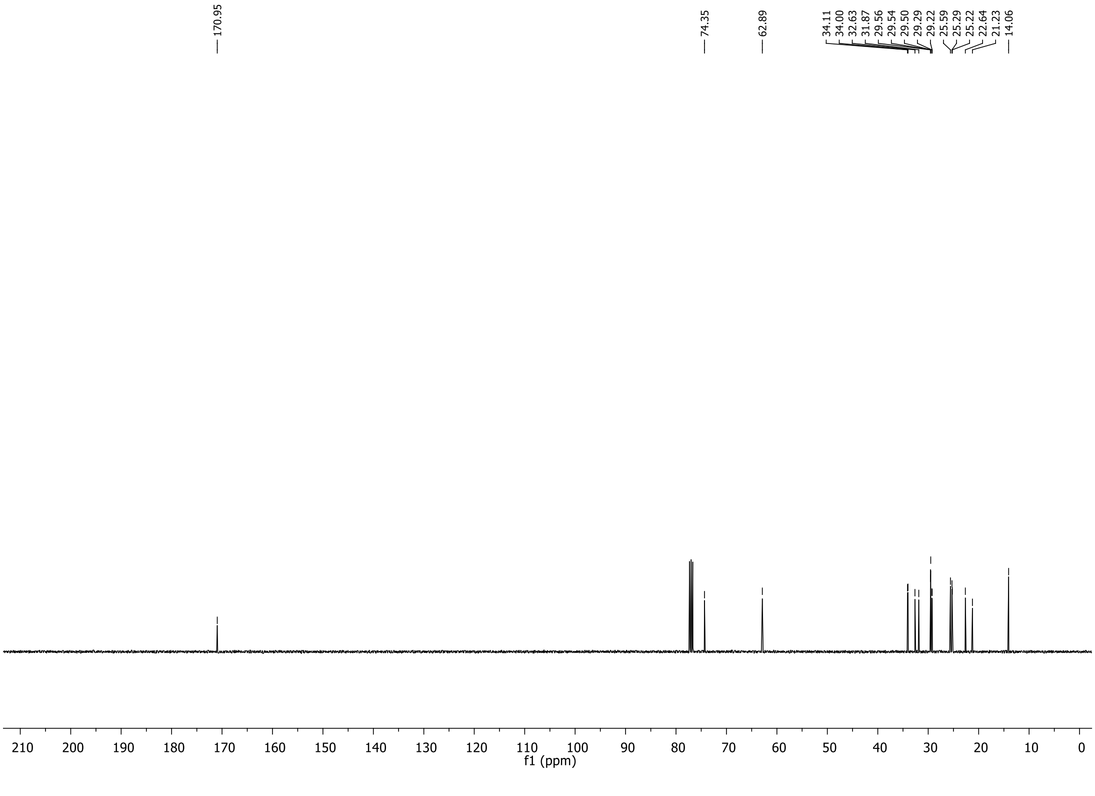
170.95
74.35
62.89
34.11
34.00
32.63
31.87
29.56
29.54
29.50
29.29
29.22
25.59
25.29
25.22
22.64
21.23
14.06
210 200 190 180 170 160 150 140 130 120 110 100 90 80 70 60 50 40 30 20 10 0
f1 (ppm)

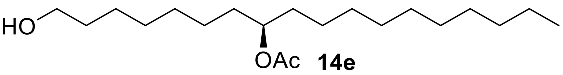
HO
OAc

14e

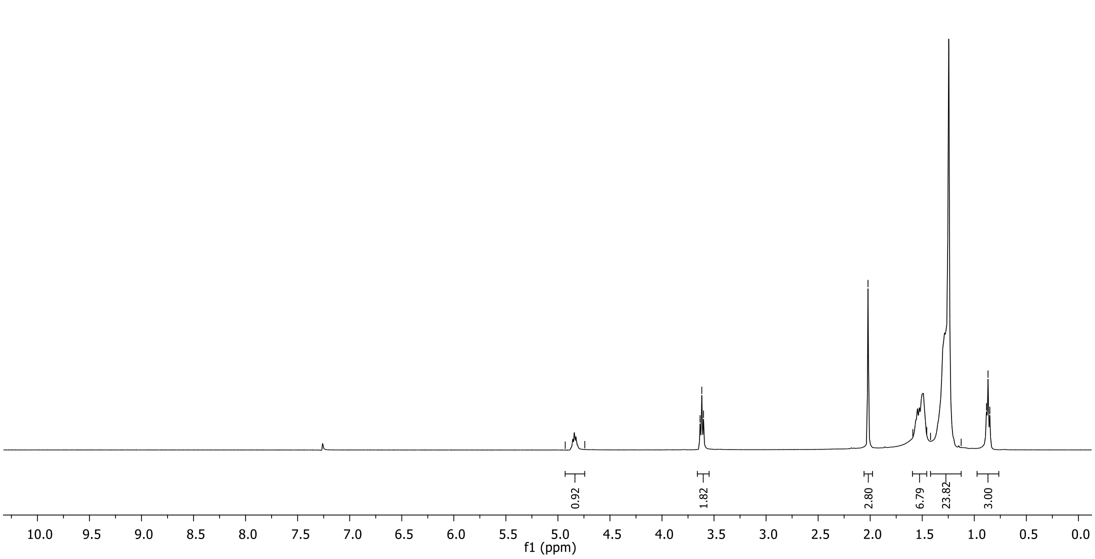
4.93
4.74
3.63
3.62
3.60
2.02
1.59
1.46
1.42
1.13
0.88
0.87
0.85
0.92
1.82
2.80
6.79
23.82
3.00
10.0 9.5 9.0 8.5 8.0 7.5 7.0 6.5 6.0 5.5 5.0 4.5 4.0 3.5 3.0 2.5 2.0 1.5 1.0 0.5 0.0
f1 (ppm)

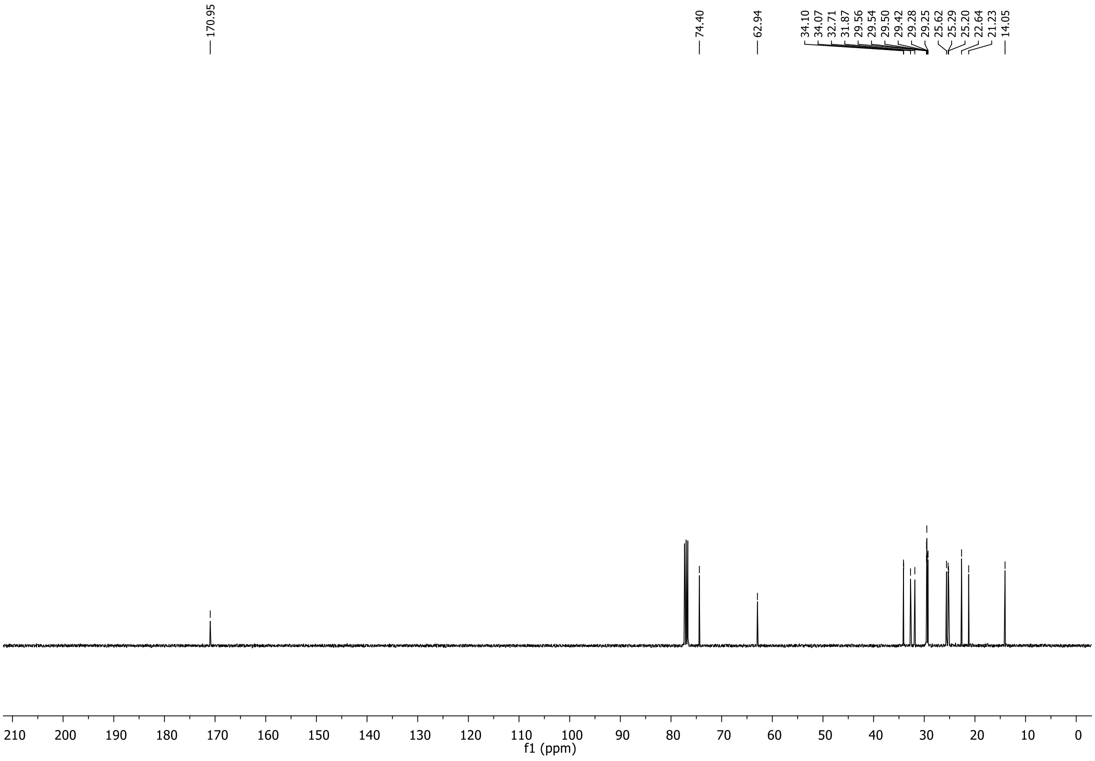
170.95
74.40
62.94
34.10
34.07
32.71
31.87
29.56
29.54
29.50
29.42
29.28
29.25
25.62
25.29
25.20
22.64
21.23
14.05
210 200 190 180 170 160 150 140 130 120 110 100 90 80 70 60 50 40 30 20 10 0
f1 (ppm)

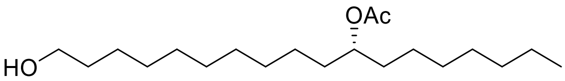
OAc
HO

14f

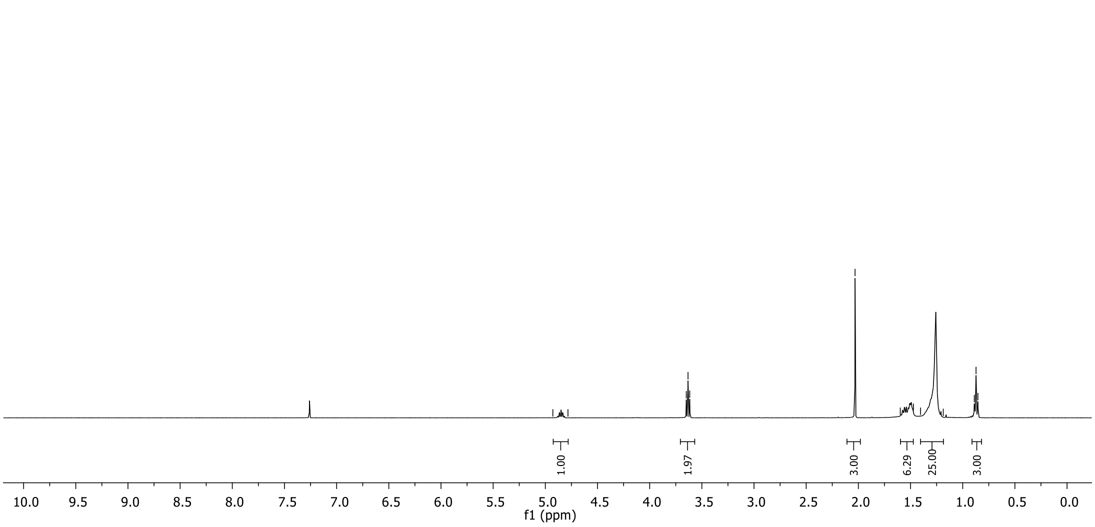
4.93
4.78
3.65
3.63
3.62
2.03
1.60
1.47
1.40
1.19
0.89
0.87
0.85
1.00
1.97
3.00
6.29
25.00
3.00
f1 (ppm)

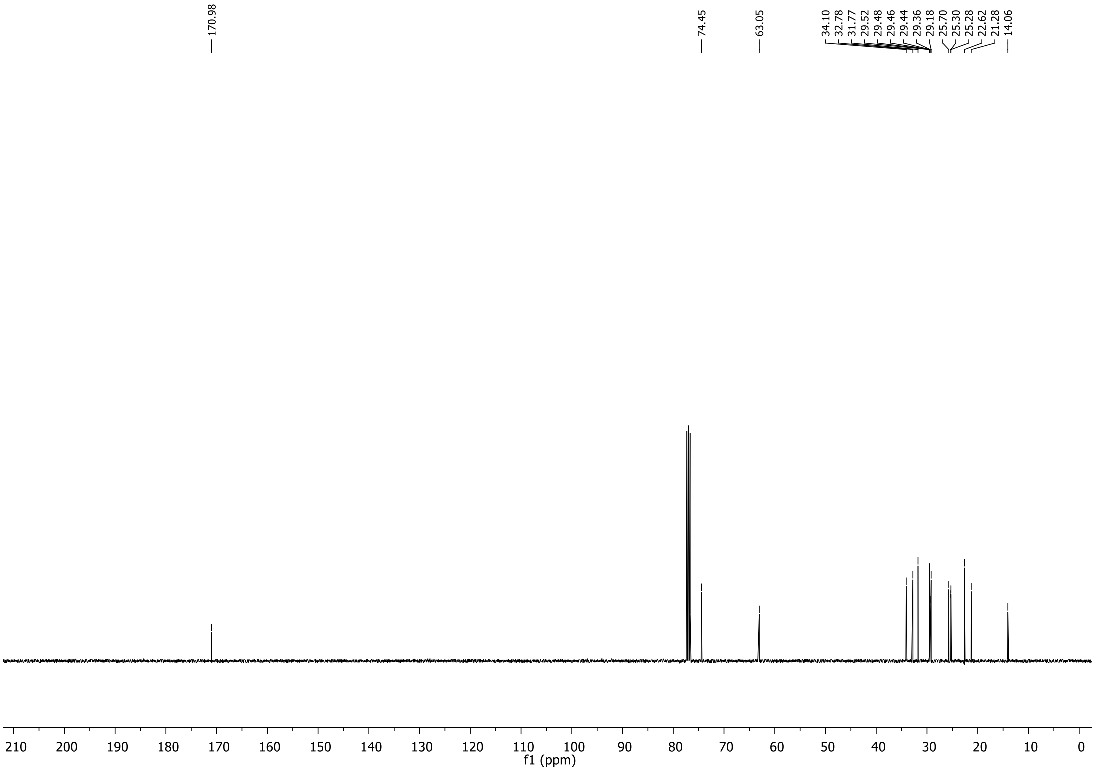
170.98
74.45
63.05
34.10
32.78
31.77
29.52
29.48
29.46
29.44
29.36
29.18
25.70
25.30
25.28
22.62
21.28
14.06
f1 (ppm)

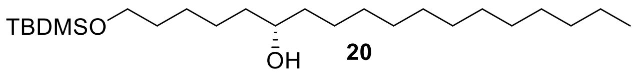
TBDMSO
OH

20

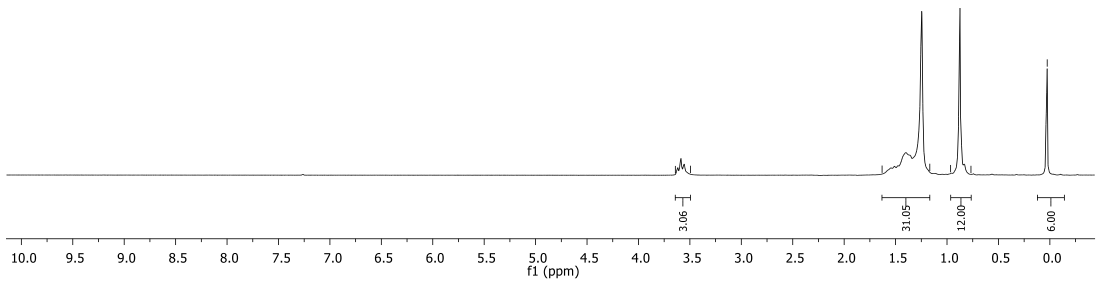
3.64
3.49
1.63
1.17
0.96
0.77
0.03
3.06
31.05
12.00
6.00
10.0 9.5 9.0 8.5 8.0 7.5 7.0 6.5 6.0 5.5 5.0 4.5 4.0 3.5 3.0 2.5 2.0 1.5 1.0 0.5 0.0
f1 (ppm)

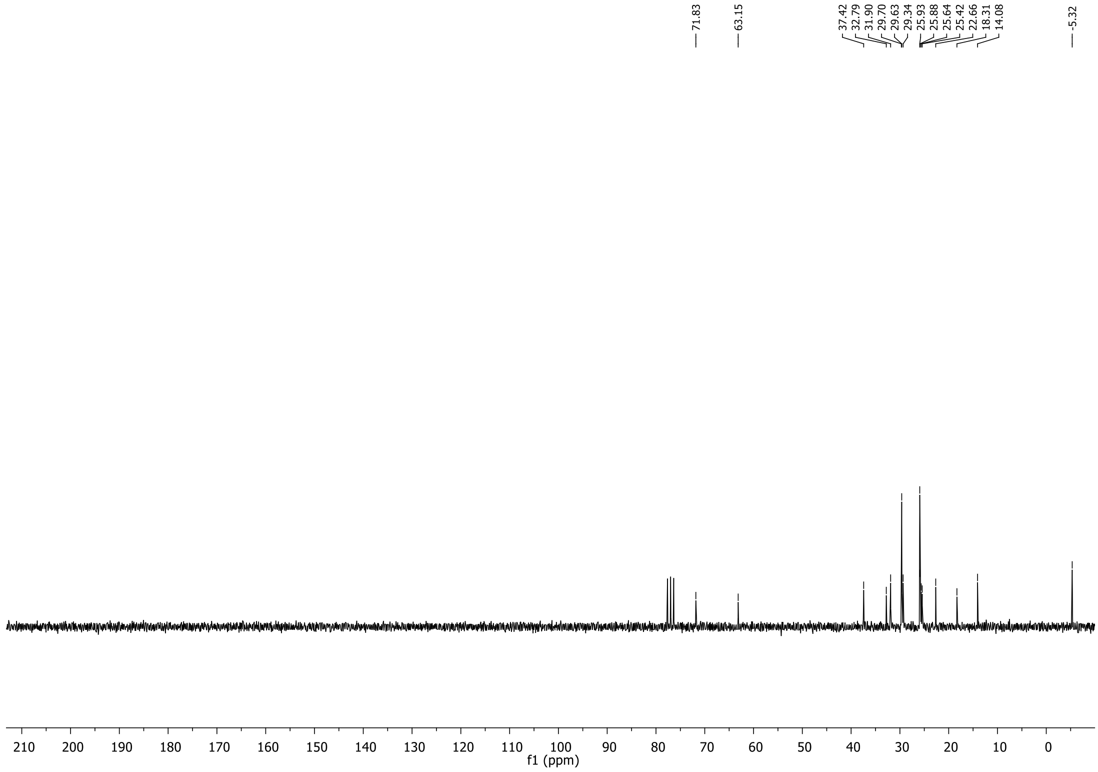
71.83
63.15
37.42
32.79
31.90
29.70
29.63
29.34
25.93
25.88
25.64
25.42
22.66
18.31
14.08
-5.32
210 200 190 180 170 160 150 140 130 120 110 100 90 80 70 60 50 40 30 20 10 0
f1 (ppm)
